# A data-driven modeling approach to identify disease-specific multi-organ networks driving physiological dysregulation

**DOI:** 10.1371/journal.pcbi.1005627

**Published:** 2017-07-21

**Authors:** Warren D. Anderson, Danielle DeCicco, James S. Schwaber, Rajanikanth Vadigepalli

**Affiliations:** Daniel Baugh Institute for Functional Genomics and Computational Biology, Department of Pathology, Anatomy, and Cell Biology, Sidney Kimmel Medical College, Thomas Jefferson University, Philadelphia, PA, USA; University of Michigan, UNITED STATES

## Abstract

Multiple physiological systems interact throughout the development of a complex disease. Knowledge of the dynamics and connectivity of interactions across physiological systems could facilitate the prevention or mitigation of organ damage underlying complex diseases, many of which are currently refractory to available therapeutics (e.g., hypertension). We studied the regulatory interactions operating within and across organs throughout disease development by integrating *in vivo* analysis of gene expression dynamics with a reverse engineering approach to infer data-driven dynamic network models of multi-organ gene regulatory influences. We obtained experimental data on the expression of 22 genes across five organs, over a time span that encompassed the development of autonomic nervous system dysfunction and hypertension. We pursued a unique approach for identification of continuous-time models that jointly described the dynamics and structure of multi-organ networks by estimating a sparse subset of ∼12,000 possible gene regulatory interactions. Our analyses revealed that an autonomic dysfunction-specific multi-organ sequence of gene expression activation patterns was associated with a distinct gene regulatory network. We analyzed the model structures for adaptation motifs, and identified disease-specific network motifs involving genes that exhibited aberrant temporal dynamics. Bioinformatic analyses identified disease-specific single nucleotide variants within or near transcription factor binding sites upstream of key genes implicated in maintaining physiological homeostasis. Our approach illustrates a novel framework for investigating the pathogenesis through model-based analysis of multi-organ system dynamics and network properties. Our results yielded novel candidate molecular targets driving the development of cardiovascular disease, metabolic syndrome, and immune dysfunction.

## Author summary

Complex diseases such as hypertension often involve maladaptive autonomic nervous system control over the cardiovascular, renal, hepatic, immune, and endocrine systems. We studied the pathogenesis of physiological homeostasis by examining the temporal dynamics of gene expression levels from multiple organs in an animal model of autonomic dysfunction characterized by cardiovascular disease, metabolic dysregulation, and immune system aberrations. We employed a data-driven modeling approach to jointly predict continuous gene expression dynamics and gene regulatory interactions across organs in the disease and control phenotypes. We combined our analyses of multi-organ gene regulatory network dynamics and connectivity with bioinformatic analyses of genetic mutations that could regulate gene expression. Our multi-organ modeling approach to investigate the mechanisms of complex disease pathogenesis revealed novel candidates for therapeutic interventions against the development and progression of complex diseases involving autonomic nervous system dysfunction.

## Introduction

Complex disease conditions characterized by co-morbidities involve pathological dysregulation that evolves across multiple organ systems and over time. Thus, a holistic approach is required to deconvolve the spatiotemporally distributed mechanisms of multifactorial disease pathogenesis at the tissue, cellular, and molecular levels of analysis. From this systems perspective, time-series analyses of multiple organs are essential to determining the biological mechanisms of disease progression [1–4].

New insights into complex disease mechanisms have been derived from analyses of gene expression across multiple human organs [5–7]. The temporal dynamics of human multi-organ gene expression profiles have provided insight into the distributed mechanisms of diseases including hypertension [8]. Such studies of animal models can be used to study disease pathogenesis by examining time points both before and after disease onset. Existing studies have provided valuable information regarding the contributions of various organs to cardiovascular disease [9, 10], but the absence of global longitudinal studies precludes our understanding of the molecular mechanisms underlying disease pathogenesis.

Even when time-series data are available, complications with conventional analysis approaches often preclude new insights. Common statistical methods that account for time as a categorical variable often fail to detect significant differences between the dynamics of phenotypes, necessitating an explicit consideration of time as a continuous variable in statistical analysis [11]. Analytical techniques available to infer the network interactions underlying the gene expression dynamics require extensive experimental assessments of responses to targeted gene perturbations [12]. The utility of combining time-series analysis with network-based approaches has been demonstrated extensively in developmental biology [13, 14], immunology [15–17], neural systems [18, 19], and critical care medicine [20, 21]. Interactions between dynamics and structure have also been studied in mechanical, electrical, telecommunication, social, and economic networks [22]. However, such approaches have been relatively underutilized in controlled studies of organismal physiology [23].

It has been proposed that a triangular pattern of positive feedback—with vertices representing autonomic nervous system (ANS) activity, systemic inflammation (INF), and renin-angiotensin system (RAS) signaling—underlies the pathogenesis of cardiovascular dysfunction in hypertension [24]. Accordingly, cardiovascular function can be modulated by perturbations of peripheral T-cells [25], bone marrow cells [26], renal [27, 28] and hepatic systems [29], the adrenal gland [30], as well as neurons and glial cells in the brain [31, 32]. Because of the positive feedback interactions amongst physiological systems involved in cardiovascular regulation [24], it is difficult to determine causal mechanisms of disease pathogenesis. The attribution of disease mechanisms can be facilitated by the temporal reconstruction of events underlying the multi-organ system’s evolution toward a pathological state [23, 33]. We performed such a temporal reconstruction by integrating experimental measurements with novel data-driven modeling and network analysis.

We profiled the temporal dynamics of ANS, INF, and RAS gene expression in the adrenal gland, brainstem, kidney, liver, and left ventricular muscle to characterize the multi-organ contributions to disease etiology. We utilized a rat model of complex disease—involving cardiovascular, metabolic, and cognitive impairments—in which autonomic dysfunction is believed to be a key factor in controlling the development and persistence of the disease state [24, 34]. Hence, we refer to this model as an “autonomic dysfunction” phenotype. Extensive evidence supports the relevance of this animal model to the pathogenesis of human hypertension. For instance, pharmacological perturbations of ANS and RAS signaling, and surgical manipulations of ANS signaling, exert anti-hypertensive effects in both humans and the autonomic dysfunction model. Other commonalities include elevated inflammation and elevated sympathetic activity that appears to precede hypertension in both humans and rats [24, 35–38]. Hence, it is plausible that the autonomic dysfunction animal model recapitulates key features of human disease pathogenesis. We applied a robust technique for system identification to estimate the strength, direction, and sign of interactions amongst genes within and between organs. We utilized a *Hartley Modulating Function (HMF)*-based system identification approach, which allowed us to estimate both continuous mathematical models of gene expression dynamics and corresponding network models of multi-organ gene regulatory interactions. We analyzed the model structure and simulation results to test whether the temporal dynamics and gene regulatory interactions were globally affected during the pathogenesis of autonomic dysfunction. We analyzed the model to identify disease-specific network motifs associated with aberrant temporal dynamics. We were interested in identifying whether the gene expression dynamics and network interactions were prominently dysregulated in particular organs, suggesting an anatomical basis for disease development. We further investigated whether single nucleotide variants were significantly associated with the transcription factor binding sites upstream of ANS, INF, and RAS genes. Our analyses utilized a novel investigative framework to identify new candidate therapeutic targets for ANS-related diseases based on aberrant expression dynamics and network interactions involving genes in multiple organs.

## Materials and methods

### Ethics statement

All experimental work was performed according to protocols approved by the Thomas Jefferson University Institutional Animal Care and Use committee.

### Animal procedures

All protocols were approved by the Thomas Jefferson University (TJU) Institutional Animal Care and Use Committee. Study subjects included male rats from the Spontaneously Hypertensive Rat (SHR/NHsd) and Wistar Kyoto (WKY/NHsd) strains, corresponding to autonomic dysfunction and control phenotypes, respectively. Rats were purchased from Harlan Laboratories and experimental procedures were carried out one week following animal arrival at our facility. All animals were housed socially in the TJU animal facility. The facilities were maintained at constant temperature and humidity with 12/12 hour light cycles (lights on at Zeitgeber time = 0). We harvested organ tissues at five time points: 4, 6, 8, 12, and 16 weeks of age. Rats were humanely sacrificed via rapid decapitation. CNS tissue was excised and the brainstem was isolated in ice-cold artificial cerebral spinal fluid (10mM HEPES; 140mM NaCl; 5mM KCl; 1mM MgCl_2_; 1mM CaCl_2_; 24mM D-glucose; pH = 7.4). We simultaneously harvested the adrenal gland, kidney, liver, and left ventricle of the heart. Tissue samples were flash frozen and stored at -80°C. Our original study was designed to include 50 animals (2 genotypes, 5 time points, 5 replicates). One animal deceased prior to the designated time point for organ harvest. Thirty-five animals were included in our study and 2–5 organ samples per strain were obtained at each time point for organs other than the brainstem (S1A Fig); 12 week brainstem tissues (from n = 10 animals) were not included in the present study as these samples were utilized for a parallel study that precluded the gene expression analysis employed here. Five other animals were excluded from our study prior to performing qPCR analysis because either the respective RNA did not pass our quality criteria (see below) or because the tissue was used for other purposes. S1A Fig shows the tissue samples included in our study for each animal.

### Molecular biology

Total RNA was extracted from 10–50 mg tissue samples using the Direct-Zol RNA extraction kit, which captures all RNA greater than 18 nucleotides in length (ZYMO Research, Irvine, CA). Samples were DNAse treated and stored at -80°C. Concentration and integrity were assessed with a spectrophotometer (ND-1000 from NanoDrop, Philadelphia, PA). RNA samples with 260/280 (nm/nm) ratio <1.8 and 260/230 ratio 1.8–2.0 were purified with RNA Clean and Concentrator-100 (ZYMO Research, Irvine, CA). High-throughput PCR was implemented as described previously [39, 40]. Intron-spanning PCR primers were designed for 24 assays (see Table 1 in S1 Text). For each sample, 30 ng of total RNA was used. The standard BioMark protocol (Fluidigm, South San Francisco, CA) was employed to reverse transcribe and pre-amplify cDNA samples for 12 cycles using TaqMan PreAmp Master Mix based on the manufacturer’s protocol (Applied Biosystems, Foster City, CA). The qPCR reactions were performed using a 192.24 BioMark Rx Dynamic Array for multiplex gene expression measurement (Fluidigm, South San Francisco, CA). Quantitative PCRs were implemented with 30 cycles (95°C for 15s, 70°C for 5s, 60°C for 60s).

### Data processing

We quantified qPCR products by determining threshold cycle (Ct) values. We designed our study with *Actb* and *Gapdh* assays as potential reference genes for normalization. To determine whether these assays were appropriate for normalization, we assessed the stability of these genes across samples, as in our previous studies [39], based on a well established method for evaluation of putative reference genes [41]. Our analysis revealed that *Actb* and *Gapdh* expression profiles were highly variable across samples, as has been shown previously. No single gene showed consistent stability across all samples. However, the median Ct across all genes was stable across samples, indicating superior utility of the median Ct as a ‘pseudo reference gene’ for data normalization (S1B Fig). Hence, we normalized the raw Ct data based on median expression levels, which were considered to represent reference gene expression levels. For each sample (s) obtained from a specific organ (r) at a specific time point (t), we subtracted the median Ct computed across all genes (g) in that organ: $\Delta Ct_{rg}^{s}\left(t\right)=Ct_{rg}^{s}\left(t\right)-med\left(Ct_{r}^{s}\left(t\right)\right)$ where *med*(.) is the median of the argument. We next centered the data for comparison across genes based on the median expression level across all samples for each gene: $\Delta \Delta Ct_{rg}^{s}\left(t\right)=\Delta Ct_{rg}^{s}\left(t\right)-med\left(\Delta Ct_{rg}\left(t\right)\right)$. We used −ΔΔ*Ct* values for analyses of gene expression. We omitted *Actb* and *Gapdh* from all subsequent analysis due to ambiguity in the functional interpretation of the results. Missing data based on our QC analysis were rare (median = 1.8%, sd = 10% of samples per gene with NA values; median = 4.2%, sd = 9.1% of genes per sample with NA values). Thus, missing data were imputed according to established approaches [6, 42] by replacing missing values with the mean across 10 samples with most similar expression profiles according to Euclidean distance using the *impute* package in R [43]. Note that we did not impute Brainstem data at age = 12 weeks because we did not obtain the gene expression data from the brainstem samples at this time point. Both raw Ct and normalized data are available (S1 and S2 Files). To examine whether specific samples imparted systematic biases in our results, we implemented Principal Components Analysis in R using the *princomp* function.

### Timeseries analysis

To test whether the temporal dynamics of gene expression differed between autonomic dysfunction and control phenotypes, we applied the *Optimal Discovery Procedure* (ODP) using the *EDGE* package in R [44]. Temporal profiles were modeled as natural cubic splines which connect a series of smooth polynomials between knots defined by the degrees of freedom for the spline fit [11, 45] (function *ns* with df = 3 in the *splines* package for R [46]). The ODP analysis involved a comparison of a null model to an alternative model. The null model was characterized by a single spline fit to the aggregated autonomic dysfunction and control time-series data for each gene. The alternative model consisted of two splines fit to the respective phenotypes. For each gene, errors between data points and fitted values were summed and squared for the null model (*SS*^0^) and the alternative model (*SS*^*A*^). An analogue of the conventional *F* statistic was computed to evaluate the goodness of fit obtained for the null versus alternative model: *F* = (*SS*^0^ − *SS*^*A*^)/*SS*^*A*^. The estimated distribution for this statistic was utilized to compute an estimate of the probability of the alternative model under the null hypothesis (p value) with correction for multiple testing (q value). Complete details can be found in [11, 44].

### Dynamic network and continuous-time system identification from discrete time-series data

**Fig 1:**
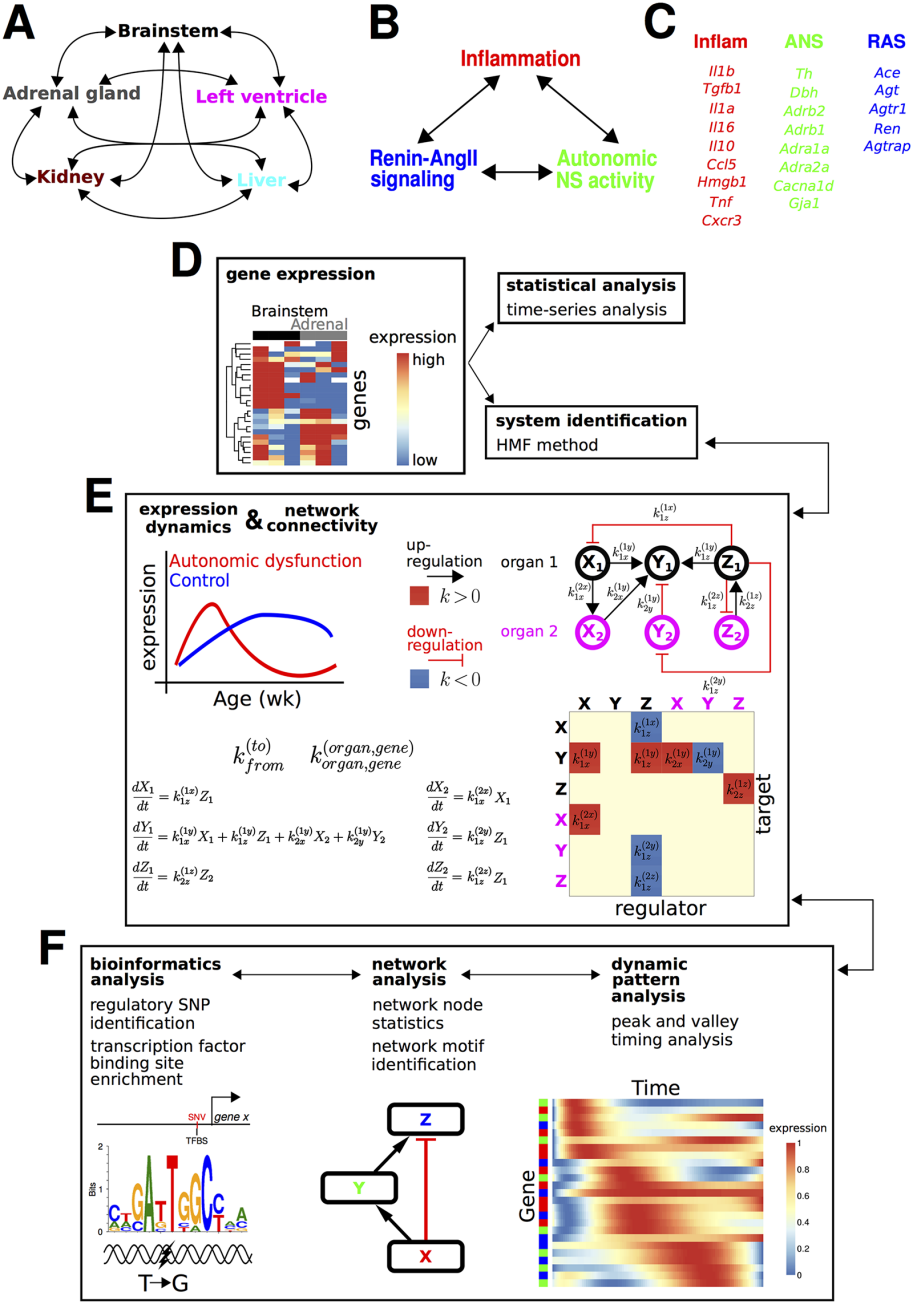
Conceptual, experimental, and analytic framework for examining multi-organ pathogenesis of autonomic function. (A, B) The etiology of autonomic dysfunction involves (A) multiple organs in which positive feedback processes involve (B) inflammatory mediators, renin-angiotensin signaling, and sympathetic activity. (C) A number of well studied genes underlie the molecular basis of maladaptive network feedback processes. (D-F) Analysis pipeline for examination of expression patterns of the genes listed in (C) in multiple organs listed in (A). (D) Gene expression measurements were obtained and the data were analyzed using time series statistics and novel network identification approaches. (E) The network identification analysis reconstructed gene expression dynamics and gene regulatory interactions underlying autonomic dysfunction and control phenotypes. (F) Bioinformatic analyses were integrated with analyses of network structure and dynamics to generate novel hypotheses regarding the molecular mechanisms underlying autonomic dysfunction.

We scaled the data to the range (0, 1) prior to implementing the *HMF method*. To implement this scaling for expression profile *E*, we applied the following transformation: *E*_*scaled*_ = (*E* − *min*(*E*))/(*max*(*E*) − *min*(*E*)). We use *E* to represent *E*_*scaled*_ in the context of our system identification studies. The following description details the basic theory and procedure underlying the system identification approach using *Hartley Modulating Functions (HMF)*. A signature attribute of this approach is that interaction coefficients can be estimated that jointly describe network dynamics and structure. From a mathematical perspective, a principal advantage of the HMF-based system identification approach is that it obviates the need to compute temporal derivatives of the raw data [47, 48]. Instead, the interaction coefficients *k* (see Fig 1D) are determined by estimating inner products between both the expression data (and the derivatives thereof) and a set of basis functions—the carefully chosen *Hartley modulating functions*—and approximating these inner products using the *Hartley transform* to transform the data into the frequency domain. This procedure facilitates the accurate and robust identification of continuous-time models from discretely sampled data, another principal advantage of the *HMF method*, as we have demonstrated previously [48].

Furthermore, our approach entailed the use of powerful regularization techniques that mitigate against overfitting the interaction coefficients [45]. Whereas frequency domain transformations of data have been previously been employed to implement systems identification, these approaches relied on optimization-based estimates of the interaction coefficients [49]. In contrast, using *HMF method*, we directly estimated the interaction coefficients via regularized regression. Thus, our approach, in principle, can overcome difficulties in parameter estimation that result from non-convex solution spaces characterized by local minima [50].

The remainder of this section starts with a description of the mathematical underpinnings and implementation details underlying our use of the *HMF method* to identify multi-organ gene regulatory networks. Following this description, we detail further analyses that demonstrate the robustness of our approach.

The expression level *E* of gene *g* in organ *r* at time *t* was modeled as follows for a data set with samples obtained between time *t* = 0 and time *t* = *T*:

$$\begin{array}{c} \frac{d}{dt}E_{rg}\left(t\right)=\sum_{i}^{N_{r}}\sum_{j}^{N_{g}}k_{ij}^{\left(rg\right)}E_{ij}\left(t\right)-\gamma_{rg}E_{rg}\left(t\right) \end{array}$$

where *N*_*r*_ is the number of organs and *N*_*g*_ is the number of genes. The degradation coefficient for *E*_*rg*_ is referred to as *γ*_*rg*_. For simplicity, we avoided using the degradation term explicitly such that degradation was implicitly incorporated in *k*_*ij*_ for *i* = *r* and *j* = *g*:

$$\begin{array}{c} \frac{d}{dt}E_{rg}\left(t\right)=\sum_{i}^{N_{r}}\sum_{j}^{N_{g}}k_{ij}^{\left(rg\right)}E_{ij}\left(t\right) \end{array}$$ (1)

The full network can be compactly expressed in matrix form as

$$\begin{array}{c} \left[\begin{array}{c} \frac{d}{dt}E_{11}\\ \frac{d}{dt}E_{12}\\ \vdots \\ \frac{d}{dt}E_{N_{r}N_{g}} \end{array}\right]=\left[\begin{array}{cccc} k_{11}^{\left(11\right)} & k_{12}^{\left(11\right)} & \cdots & k_{N_{r}N_{g}}^{\left(11\right)}\\ k_{11}^{\left(12\right)} & k_{12}^{\left(12\right)} & \cdots & k_{N_{r}N_{g}}^{\left(12\right)}\\ \vdots & \vdots & \ddots & \vdots \\ k_{11}^{\left(N_{r}N_{g}\right)} & k_{12}^{\left(N_{r}N_{g}\right)} & \cdots & k_{N_{r}N_{g}}^{\left(N_{r}N_{g}\right)} \end{array}\right]\left[\begin{array}{c} E_{11}\\ E_{12}\\ \vdots \\ E_{N_{r}N_{g}} \end{array}\right] \end{array}$$

with the following simplified representation:

$$\begin{array}{c} \frac{d}{dt}E\left(t\right)=KE\left(t\right) \end{array}$$ (2)

Note that that parameter matrix **K** from Eq (2) is equivalent to the Jacobian matrix corresponding to this linear system: **J** = [*J*_*ij*_] where *J*_*ij*_ = ∂*f*_*i*_/∂*E*_*j*_, *f*_*i*_ = *dE*_*i*_/*dt*, and (*i*, *j*) each refer to a particular gene-organ combination. Thus, this matrix gives the influence of a gene in column *j* on a gene in row *i*.

We estimated the interaction coefficients *k* by applying the *HMF method* [47, 48]. This method entails the multiplication of Eq (1) by *M* different modulation functions *ϕ*_*m*_ (*m* = 1, 2,…, *M*). Integrating these products gives the following relation:

$$\begin{array}{c} \int_{0}^{T}\phi_{m}\left(\frac{d}{dt}E_{rg}\right)dt=\sum_{i}^{N_{r}}\sum_{j}^{N_{g}}\left(\int_{0}^{T}\phi_{m}E_{ij}dt\right)k_{ij}^{\left(rg\right)} \end{array}$$ (3)

where *ϕ*_*m*_ = *f*(*t*) is chosen such that $\frac{d}{dt}\phi_{m}\left(t\right)$ = 0 for *t* = 0 and *t* = *T*. Note that the times *t* = 0 and *t* = *T* corresponding to sampled ages of 4 and 16 weeks, respectively, such that *T* = 12 in our computations. The modulating functions *ϕ* are chosen as follows:

$$\phi_{m}\left(t\right)=\sum_{j=0}^{n}{\left(-1\right)}^{j}\left({}_{k}^{n}\right)cas\left(\left(n+m-j\right)\omega_{0}t\right)$$

where *n* is the order of the highest derivative of the system described by Eq (1) (i.e., n = 1), *cas*(*x*) = *sin*(*x*) + *cos*(*x*), and $\omega_{0}=\frac{2\pi }{T}$. The integrals on the right and left hand sides of Eq (3) can be estimated using the *Hartley transform* [51], the m-th HMF spectral component of gene expression profile *E*(*t*), and the HMF spectra for the i-th derivative of *E*(*t*) [47]. These computations are defined respectively as follows:

$$\begin{array}{c} H_{rg}\left(\omega \right)=\int_{0}^{T}E_{rg}\left(t\right)cas\left(\omega t\right)dt \end{array}$$ (4)

$${\bar{H}}_{rg}\left(m\omega_{0}\right)=\sum_{j=0}^{n}{\left(-1\right)}^{j}\left({}_{j}^{n}\right)H_{rg}\left(\left(n+m-j\right)\omega_{0}\right)$$ (5)

$$\begin{array}{c} {\bar{H}}_{rg}^{i}\left(m\omega_{0}\right)=\sum_{j=0}^{n}f_{1}f_{2}f_{3} \end{array}$$ (6)

where

f1=(-1)jnjddtcasiπ2

$$\begin{array}{c} f_{2}={\left(n+m-j\right)}^{i}\omega_{0}^{i} \end{array}$$

$$\begin{array}{c} f_{3}=H_{rg}\left({\left(-1\right)}^{i}\left(n+m-j\right)\omega_{0}\right) \end{array}$$

Importantly, given a solution to Eq (4), numerical solutions to Eqs (5 and 6) can be obtained. The numerical solutions to Eqs (5 and 6) can be used to compute the interaction coefficients based on the following relations:

$$\begin{array}{c} {\bar{H}}_{rg}\left(m\omega_{0}\right)=\int_{0}^{T}\phi_{m}E_{rg}dt \end{array}$$ (7)

$$\begin{array}{c} {\bar{H}}_{rg}^{i}\left(m\omega_{0}\right)=\int_{0}^{T}\phi_{m}\left(\frac{d^{i}}{dt^{i}}E_{rg}\right)dt \end{array}$$ (8)

Then Eq (2) can be written as follows

$$\begin{array}{c} {\bar{H}}_{rg}^{1}\left(m\omega_{0}\right)=\sum_{i}^{N_{r}}\sum_{j}^{N_{g}}{\bar{H}}_{rg}\left(m\omega_{0}\right)k_{ij}^{\left(rg\right)} \end{array}$$ (9)

where the only unknowns are the interaction coefficients, which can be determined by linear regression. However, Eq (4) must be computed first. Following our previous work [48], we computed Eq (4) by linearly interpolating between average gene expression values at adjacent time points and analytically evaluating the integral:

$$\begin{array}{c} \int_{t_{i}}^{t_{f}}E(t)cas(\omega t)dt=\int_{t_{i}}^{t_{f}}(mt+b)cas(\omega t)dt \end{array}$$

$$\begin{array}{c} m=\frac{E\left(t_{f}\right)-E\left(t_{i}\right)}{t_{f}-t_{i}},b=E\left(t_{i}\right)-mt_{i} \end{array}$$

Then we computed the values in Eqs (5 and 6) given *n* = *i* = 1 [48]. Note that the analytical solution to the integral in Eq (4) evaluates to zero for *n* + *m* − *j* = 0 (*m* = −1, *j* = 0 and *m* = 0, *j* = 1). However, allowing Eq (4) to be zero resulted in poor fits of the model to the data. For these cases, we arbitrarily set *n* + *m* − *j* = *ϵ* with *ϵ* = 10^−6^. This choice of *ϵ* resulted in robust model fits as detailed below.

The set of interaction coefficients can be obtained for interactions regulating the expression dynamics of each organ/gene combination by solving Eq (9) using a range of *m* values:

$$\begin{array}{c} {\bar{H}}_{rg}^{1}\left(m_{1}\omega_{0}\right)=\sum {}_{i}^{N_{r}}\sum {}_{j}^{N_{g}}{\bar{H}}_{rg}\left(m_{1}\omega_{0}\right)k_{ij}^{\left(rg\right)}\\ {\bar{H}}_{rg}^{1}\left(m_{2}\omega_{0}\right)=\sum {}_{i}^{N_{r}}\sum {}_{j}^{N_{g}}{\bar{H}}_{rg}\left(m_{2}\omega_{0}\right)k_{ij}^{\left(rg\right)}\\ \vdots \\ {\bar{H}}_{rg}^{1}\left(m_{M}\omega_{0}\right)=\sum {}_{i}^{N_{r}}\sum {}_{j}^{N_{g}}{\bar{H}}_{rg}\left(m_{M}\omega_{0}\right)k_{ij}^{\left(rg\right)} \end{array}$$

In matrix notation, this relation can be expressed as

$$\begin{array}{c} y_{rg}=X_{rg}\beta^{\left(rg\right)} \end{array}$$ (10)

where **y** is a column vector of ${\bar{H}}_{rg}^{1}$ terms in which each row entry is computed with a different *m* value, **X** is a matrix of ${\bar{H}}_{rg}$ terms with a row for each *m* value and a column for each term corresponding to a particular organ/gene combination, and *β* is a vector of interaction coefficients with the same length as the number of columns in **X**. Solving the linear system for *β* provides interaction coefficients that determine the dynamic regulation of a gene in a given organ, based on the expression profiles of all genes in all organs. However, we wanted to perform system identification for gene regulatory networks spanning multiple organs and genes. To globally estimate the entire set of interaction coefficients using the *HMF method*, a matrix of the form Eq (11) was utilized. The full matrix was established using instances of Eq (10) for each organ/gene combination considered in the system:

$$\begin{array}{c} \left[\begin{array}{c} y_{11}\\ y_{12}\\ \vdots \\ y_{N_{r}N_{g}} \end{array}\right]=\left[\begin{array}{cccc} X_{11} & 0 & \cdots & 0\\ 0 & X_{12} & \cdots & 0\\ \vdots & \vdots & \ddots & \vdots \\ 0 & 0 & \cdots & X_{N_{r}N_{g}} \end{array}\right]\left[\begin{array}{c} \beta^{\left(11\right)}\\ \beta^{\left(12\right)}\\ \vdots \\ \beta^{\left(N_{r}N_{g}\right)} \end{array}\right] \end{array}$$ (11)

where **0** represents a matrix of zeros with the same dimensionality as **X**. In this formulation, **y**_11_ = **X**_11_ *β*^(11)^, **y**_12_ = **X**_12_ *β*^(12)^, and **y**_*N*_*r*_*N*_*g*__ = **X**_*N*_*r*_*N*_*g*__ *β*^(*N*_*r*_*N*_*g*_)^. Thus, the solution to Eq (11) provides a global fit to the interaction network including all assayed genes in all sampled organs. The regression problem can be described compactly as **y** = **X** *β* and solved using linear regression. To circumvent overfitting of the model, we applied well established regularization techniques in which the regression coefficients were determined by solving an optimization problem with the following objective function:

$$\begin{array}{c} J_{reg}=\underset{\beta }{min}\left(||y-{X\beta ||}^{2}+\lambda \left({\alpha ||\beta ||}_{1}+{\left(1-\alpha \right)||\beta ||}_{2}^{2}\right)\right) \end{array}$$ (12)

where ||*x*||_1_ = ∑|*x*| and ${\left||x|\right|}_{2}=\sqrt{{\sum |x|}^{2}}$. The regularization parameters *α* ∈ [0, 1] and *λ* ∈ [*λ*_*min*_, *λ*_*max*_] (see below) impose sparsity on the network by forcing the interaction coefficients towards zero [45]. This form of regularization is known as the ‘*elastic net*’ [52], where the ||*β*||_1_ term represents the ‘*lasso*’ penalty [53] and ||*β*||_2_ term represents the ‘*ridge*’ penalty [54]. Thus, *α* weights the *lasso* penalty and (1−*α*) weights the *ridge* penalty. The *elastic net* formalism exhibits positive attributes of both regularization techniques with respect to enhancement of network interpretation, based on sparsity of connectivity, and augmentation of prediction accuracy [45].

We performed network identification as described above using a range of *m* value sets. The regression problem represented by Eq (11) requires that the number of *m* values exceeds the number of interaction coefficients, *N*_*r*_ ⋅ *N*_*g*_. Each *m* value set had the form *m* = 0, ±1, ±2, …, ±(*M* − 1), ±*M*, where *M* was varied between $M_{min}=\frac{N_{r}\;\cdot \;N_{g}}{2}$ and $M_{max}=\frac{N_{samples}}{2}$ [48]. Initial simulations showed that the regression results were not sensitive to the range of the *m* values, given *M* ≥ *M*_*min*_. We selected ten sets of *m* values that were evenly spaced within the *M* range. For each of the ten *m* value sets, we performed the regression analysis for *α* = 0, 0.2, 0.4, 0.6, 0.8, 1, and we used 10 *λ* values for each *α*. The *λ* values were also varied over a range. The *λ* range was bounded by *λ*_*max*_, the minimal *λ* value associated with a particular *α* that resulted in all zero coefficients. That is, for *λ* = *λ*_*max*_ there was no network connectivity. The *λ* values were incrementally varied from *λ*_*min*_ = *λ*_*max*_ × 10^−4^ to *λ*_*max*_ on a logarithmic scale according to the default functionality of the *glmnet* package used for *elastic net* regression in R [46, 55].

Optimal solutions to the regularized regression problems (11 and 12) were determined based on simulation results. We simulated the identified network models (2) using MATLAB’s *ode*45 and *ode*15*s* functions, or with R using the *lsoda* function from the *deSolve* package [56]. Differences in simulation results, with respect to the choice of numerical integrator, were not visually detectable. To select the optimal fits, we initially considered the sum of squared residuals as an objective function for minimization, $\sum {\left(\hat{y}-\bar{y}\right)}^{2}$, where $\hat{y}$ is the simulation result and $\bar{y}$ is the average for the corresponding gene expression data set. However, according to this objective, the best fits could be those with all zero coefficients. Hence, we revised our objective to penalize fits with low variability:

$$\begin{array}{c} J_{sim}=\frac{\sum {\left(\hat{y}-\bar{y}\right)}^{2}}{Var(\hat{y})+\epsilon } \end{array}$$ (13)

and set *ϵ* = 10^−10^ (the choice of *ϵ* did not make a detectable difference in the selection of the best fit).

Molecular networks underlying the physiology of blood pressure control have been shown to be distinct for autonomic dysfunction versus control [40]. For all network reconstructions, we considered autonomic dysfunction and control phenotypes separately. We initially performed our system identification analysis of a network with all organs (*N*_*r*_ = 5) and genes (*N*_*g*_ = 22) with (*N*_*r*_ ⋅ *N*_*g*_)^2^ = 12,100 possible connections. We implemented 600 iterations of the system identification algorithm with distinct combinations of the *m* value range (n = 10), *λ* value (n = 10), and *α* value (n = 6). In general, for both phenotypes, small *λ* values were associated for good fits (i.e., *J*_*sim*_ ∼ *min*(*J*_*sim*_)) irrespective of the *α* value for *α* > 0 (S2 Fig). For the control, *J*_*sim*_ = *min*(*J*_*sim*_) for *α* = 0.2 at *λ* = 6.5⋅10^−5^ (221 *m* values). For autonomic dysfunction, *J*_*sim*_ = *min*(*J*_*sim*_) for *α* = 0.4 at *λ* = 8.3⋅10^−7^ (111 *m* values). Based on these pilot studies, further analyses were implemented with ten *λ* values, ten sets of *m* values, and *α* = 0.2.

### System identification robustness analysis

To evaluate the robustness of the *HMF Method*, we compared the ‘best fit’ network with multiple subnetworks characterized by *log*(*J*_*sim*_) < 10 (see S2 Fig). For these comparisons, we considered interaction coefficients that were larger in absolute magnitude than two standard deviations from the median (*median* ∼ 0 in all cases). Further, we only considered the coefficients for which $|k|>|2{\hat{\sigma }}_{k}-med\left(k\right)|$ in each phenotype-specific best fit network and comparison network, and analyses were completed for coefficients that met this criterion for both the best fit network and the comparison network. Spearman rank correlation coefficients were determined and we investigated whether the coefficient sign was sensitive to the regularization parameters using the Fisher's exact test (FET). For the FET analysis, we computed the sign (i.e., +1 or −1) of the coefficients considered in the correlation analysis. The contingency table illustrated in S3 Fig was formulated for the FET. Both Spearman rank and FET p-values were corrected for multiple testing according to the Benjamini-Hochberg method using the *qvalue* package [57]. We also computed the odds ratio, based on the same data, to quantify the degree of agreement between networks (S3 Fig).

To further evaluate robustness, we examined graph theoretic metrics. A path through a network consists of the sequence of edges between two nodes or vertices. The shortest path length refers to the minimal number of edges connecting two nodes, and the average path length < ℓ > is the mean of all shortest paths between every pair of nodes. This measure is an index of the efficiency with which the network can be navigated. The clustering coefficient *C*_*i*_ generally quantifies the connectivity among all nodes connected to node *i*. The number of nodes with links to node *i* is *n*_*i*_ and the number of links amongst the *n*_*i*_ nodes is *n*_*c*_: *C*_*i*_ = 2*n*_*c*_/(*n*_*i*_ ⋅ (*n*_*i*_−1)) [58]. We computed a variant of this measure, the global clustering coefficient, which is the number of closed node triplets (i.e., ‘triangles’) divided by the total number of connected triplets. The global clustering coefficient is also known as the ‘transitivity’ metric [59]. Finally, we assessed the distribution of degrees *k*_*c*_; that is, the distribution for the number of edges connecting a node to its neighbors, which follows a power law of the following form for many biological networks: $P\left(k_{c}\right)∼k_{c}^{-\gamma }$. This distribution gives the probability that a given node has *k*_*c*_ connections [58, 60]. To characterize the degree distribution, we fit a power law to the vector of degree frequencies and utilized a Kolmogorov-Smirnov (K-S) test of the null hypothesis that the data were distributed according to the power law. The fit returned an estimate of *γ* and a p-value indicating the probability of the test statistic given the null hypothesis. Thus, high p-values indicate the absence of evidence in support of the conclusion that the graph under consideration does not exhibit a power law degree distribution. Network analyses were implemented using the *igraph* package for R [59].

### Dynamic pattern analysis

We assessed the patterns of gene expression dynamics across and within organs by temporally ordering the expression profiles identified using the *HMF method*. We used temporal ordering schemes based on peak timing and valley timing, which were established as follows. The dynamic profiles *E*_*i*_ of each gene *i* were scaled to *E*^*sc*^ ∈ [0, 1]. We then determined all local extrema for the scaled waveform *E*^*sc*^. We refer to local maxima as peaks and local minima as valleys, with associated times denoted as *t*_*p*_ and *t*_*v*_. Putative peaks and valleys ($\hat{t_{p}}$ and $\hat{t_{v}}$) were considered as veritable extrema if their expression levels relative to the initial time point (i.e., *t*_0_ = 4 weeks) exceeded a given threshold (*E*^*th*^):

$$\begin{array}{c} if\;\;\;|E\left(\hat{t_{x}}\right)-E\left(t_{0}\right)|>E^{th}, \end{array}$$

$$\begin{array}{c} then\;\;\;t_{x}=\hat{t_{x}},\;\;\;x=p,v \end{array}$$

For profiles with both peaks and valleys, we determined the first peak or valley that satisfied this condition. We also classified monotonically decaying or increasing profiles as profiles without peak or valleys as defined above. Further, monotonicity required the additional condition that

$$\begin{array}{c} |E\left(\hat{t_{x}}\right)-E\left(t_{0}\right)|<E^{th0} \end{array}$$

for all *x* = (*p*, *v*). Monotonic increases versus decays were distinguished based on estimates of the profile’s first time derivative; *t*_*p*_ = *t*_0_ and *t*_*v*_ = *t*_0_ for monotonically decaying and increasing profiles, respectively. For visualization, scaled profiles were sorted and plotted according to peak or valley time. For this analysis, we set *E*^*th*^ = 0.5 and *E*^*th*0^ = 0.1.

### Differential network analysis

We evaluated the differences in the multi-organ gene-gene interaction networks between autonomic dysfunction versus control phenotypes. We describe our analyses based on conventional graph theoretic terminology, according to which the network is considered to be a graph *G* with vertices or nodes *V* that refer to genes which are connected by edges *E*: *G* = (*V*, *E*). The edges *E* are characterized by the interaction coefficients *k* described above. In particular, we addressed whether edges were added, removed, or switched in sign from autonomic dysfunction to control. We focused on the strongest network connections by considering edges for which the following condition was met:

$$\begin{array}{c} |E_{ij}|>med\left(E\right)+2sd\left(E\right) \end{array}$$

where *E*_*ij*_ is an edge from node *V*_*i*_ to node *V*_*j*_, *med*(.) is the median, and *sd*(.) is the standard deviation. Median values computed across all edges were exactly zero. Edges that did not meet our criteria were set to zero. All edge values were scaled to the interval (−1, 1) by applying *E*^*sc*^ = *E*/*max*(|*E*|). We evaluated differential network properties as follows. For each edge in *G*, we first computed the difference between unscaled edge values for control (WKY)- versus autonomic dysfunction (SHR)-specific networks as

$$\begin{array}{c} \Delta E_{ij}=E_{ij}^{SHR}-E_{ij}^{WKY} \end{array}$$

and we defined a threshold edge difference as follows:

$$\begin{array}{c} E^{th}=max\left(2sd\left(E^{WKY}\right),2sd\left(E^{SHR}\right)\right) \end{array}$$

Then we evaluated each edge for a set of conditions. Edges were considered to be added to the autonomic dysfunction network (absent for control but present for the autonomic dysfunction phenotype) if the following conditions were satisfied:

$$\begin{array}{c} |E_{ij}^{sc,SHR}|>0 \end{array}$$

$$\begin{array}{c} |E_{ij}^{sc,WKY}|=0 \end{array}$$

$$\begin{array}{c} |\Delta E_{ij}|>E^{th} \end{array}$$

Note that the third condition ensured that an edge could not be considered to be added to the SHR network if that edge was slightly larger than *E*^*th*^ for SHR but slightly smaller than *E*^*th*^ for WKY, such that the actual difference was negligible. Hence, only edge differences that exceeded *E*^*th*^ were considered. Edges were considered to be removed from the SHR network (absent for SHR but present for WKY) if the following conditions were satisfied:

$$\begin{array}{c} |E_{ij}^{sc,SHR}|=0 \end{array}$$

$$\begin{array}{c} |E_{ij}^{sc,WKY}|>0 \end{array}$$

$$\begin{array}{c} |\Delta E_{ij}|>E^{th} \end{array}$$

Finally, edges were considered to be switched in sign from WKY to SHR (e.g., *E*_*ij*_ = −1 for WKY and *E*_*ij*_ = 1 for SHR) if the following conditions were satisfied:

$$\begin{array}{c} |E_{ij}^{sc,SHR}|>0 \end{array}$$

$$\begin{array}{c} |E_{ij}^{sc,WKY}|>0 \end{array}$$

$$\begin{array}{c} sign\left(E_{ij}^{sc,SHR}\right)\neq sign\left(E_{ij}^{sc,WKY}\right) \end{array}$$

$$\begin{array}{c} |\Delta E_{ij}|>E^{th} \end{array}$$

We further filtered the data by analyzing count histograms of Δ*E*_*ij*_ values for added, removed, and switched edges and applying the following respective cutoffs to exclude edges with the smallest differences between SHR and WKY: 0.15, 0.15, and 0.2 (See S4 Fig). Networks characterized by edges that met the aforementioned criteria were visualized using *Cytoscape* [61].

### Bioinformatic analyses

We completed a number of analyses in which we utilized publicly available data and software tools, as described in detail below.

*Network motif analysis*: We used the *mfinder* tool to search for specific network motifs associated with cellular adaptation [62, 63]. We visualized the detected subnetworks using *Cytoscape* [61].

*Genetic analysis*: The genomes of numerous rat strains have been sequenced, including the strains used in our study: the Wistar Kyoto (WKY/NHsd) and the Spontaneously Hypertensive Rat (SHR/NHsd) [64]. For these strains, single nucleotide variants (SNVs) have been identified relative to the Brown Norway (BN) founder rat strain (genome build RGSC3.4) [64]. We downloaded the SNV data set in the VCF format from the Rat Genome Database during December 2015 (ftp://ftp.rgd.mcw.edu/) [65]. We isolated SNVs that were found in SHR/NHsd but not in WKY/NHsd. To test whether SHR-specific SNVs were present in the coding regions of the genes analyzed in this study, we downloaded the genomic coordinates for exons of interest from the UCSC genome table browser (rn4 rat genome, https://genome.ucsc.edu/) [66] and evaluated the presence of SNVs in these regions using *BEDTools* [67]. Similarly, to examine gene-proximal regulatory regions, we used the UCSC genome table browser to obtain the genomic coordinates of regions 2kb upstream of the transcription start sites (TSSs) [68, 69]. We identified SNVs in these regions using *BEDTools*. We further examined whether SNVs in TSS-proximal upstream regions, which are likely to contain regulatory elements [70], could potentially dysregulate gene expression by disrupting transcription factor (TF) binding. We considered regions ±60 bp relative to SNVs identified within 2kb of TSSs, and used the Genomatix software (https://www.genomatix.de/) to evaluate TF binding site (TFBS) enrichment [71, 72]. We identified putative TFBSs by computing Z-scores associated with the difference in counts of TF motif binding site nucleotide matches in the regions of interest as compared to the entire genome (see analysis details in [73]). We considered TFBSs to be statistically significant if Z > 2 was obtained, under the assumption that the Z scores were normally distributed and Z = 2 corresponds to *P* = 0.045. Visualizations of TFBS motifs were acquired from the Genomatix software interface.

## Results

### Time-series analysis of multi-organ gene expression reveals differential dynamics underlying disease progression

We characterized the pathogenesis of physiological dysregulation in autonomic dysfunction by examining organs anatomically integrated with the ANS. We temporally profiled the expression of genes implicated in ANS activity, peripheral/central INF, and RAS signaling (Fig 1A–1C). We acquired data over a span of animal ages encompassing the development of cardiovascular and metabolic disease. Arterial hypertension is a key feature of this animal model of autonomic dysfunction. We selected assay time points characterized by pre-hypertension (4, 6, and 8 wk), hypertension-onset (10 and 12 wk), and robust hypertension (16 wk) [40]. To determine whether specific samples or animals included in our study imparted systematic biases in our results, we utilized Principal Components Analysis (PCA). First, we reasoned that if specific samples or groups of samples were systematically biasing our results, such samples would appear to be distinct from the bulk of our samples in the subspace defined by the first two PCs. Such a finding was not obtained. Rather, all samples grouped together in a large aggregate (S1C Fig). To further examine whether specific animals selectively contributed to bias, we implemented PCA separately for each organ. We reasoned that if specific animals were biasing our results, such animals would be associated with pronounced variation across multiple organs. Our analysis showed that animals with pronounced gene expression differences in one organ were often similar to the majority of the other animals when considering the other organs (S1D and S1E Fig). These analyses did not suggest any systematic sample outliers, and hence, all samples analyzed using qPCR were included in our downstream computational analysis. Our analysis framework consisted of statistical approaches, dynamics analysis, network modeling and analysis, and bioinformatics (Fig 1D–1F).

**Fig 2:**
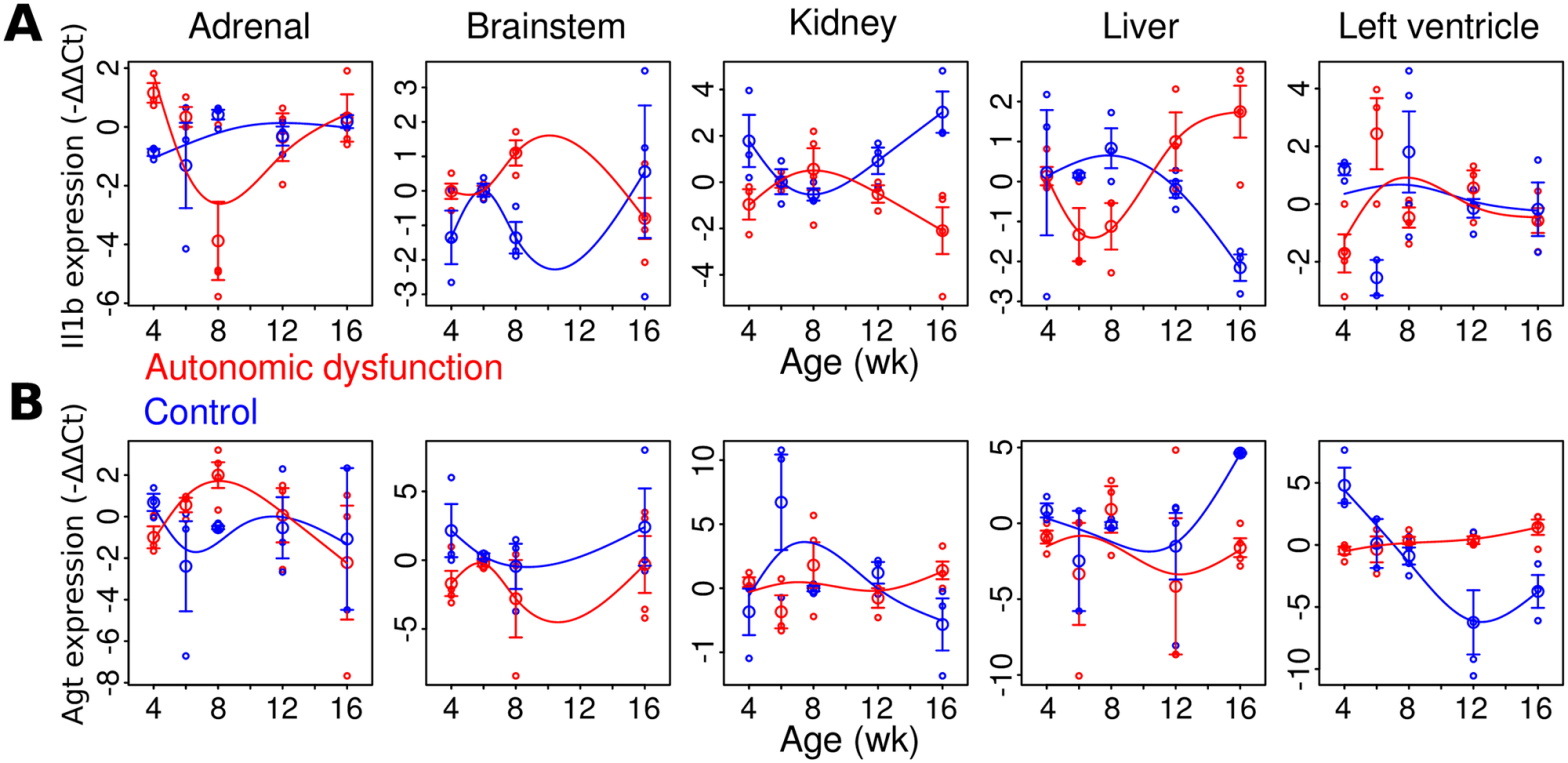
Divergent patterns of dynamic gene expression across organs and genes correspond to autonomic dysfunction. (A) Expression dynamics of pro-inflammatory gene *Il1b* across organs and phenotypes. (B) Expression dynamics of angiotensin precursor gene *Agt*. Error bars indicate standard error of the mean. Smooth curves depict natural cubic splines fit to the data.

To examine whether gene expression dynamics were distinct in autonomic dysfunction, as compared to the control phenotype, we performed statistical analyses of gene expression dynamics. We implemented the *Optimal Discovery Procedure* [11] to address whether the temporal profiles for a given gene in a given organ differed significantly between autonomic dysfunction and control phenotypes. In this analysis, temporal profiles of gene expression were characterized by cubic splines fitted to the data (Fig 2). The time series analysis identified numerous statistically significant differences in the temporal dynamics of gene expression (Fig 2, S5 Fig). In particular, all temporal expression profiles from the brainstem showed significant differences in autonomic dysfunction (false discovery rate <0.1, S5 Fig). Overall, these results highlight the extensive differences in dynamics of gene expression between disease and control phenotypes. Note the apparent gene expression differences observed at the 4 wk time point; we address this finding in the discussion. Importantly, at time points in which the disease and non-disease phenotypes exhibited similar gene expression levels, discrepancies in the underlying dynamic trajectories indicated divergent properties of the underlying gene regulatory networks.

### Robustness analysis of the HMF method

We thoroughly evaluated the robustness of the *HMF method* for identifying network models of multi-organ gene regulatory networks. We have previously shown that the *HMF method* achieves robust performance in the identification of gene regulatory network dynamics and structure [48]. We further evaluated the robustness of our network identification results with respect to regression regularization parameters *λ* and *α*, structural properties of the identified networks, and sensitivity to exclusion of data subsets.

We performed the following analyses to compare identified networks with the ‘best fit’ network characterized by *J*_*sim*_ = *min*(*J*_*sim*_):

1. Spearman rank correlation of interaction coefficient values
2. Fisher’s exact test for counts of coefficient signs (±1)

For each phenotype, we compared the ‘best fit’ network with many subnetworks. For these comparisons, we evaluated Spearman rank correlation coefficients, Fisher’s exact test (FET) p-values, and odds ratios. For the autonomic dysfunction phenotype, a total of 272 comparison networks were identified and we obtained Spearman rank correlation ≥ 0.77 (*P* < 2.2 × 10^−16^, S6A Fig left) along with FET *P* ≤ 2.1 × 10^−35^. All odds ratios approached infinity. For the control phenotype, 289 networks met our criteria for comparison, Spearman rank correlations exceeded 0.79 (*P* < 2.2 × 10^−16^, S6A Fig right), and the FET indicated a high degree of interaction coefficient sign similarity (*P* ≤ 1.0 × 10^−22^). The corresponding odds ratios approached infinity due to the absence of coefficient sign discrepancies. In general, for both autonomic dysfunction and control, with *α* > 0 there was a negligible influence of *λ* on network correlations for *λ* ∼ *λ*_*min*_ (S6B Fig). While the control network correlations were generally insensitive to the *α* value, the autonomic dysfunction networks showed a small degree of sensitivity to *α*. Finally, the range of *m* values exerted a negligible influence on network correlations. This is illustrated by the aggregates of data points, of each *λ*/*α* combination, corresponding to the ten *m* value ranges assessed (S6B Fig).

We further examined the robustness of out network identification procedure, with respect to regularization parameters *λ* and *α*, by determining three key features of network topology [74]:

1. Average path length: < ℓ >
2. Clustering coefficient: *C*_*i*_
3. Degree distribution: *γ*

In general, our network analyses revealed that autonomic dysfunction and control networks were characterized by similar topological properties that were largely consistent with those of other biological networks. We observed < ℓ > ∼ 1.5 − 3 (S7A Fig), well within the typical range for biological networks [75, 76]. Similarly, the clustering coefficient values *C*_*i*_ ∼ 0.1 − 0.4 were consistent with previous observations (S7B Fig) [76, 77]. For our analysis of the power law exponent, we considered only fits for which a K-S p-value of *P* > 0.8 was obtained, as this was consistent with reasonable evidence supporting the power law degree distribution (S7C Fig). The exponent values generally fell in the range of *γ* ∼ 2−8. Exponent values of 2 < *γ* < 3 are typically observed for biological networks [60], in partial agreement with our findings. The discrepancy, however, is not surprising given that our analysis included a limited number of functionally related genes, while global networks on the genomic scale would be expected to have distinguishable properties. Further, the elevated values of *γ* we observed are consistent with the small-world topology associated with the power law distribution. In general, our correlation-based and graph theoretic analyses support the robustness of our system identification approach with respect to reliability of edges identified and global network properties.

While the findings so far support the robustness of our approach to variation in regression parameterization, we wanted to further address whether we could identify similar edges if subsets of the full network were considered. We considered subsets of both organs and genes. Eight organ subsets were considered:

1. Adrenal gland, Brainstem
2. Brainstem, Kidney
3. Brainstem, Left ventricle
4. Kidney, Left ventricle
5. Brainstem, Kidney, Left ventricle
6. Adrenal gland, Kidney, Left ventricle
7. Adrenal gland, Brainstem, Kidney, Left ventricle
8. Adrenal gland, Brainstem, Kidney, Liver, Left ventricle

For each organ subset, we considered four combinations of gene annotations:

1. Inflammation, Autonomic
2. Inflammation, RAS
3. Autonomic, RAS
4. Inflammation, Autonomic, RAS

**Fig 3:**
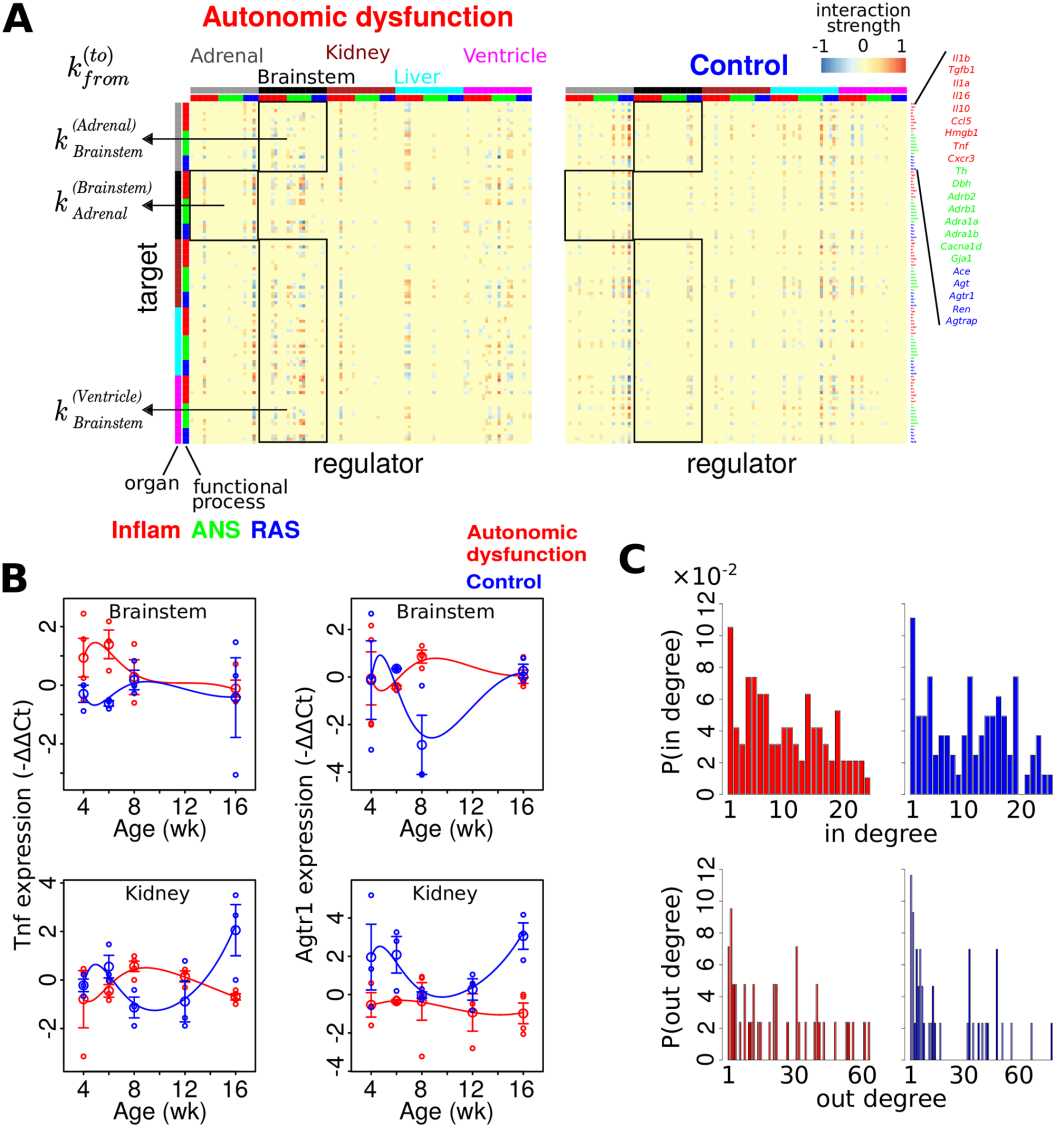
Data-driven model-based network identification shows that connectivity differences are associated with dynamic differences in autonomic dysfunction. (A) Phenotype-specific network topology representations of gene-gene interaction coefficients. A given coordinate represents the magnitude of influence of the organ-gene in the corresponding column on the organ-gene in the corresponding row (see Fig 1E). (B) Sample dynamic profiles obtained from the simulation of the mathematical models associated with the interactions described in (A). (C) Degree distributions for both in and out degree. These data show that differences in network topology are associated with differential dynamics in autonomic dysfunction versus control.

Thus, we evaluated 32 separate iterations of our network identification approach, with distinct network subsets, for both autonomic dysfunction and control phenotypes. We provide compact examples of identified network structures via parameter heatmap representations (Fig 3A) and corresponding connectivity illustrations (S8 Fig). We considered the coefficient data for the best fit over ten *λ* values and ten *m*-value ranges. As described above, we compared each sub-network to the full network based on the Spearman rank correlation and FET, including normalized interaction coefficients *k* ∈ (−1, 1) that exceeded two standard deviations from the median in both the full network and the comparison network. We also required that at least five coefficient values meet the stated criteria for both networks. For the control network comparisons, our comparison criteria were satisfied in all of the comparisons. Our results showed Spearman rank correlation ≥ 0.77 (*P* ≤ 3.8 × 10^−4^), FET *P* ≤ 8.1 × 10^−5^, and all odds ratios approached infinity. For the autonomic dysfunction phenotype, all sub- networks satisfied our comparison criteria, Spearman rank correlation ≥ 0.71 (*P* ≤ 4.2 × 10^−5^), FET *P* ≤ 7.4 × 10^−7^, and all odds ratios approached infinity.

In considering our correlation-based and graph theoretic analyses across regularization conditions, along with our correlational analyses of subnetwork comparisons, we concluded that our approach is robust to regression parameterization and the inclusion/exclusion of dynamic variables.

### System identification yields dynamic model-based phenotype specific regulatory networks

To examine the dysregulation of physiological homeostasis, we applied systems identification to infer network models corresponding to the autonomic dysfunction and control phenotypes. We utilized the *HMF method*, which entailed the application of the *Hartley transform* and associated *Hartley modulating functions*, to infer data-driven dynamic network models. The principal advantages of the *HMF method*, in comparison to related methods, are that (1) this approach facilitates the identification of continuous-time mathematical models of gene expression from discrete data, and (2) this approach obviates the need to compute temporal derivatives of discrete data by instead utilizing a frequency domain transformation of the data [48].

The network dynamics were represented by *ordinary differential equations* (ODEs) that described continuous models of gene expression. The network structures were represented by interaction coefficients that describe either gene regulatory interactions in which one gene’s activity promotes the activity of another gene (‘upregulation’), or interactions in which one gene inhibits the activity of another gene (‘downregulation’, Fig 1E). We consider the terms upregulation and downregulation to represent indirect influences in most cases, rather than direct mechanistic influences.

The network models identified for autonomic dysfunction and control phenotypes depicted the topology of gene-gene influences within and across organs (Fig 3A, S8 Fig). It is noteworthy that regulatory interactions within the brainstem were relatively absent in the control phenotype as compared to autonomic dysfunction. Moreover, brainstem genes showed extensive cross-organ influence patterns in autonomic dysfunction, whereas these interactions were comparatively under-represented in the control (S9 and S10 Figs). It is also worth noting that because we did not have 12 wk data for the brainstem samples the identified brainstem networks were more prominently constrained by the earlier time points; we return to this potential limitation in the discussion.

The gene-gene influences underlying the multi-organ network were determined by using our time-series data to computationally infer the interaction coefficients of a mathematical model formulated by ODEs. According to this approach, the *HMF method* was utilized to infer phenotype-specific regulatory interactions and expression dynamics. Our simulated dynamic models showed considerable agreement with the experimental data (Fig 3B; see S3 File for all time series data along with dynamic model simulations). We refer to each simulated timeseries as an “expression profile” (smooth traces in Fig 3B). Code and annotation information for implementing our models in Matlab, R, and Systems Biology Markup Language are available (S4, S5, S6, S7, S8, S9 and S10 Files).

To further characterize the properties of phenotype-specific networks, we evaluated the respective degree distributions [74]. The *in degree* of *gene x* indicates the number of genes that influence *gene x* (i.e., the number of non-zero values of a given row in Fig 3A). The *out degree* of *gene x* denotes the number of genes that *gene x* influences (the number of non-zero values in the column corresponding to *gene x* in Fig 3A). Our analysis showed that the autonomic dysfunction network exhibits a similar though distinct degree distribution, as compared to the control network (Fig 3C). Overall, the gene-gene influence matrices (Fig 3A) along with the degree distribution analysis (Fig 3C) showed that differential network topology is associated with dynamic disturbances in autonomic dysfunction that were identified by timeseries analysis (Fig 2, S5 Fig).

### Aberrant gene expression cascades underlie the pathogenesis of autonomic dysfunction

**Fig 4:**
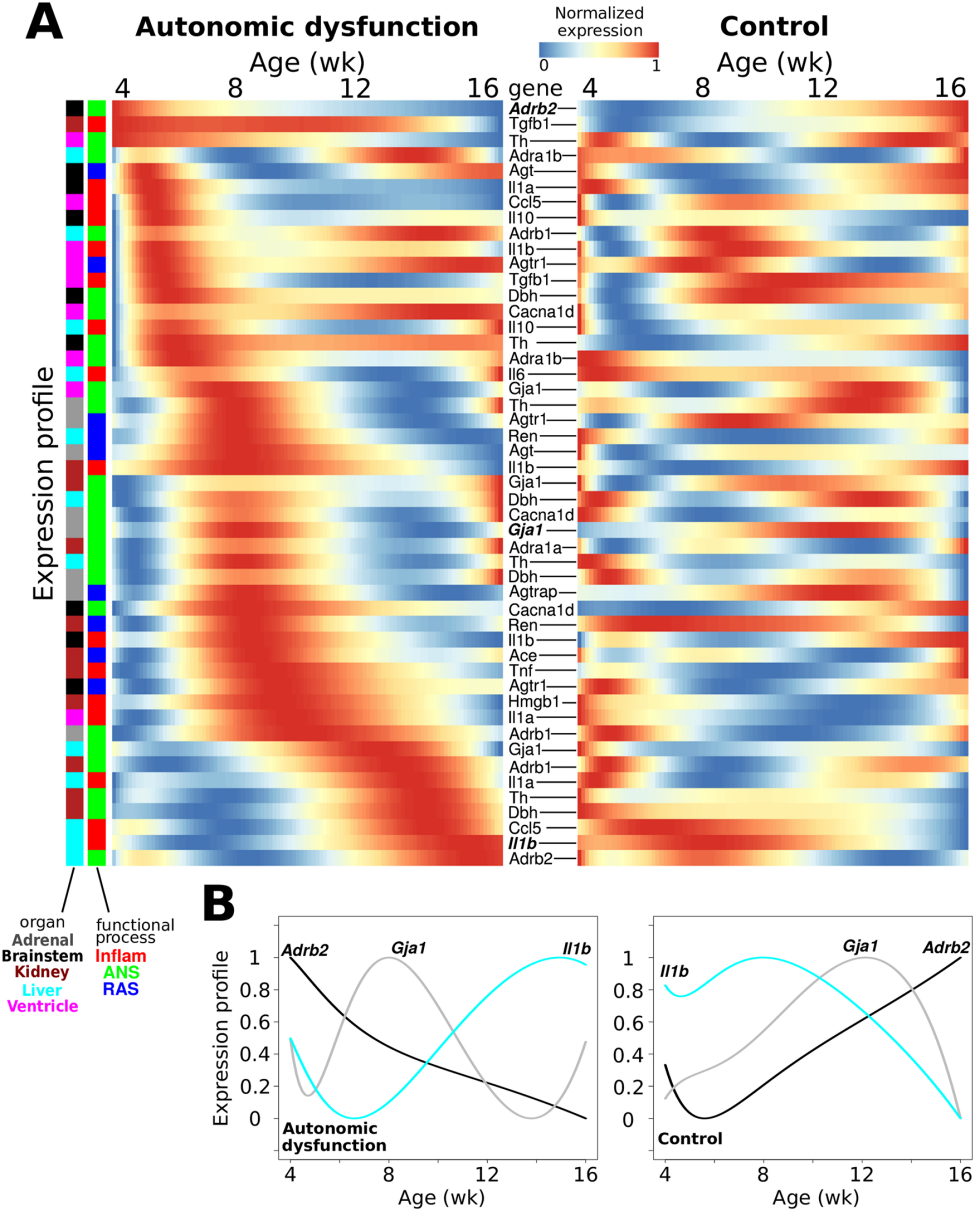
Autonomic dysfunction is associated with a disease-specific regulatory cascade of gene expression patterns. (A) Gene expression profiles from the autonomic dysfunction phenotype were sorted according to the time that the expression profile showed a peak. The analysis shows a cascade or peak times associated with autonomic pathogenesis and corresponding hypertension development (left). Comparison with control profiles (right) show that the autonomic dysfunction cascade is disease-specific. (B) Examples of temporal profiles with relatively early, intermediate, and late peaks corresponding to the autonomic dysfunction (left) and control (right) phenotypes.

Developmental processes are often characterized by coordinated waves of precisely timed gene expression patterns [13, 78]. We hypothesized that an analogous coordinated cascade of dynamic transcriptional patterns regulates disease progression. We investigated whether temporal sequencing of gene expression corresponded to the development of autonomic dysfunction. We analyzed the sequence of peak times observed in the temporal profiles for the autonomic dysfunction and control phenotypes (Fig 4). In this analysis, we considered genes for which prominent expression peaks were not preceded by prominent expression valleys. Profiles with monotonically decreasing expression levels were considered to exhibit peaks at the initial time point of four weeks [13]. Several brainstem profiles showed peak activity at early pre-hypertensive ages including *Adrb2*, *Agt*, *Il1a*, and *Il10* (Fig 4A). Similarly, early peaks were observed for kidney *Tgfb1*, liver *Adra1b*, and ventricle *Th* and *Ccl5* (Fig 4A). By comparison with the control expression profiles, our analysis revealed a coordinated cascade of gene expression activation patterns that was specific to the autonomic dysfunction phenotype (Fig 4B).

We expanded our analysis by examining gene expression profiles with valleys in autonomic dysfunction (S11 Fig). Similar to the results for the peak analysis, we found that disease development was associated with a cascade of valleys that was specific to the autonomic dysfunction phenotype. Moreover, when we completed peak and valley analyses and ordered the expression profiles according to the control phenotype, we found that distinct dynamic patterns were control phenotype-specific (S12 and S13 Figs). Our dynamic pattern analyses showed that distinct sets of temporal profiles exhibit early peaks/valleys, and the respective peak/valley times were associated with various temporal offsets relative to disease onset. To summarize these findings, we analyzed the peak and valley times for autonomic dysfunction versus control (S14 Fig). This analysis highlighted genes that showed similar versus divergent dynamic patterns of peak/valley timing with respect to phenotype (S14 Fig). Our findings indicate that different subsets of the multi-organ network exhibit distinct aberrations in gene expression dynamics, thereby indicating that gene regulatory networks are re-wired under disease conditions.

### Differential gene regulatory network wiring is associated with autonomic dysfunction

Given our finding of kinetic and network structure differences corresponding to the development of autonomic dysfunction (Figs 3 and 4), we wanted to further examine the specific molecular underpinnings of these differences. Divergent molecular networks have been associated with cardiovascular disease and aberrant ANS function [40, 79]. Such connectivity differences reflect re-wiring of the molecular networks that determine the gene regulatory interaction dynamics which underly cell, tissue, and organismal physiology [80]. To interrogate the molecular underpinnings of disease-relevant multi-organ network modifications, we examined the network degree distribution, differential network module structure, and specific gene regulatory influences that were re-wired in the disease phenotype [79, 81, 82].

**Fig 5:**
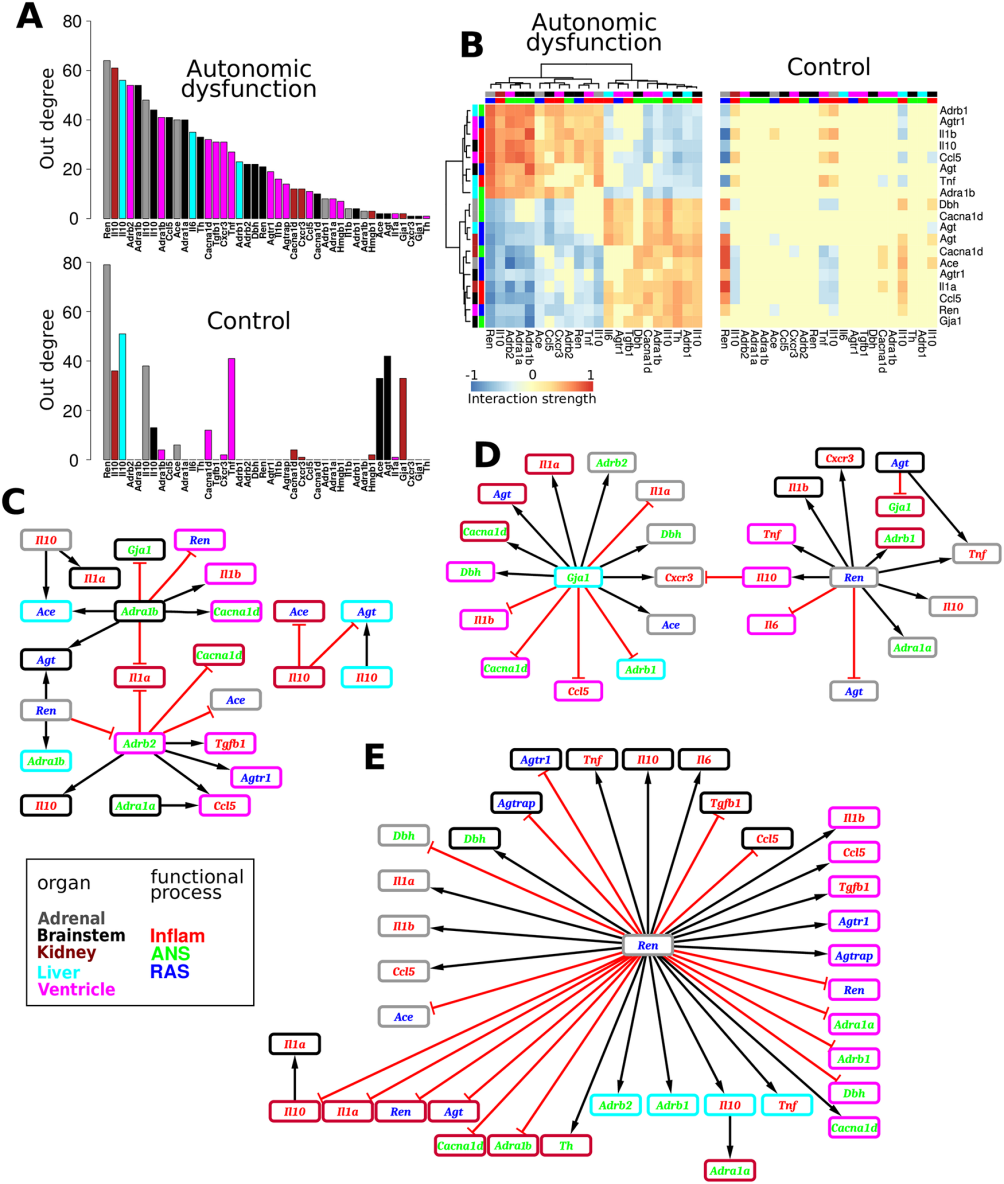
Differential network structure in autonomic dysfunction. (A) Out degree is shown according to the degree order of the autonomic dysfunction network. The control network exhibited a divergent out degree pattern. (B) A highly connected module in the autonomic dysfunction network with genes that have prominent influences (columns, regulators) and their targets (rows). Each row and column of the full network matrix (Fig 3A) contains more than 15% of non-zero entries. The corresponding control subnetwork is comparatively sparse. (C) Diagram illustrating gene-gene interactions that are present only in the autonomic dysfunction network. (D) Interactions that are only present in the control network. (E) Autonomic dysfunction-specific interactions that have the opposite sign of the corresponding control interactions (e.g., adrenal *Ren* upregulates brainstem *Tnf* in the autonomic dysfunction phenotype but downregulates brainstem *Tnf* in the control phenotype).

**Fig 6:**
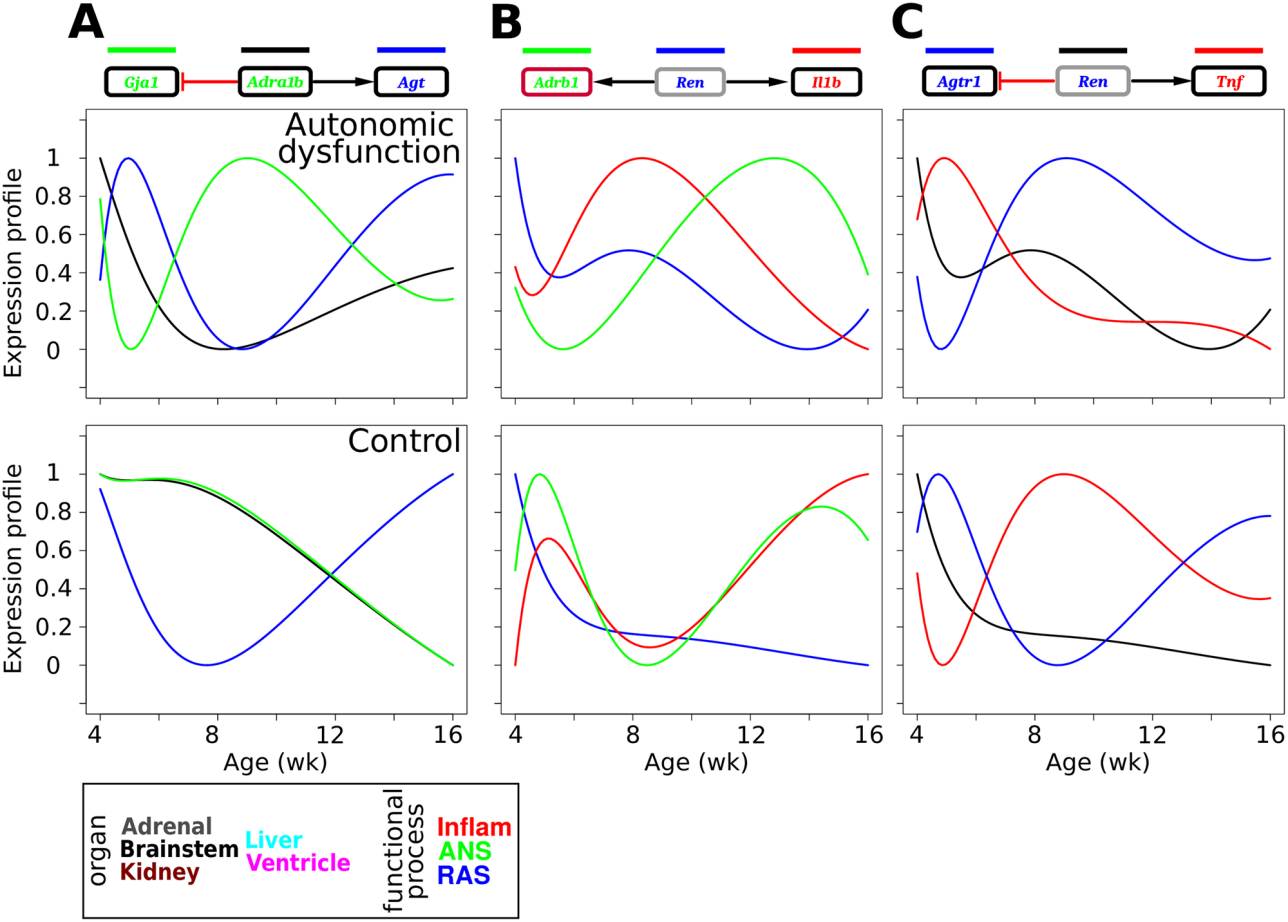
Divergent dynamics corresponding to differential network structure in autonomic disease. (A) Dynamic profiles related to gene-gene interactions exclusive to the autonomic dysfunction network. (B) Dynamic profiles related interactions exclusive to the control network. (C) Dynamic profiles related inverted interactions in the autonomic dysfunction phenotype as compared to the control.

**Fig 7:**
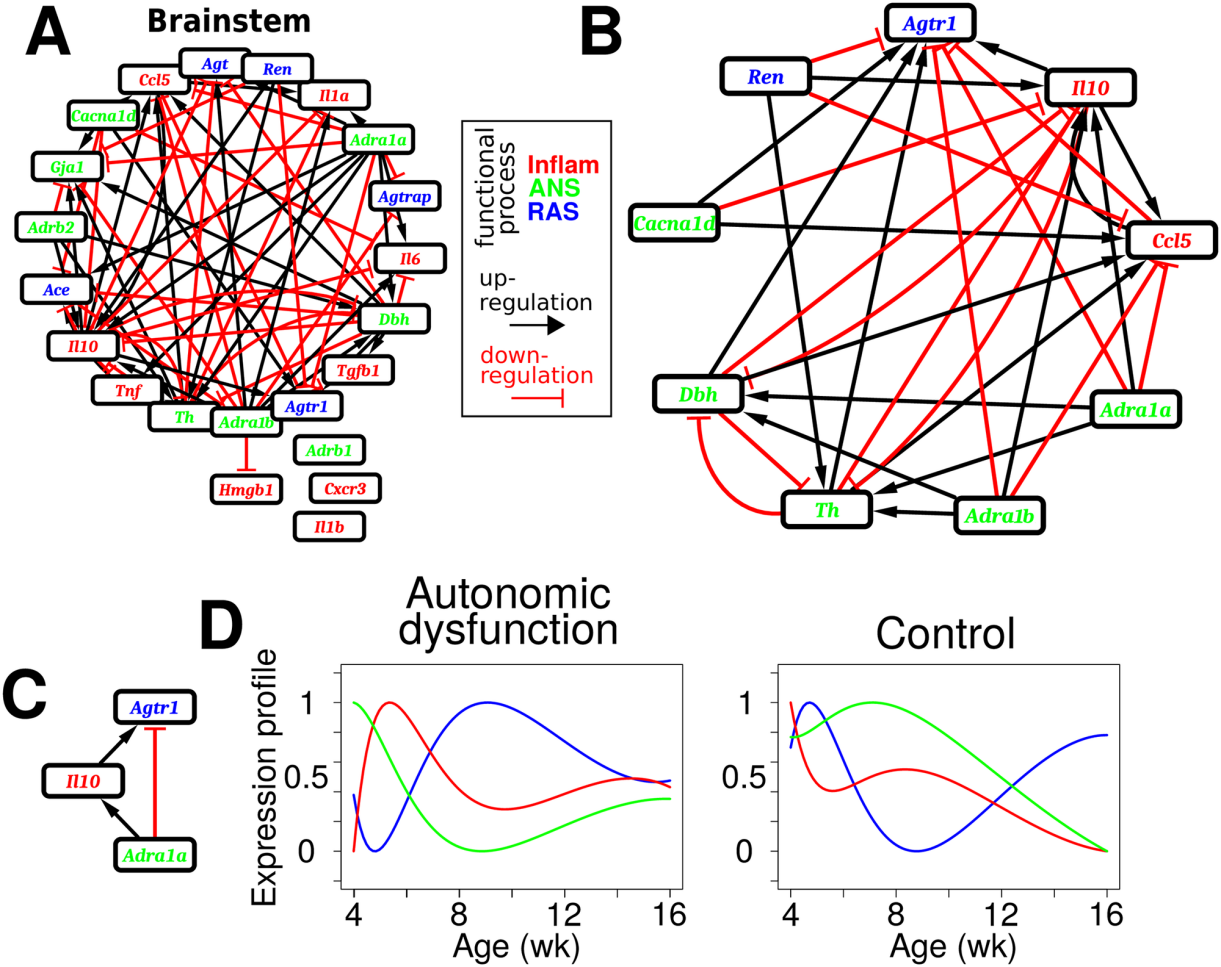
Dynamic profiles support specific network interactions in the brainstem of the autonomic dysfunction phenotype. (A) Brainstem subnetwork for the autonomic dysfunction phenotype. (B) Interactions involving *Agtr1* are highlighted. (C) Example of a three node ‘adaptation motif’ from the autonomic dysfunction phenotype. (D) Dynamic profiles corresponding to the motif from (C) show expected behavior only for the autonomic dysfunction phenotype.

We analyzed the disease-specific *out degree* distribution and examined the ordering of *out degree* across genes (Fig 5A). Genes with the highest *out degrees* included adrenal *Ren* and brainstem *Il10*. Interestingly, adrenal *Ren* showed an pre-hypertensive valley in autonomic dysfunction (Fig 6B) and brainstem *Il10* showed a pre-hypertensive peak (Fig 7D), whereas differential dynamics were observed for the control phenotype. These results suggest the possibility that changes in adrenal *Ren* and brainstem *Il10* are key early events that critically influence the pathogenesis of autonomic dysfunction by virtue of their respective kinetics and substantial participation in the regulation of gene expression within and across organs.

Modules of highly interconnected subnetworks or modules of gene regulatory interactions are critical for the regulation of biological networks [82]. We tested whether we could identify a highly connected module in the autonomic dysfunction network that would be expected to critically regulate autonomic (dys)function. We detected such a module (Fig 5B) which highlighted some of the key influences of genes with high *out degrees* (Fig 5A). For instance, adrenal *Ren* upregulated the expression of several genes including liver *Adrb1*, ventricular *Agtr1*, and brainstem *Il10*. In contrast, adrenal *Ren* downregulated the expression of these genes in the control phenotype (Fig 5B, right). Moreover, adrenal *Ren* downregulated genes including brainstem *Agtr1* in autonomic dysfunction but exerted a corresponding upregulatory influence in the control network (Fig 5B).

To further define the network differences between the autonomic dysfunction and control phenotypes, we queried the networks for gene-gene interactions that were present on only one network as well as interactions that exert opposing influences with respect to phenotype (e.g., interactions that are upregulating in autonomic dysfunction but downregulating in the control network) [83]. We applied a conservative approach that identified only the interactions that showed relatively large differences, yielding a high confidence set of network differences (Methods, “Differential network analysis”). Our analysis revealed that a number of gene regulatory interactions were present exclusively in the autonomic dysfunction network (Fig 5C). For instance, brainstem *Adra1b* expression dynamics influenced the temporal patterns of *Agt* and *Gja1* expression in the brainstem for the autonomic dysfunction phenotype. To determine if the observed gene regulatory network differences in autonomic dysfunction could be related to differential dynamics of gene expression, we examined the phenotype-specific brainstem expression profiles. This analysis showed that, for the autonomic dysfunction phenotype, *Adra1b* exhibited a valley following and early peak in *Agt* and an early valley in *Gja1* expression (Fig 6A). Further analysis showed that the decrease in *Adra1b* preceded decrease in *Agt* and increase in *Gja1*. Considered together, these temporal profiles from the autonomic dysfunction phenotype were consistent with the topological interactions amongst these brainstem genes based on the identified network.

We next examined whether there was evidence for control–specific network interactions (Fig 5D). Our analysis revealed that control adrenal *Ren* upregulated brainstem *Il1b* and kidney *Adrb1* expression. We studied the respective temporal profiles and found that a decrease in adrenal *Ren* preceded a decrease in brainstem *Il1b* and kidney *Adrb1* in the control phenotype, presumably through reduction of an upregulating influence (Fig 6B). In contrast, for the autonomic dysfunction phenotype, brainstem *Il1b* and kidney *Adrb1* dynamics showed increases during the pre-hypertensive and hypertension-onset time frames, respectively, which could be related to the absence of *Ren*-mediated influence (Fig 6B).

We concluded our differential network analysis with an evaluation of whether we could identify gene-gene influences that switched sign (e.g., from positive upregulation to negative downregulation) in the autonomic dysfunction phenotype relative to the control network. Our analysis showed evidence for inverted interactions, the majority of which involved adrenal *Ren* (Fig 5E). For example, adrenal *Ren* upregulated *Tnf* but downregulated *Agtr1* in the brainstem of the autonomic dysfunction phenotype. The respective expression profiles showed that the attenuation of adrenal *Ren* expression in autonomic dysfunction preceded a decrease in brainstem *Tnf* and an increase in brainstem *Agtr1*. These results could be due to attenuated upregulation and downregulation influences of adrenal *Ren* on brainstem *Tnf* and *Agtr1*, respectively (Fig 6C). Consistent with the inversion of these interactions, the temporal patterns of brainstem *Tnf* and *Agtr1* expression was inverted in the control phenotype relative to that of autonomic dysfunction (Fig 6C). In sum, our studies on differential dynamics and network structure illustrate that our integrated analyses can be applied to identify potential disease mechanisms associated with the organ-specificity and timing of gene expression.

### Network motif analysis-based identification of a core module involving the brainstem angiotensin-II receptor transcript

Because our differential network analysis was designed to impose strict criteria for the attribution of phenotypic differences in gene-gene interactions, we elaborated on this analysis. Given the phenotypic disparity in intra-organ gene-gene connectivity in the brainstem (Fig 3A), we specifically focused on the brainstem subnetwork. Further, because connectivity was largely absent in the control brainstem network (S15 Fig), we focused exclusively on the autonomic dysfunction brainstem network (Fig 7A). In particular, we posed the question of whether we could identify network motifs associated with the adaptation to prolonged stimuli [84]. Network motifs that support adaptation have important biological functions and the motif dynamics are readily interpret-able [84, 85]. Hence, we tested whether we could identify three node motifs capable of adaptation in the brainstem network of the autonomic dysfunction phenotype (Fig 7A).

Our analysis of the autonomic dysfunction brainstem subnetwork showed numerous three node feed-forward motifs, many of which could support adaptation according to topological considerations [84] (S16 Fig). Brainstem expression of the angiotensin-II type-1 receptor, encoded by *Agtr1*, is known to exert prominent influences on ANS function. Hence, we focused on motifs including *Agtr1* (Fig 7B). We examined the expression profiles of 3-node motifs including *Agtr1*. This analysis showed that it was difficult to interpret many of the motifs in terms of the corresponding dynamic expression profiles. For instance, consider a motif in which *Dbh* upregulates both *Agtr1* and *Ccl5*, while *Ccl5* downregulates *Agtr1* (S17A Fig). The similarity between the temporal profiles of *Ccl5* and *Agtr1* is inconsistent with the downregulation of *Agtr1* by *Ccl5* (S17B Fig, left).

Many other motifs were associated with similar ambiguities regarding interpretation of the dynamics. In contrast, a motif involving *Adra1a*, *Il10*, and *Agtr1* was associated with temporal dynamics that agreed well with predictions based on motif topology (Fig 7C). Elevated levels of *Adra1a* were followed by increased levels of *Il10* and decreased levels of *Agtra1* (Fig 7D, left), consistent with the respective upregulating and downregulating influences of *Adra1a* on *Il10* and *Agtr1*, as defined by the motif topology (Fig 7C). As *Adra1a* levels were decreased with time, a subsequent reduction of *Il10* and increase of *Agtr1* was observed, consistent with the motif structure (Fig 7C and 7D). Despite the existence of numerous feedback loops and complex arrangements of interactions, our analyses revealed that structural features of our multi-organ model could be related to the underlying dynamics in a context-dependent manner. Taken together with our differential network analysis findings, our results suggest that there are core modules that exhibit dynamic behavior consistent with isolated subsets of gene-gene interactions both within and across organs.

### Bioinformatic identification of regulatory elements modified by genetic variants associated with autonomic dysfunction

Disease-specific genetic aberrations can contribute to, rather than respond to, the disease state [86]. We tested whether autonomic dysfunction was associated with disease-specific single nucleotide variations (SNVs) that could be related to either protein sequence or regulatory regions of DNA that function as putative transcription factor binding sites (TFBSs). Mutations in TFBSs have been shown to be implicated in complex disease pathology [87], and identification of novel regulatory SNVs could yield important insight as to the mechanistic basis of the alterations of network dynamics and connectivity we observed in autonomic dysfunction.

We initially focused on examining the presence of SNVs in coding regions of genes for which expression was analyzed in our study. We identified several coding SNVs that were all synonymous, such that the corresponding amino acid sequence remains unchanged by the SNV. These SNVs have been previously annotated (rs8154045, rs10546839, rs198233992, rs199262884, rs197275571, rs198523377, rs8174203) [88]. While it is possible that synonymous SNVs could alter gene expression, the functional implications of such variants are currently unclear [89].

**Fig 8:**
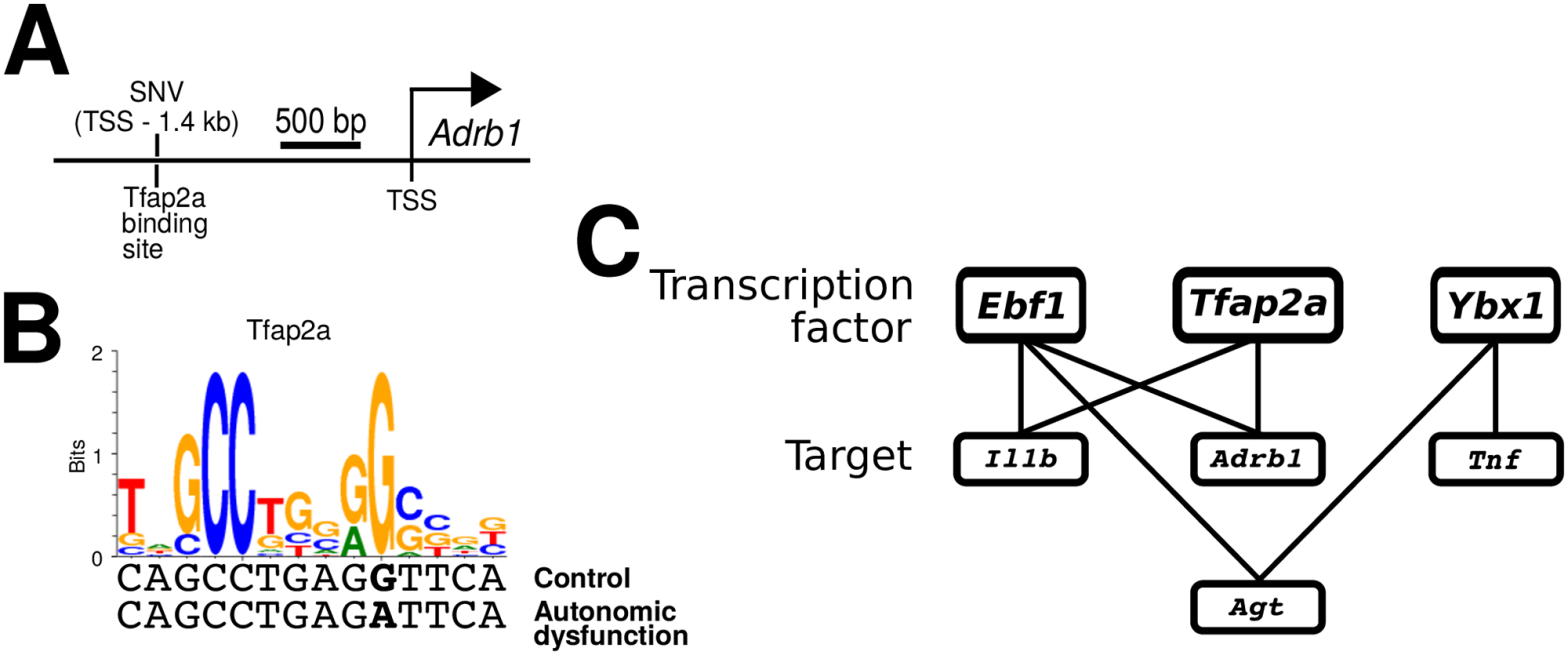
Autonomic dysfunction-specific single nucleotide variants in regulatory regions upstream of prominent genes. (A) Example spatial relations amongst an SNV, TFBS, and transcription start site. (B) Transcription factor binding motif associated with *Tfap2a* along with the respective sequences in the autonomic dysfunction and control phenotypes. A single nucleotide variation (SNV) occurs in a key element of the putative binding site for the autonomic dysfunction phenotype. (C) Summary of TFs with bioinformatically identified TFBSs in or near autonomic dysfunction-specific SNVs upstream and proximal to genes implicated in physiological homeostasis.

Next, we determined whether 2 kb regions upstream of the transcription start sites for our genes of interest contain SNVs in the autonomic dysfunction phenotype. The analysis revealed several SNVs in these gene-proximal upstream regions, which are putative regulatory sites associated with TF binding. Recent analyses have shown that SNVs either within or in proximity to TFBSs can modulate transcriptional regulation [90], with relevance to disease phenotypes [91]. To examine whether the SNVs we identified could potentially disrupt TF binding in gene-proximal regulatory regions, we evaluated the statistical enrichment of TFBSs in the vicinity of the SNVs within these regions (SNV ±60 bp [71]). Our analysis revealed four SNVs with statistically significant enrichment for TFBSs. We found that the upstream region of *Adrb1* contains a autonomic dysfunction-specific SNV within a putative binding site for Tfap2a (Fig 8A). Similarly, we found other TFBS enriched sequences near gene-proximal SNVs in the autonomic dysfunction phenotype (Fig 8C, S18 Fig). In addition to Tfap2a, the analysis identified TFBSs for Ebf1 and Ybx1, with sites proximal to SNVs associated with *Adrb1*, *Agt*, *Il1b*, and *Tnf* in the autonomic dysfunction phenotype (Fig 8C, S18 Fig). Our analyses suggest that altered connectivity and dynamics in autonomic dysfunction could be related to genetic variations that influence the expression of functionally important genes.

## Discussion

Chronic diseases evolve over time scales from weeks to years, and involve multiple physiological systems. Thus, co-morbidities involving impairments of multiple organs are common in complex chronic conditions such as cardiovascular disease [1, 2]. In this study we focused on a disease phenotype associated with autonomic dysfunction and co-morbidities including hypertension, metabolic disorder, and cognitive impairment. Neurogenic hypertension has also been associated with numerous disease conditions including sleep apnea, renal failure, and heart disease [92]. We implemented a unique and novel synthesis of experimental and computational approaches involving molecular biology, time series analysis, system identification, dynamic network analysis, and bioinformatics. While the identification and analysis of gene regulatory networks is common, such studies typically do not include the identification of continuous-time models in which the network dynamics and structure are jointly described by a single mathematical formalism [93]. Importantly, while previous studies have examined multi-organ network dynamics at acute time scales [20, 23, 94], we focused on the pathogenesis of a chronic disease state. Our investigation yielded convergent lines of evidence suggesting that aberrant brainstem function is a key initiating process in the pathogenesis of autonomic dysfunction.

Our analyses showed that a disease-specific cascade of molecular activation patterns is associated with dysregulated multi-organ network connectivity. Initial transients in the molecular expression cascades were observed for brainstem genes. Further, the brainstem network associated with autonomic dysfunction exhibited a high degree of connectivity in comparison to the control brainstem network. The disease-specific network included autonomic, inflammatory, and RAS genes. These results are consistent with the hypothesis of neurogenic hypertension, which predicts that augmented sympathetic drive promotes hypertension through mutual interactions amongst the autonomic, immune, and RA systems [24]. Data aligned with this hypothesis showed that hypertension is associated with the augmentation of catecholaminergic signaling, neuroinflammation, and RAS signaling in the brainstem [95, 96]. Our results provide an integrative network based framework for interpreting these previous observations. Our analyses revealed putative network-level influences spanning a set of organs that collectively maintain physiological homeostasis.

Our network analyses revealed a prominent dysregulation of renin expression dynamics in the adrenal gland, and dysregulation of multi-organ interactions involving adrenal renin in the autonomic dysfunction phenotype. Renin showed an expression decrease that preceded the onset of hypertension in the autonomic dysfunction phenotype. This result suggests the possibility that the disinhibition of adrenal renin targets, coincident with the decrease in renin expression, is implicated in the pathogenesis of autonomic dysfunction. However, as noted below, our current study did not conclusively establish whether specific molecular events were strictly causal for subsequent regulation of molecular expression or physiological function. Nevertheless, our hitherto unobserved findings suggest that local RAS signaling in the adrenal gland may elicit systemic endocrine signaling to regulate gene expression in multiple organs. For instance, our network analysis showed that adrenal renin downregulated the brainstem type-1 angiotensin receptor (AT1R) expression the autonomic dysfunction phenotype, but upregulated AT1R expression in the control phenotype. Combined with the data showing a decrease of adrenal renin expression during the development of autonomic dysfunction, our analyses suggest that adrenal renin downregulation may drive the onset of autonomic dysfunction through the disinhibition of AT1R expression in the brainstem.

We identified that specific brainstem network motifs involving the transcript encoding AT1R, an important target of antihypertensive therapeutics [97, 98]. Our analyses suggested that AT1R is prominently influenced the *α*1 adrenergic receptor and IL-10 in the hypertensive brainstem. Both the *α*1 adrenergic receptor and IL-10 have been previously implicated in autonomic influences on blood pressure control [99, 100]. Our results suggest that, even though AT1R could be regulated in a complex manner by a number of regulatory influences, *α*1 adrenergic receptor and IL-10 may regulate sympathetic outflow through prominent influences on AT1R expression. The examination of the intracellular pathways coupling *α*1 adrenergic receptor and IL-10 to AT1R expression could improve our understanding of the molecular and cellular mechanisms of neurogenic hypertension, and could elucidate novel approaches for therapeutic treatments based on an understanding of network topology and molecular expression dynamics. For example, network-based pharmacological interventions directed at the regulation of AT1R prior to hypertension could prevent or slow disease progression [101].

We utilized available sequencing data from a number of rat strains [64] and performed a bioinformatic analysis to determine putative regulatory nucleotide variations between the disease and control rat strains utilized in our experiments. Our analysis identified several novel disease-specific nucleotide variants in promoter-proximal regions upstream of genes implicated in autonomic, inflammatory, and RAS functions. We found that genes encoding angotensinogen, TNF*α*, IL-1*β*, and the *β*1 adrenergic receptor were associated with upstream nucleotide variants either in or adjacent to putative binding sites for transcription factors including Ebf1 and Tfap2. Single nucleotide variants in the vicinity of a TFBS could modulate transcription in a disease-specific manner. Hence, our data suggest that regulatory variants could influence the network dynamics and structure underlying autonomic dysfunction. In the context of our differential network analysis, all of these genes appeared to be differentially regulated in autonomic dysfunction. For example, adrenal angiotensinogen was shown to be activated by adrenal renin in autonomic dysfunction. However, our network identification analysis did not reveal evidence for the activation of adrenal angiotensinogen by adrenal renin in the control phenotype.

The transcription factor early B-cell factor-1 (Ebf1), also known as olfactory-1 (Olf1), was associated with a putative binding site adjacent to the disease-specific nucleotide variation upstream of the angiotensinogen and IL-1*β* genes. Ebf1 is reported to be expressed in several CNS regions and has been implicated in CNS function and development [102–105]. This factor has also been implicated in B-cell development and peripheral inflammation [106–108]. Furthermore, Ebf1 may exert a role in the pathophysiology of multiple sclerosis [109]. However, Ebf1 function has not been previously assessed in the context of cardiovascular disease, hypertension, or neuroinflammation. Both our data and the available published research support a role for Ebf1 in the mechanisms underlying autonomic dysfunction, thereby suggesting a novel hypothesis for a genetic contribution to autonomic dysfunction development.

Given our results, and the established connection between genetic variations and gene expression dynamics [110, 111], our novel hypotheses regarding the genetic correlates of autonomic dysfunction suggest a genetic basis for dynamic network modulation that can be leveraged for the design of therapeutic approaches. The translational relevance of animal model findings could be established by identifying whether overlapping cis-regulatory variants are both linked to human disease through genome wide association studies and implicated in regulation of organ-specific gene expression based on expression quantitative trait locus analysis [112]. Because inherited genetic variations can only be causes, as opposed to consequences, of gene expression dynamics, identifying genetic variations that regulate organ-specific functional processes could facilitate the identification of novel therapeutics [113]. It has been argued that incorporating information from human genetics in the drug discovery pipeline could mitigate against compound attrition [114]. Focused studies of animal models—which allow for the investigation of pathogenesis and temporal dynamics in multiple organs—could be combined with human genetics data to identify novel biomarkers therapeutic targets with enhanced potential.

Because our inferred signed directed networks exhibit extensive feedbacks, we make no explicit assumptions regarding the causal influences of a given network node. Each gene continuously regulates and is regulated by other genes. Compensatory responses to the initiating pathological events could be epiphenomena, or could further propagate regulatory influences on pathogenesis. Disambiguating the time-dependent regulatory control of homeostasis and response to disease-initiating events will be critical for defining the mechanisms of pathogenesis. Such issues can be addressed computationally through simulated perturbations or sensitivity analyses [115], and experimentally using pharmacological interventions or conditional knockouts [116]. It will also be critical to define the coupling between molecular events (e.g., gene/protein expression variations) and features of systemic homeostasis. Moreover, its is generally important to establish the role of initial conditions, independent of other regulatory features such as network structure, in coordinating the evolution of a system’s behavior [117].

It is noteworthy that apparent gene expression differences between the autonomic dysfunction and control conditions were observed at the earliest time point of our study, which preceded disease initiation. Hence, it could be argued that molecular network and gene expression trajectory differences between the autonomic dysfunction and control phenotypes could reflect genetic differences that are unrelated to disease pathogenesis. Because the autonomic dysfunction model utilized in our study recapitulates several features of human hypertension and metabolic dysfunction, the supposition that our findings reflect pathogenic mechanisms is not inconsistent with available evidence. It is a general shortcoming of currently available research on human hypertension that there are not more prospective longitudinal data available to delineate temporal mechanisms [24, 38]. It is generally a problem that the temporal trajectories and variability thereof across human populations is not well understood for complex diseases [118]. However, extensive focused analyses are required to disambiguate the relative interactions amongst genetics, molecular network structure, expression dynamics, physiological processes, and disease manifestation.

### Conclusion

Our process dynamics-based approach presents a novel experimental and analytic paradigm for the dissection of mechanisms underlying disease pathogenesis. Our analyses yielded several hypotheses for novel mechanisms of autonomic dysfunction development. Our dynamic and network analyses are based on a mathematical model that can be further utilized for simulations and computational analyses directed to unravel new disease mechanisms and optimize treatment strategies [94, 119]. Our data-driven model can also serve as a basis for identifying putative biomarkers for prediction of disease onset [20]. Future directions include refining the temporal resolution of the time series analysis, examining a broader range of ages, and utilizing an expanded repertoire of molecular and functional assays. Investigation into whether network interactions undergo re-wiring throughout the progression of disease [76] will be important for understanding the mechanisms underlying how therapeutic interventions can restore pathological molecular networks to a healthy state [120]. Targeted perturbations will be critical to confirming mechanistic influences of gene regulatory interactions. In sum, our novel integrated approach can be more broadly applied to examine the developmental dynamics of numerous chronic disease states. Such analyses are expected to yield novel mechanistic knowledge that can facilitate the identification of novel biomarkers and therapeutic treatment strategies based on the structure and dynamics of multi-organ interactions underlying organismal homeostasis.

## Supporting information

S1 Text This file contains supporting information. (PDF)

S1 File This file contains un-normalized raw gene expression data (Ct values). (CSV)

S2 File This file contains normalized gene expression data. (CSV)

S3 File This file contains normalized gene expression data plotted along with model simulation traces. (PDF)

S4 File This file contains names for parameters and dynamic variables. As an example to describe out parameter label convention, the interaction coefficient denoting the directed influence in which gene g2 from organ r2 regulates gene g1 in organ r1 is labled k_r1g1_r2g2 (i.e., k_to_from). Initial conditions are included in another tab. SHR denotes the spontaneously hypertensive rat (autonomic dysfunction) and WKY denotes the Wistar Kyoto control phenotype.
(XLSX)

S5 File This file contains the dynamic model for the autonomic dysfunction phenotype in the systems biology markup language (SBML) format. The model was converted from Matlab to SBML using *MOCCASIN* [121].
(XML)

S6 File This file contains the dynamic model for the control phenotype in the systems biology markup language (SBML) format. The model was converted from Matlab to SBML using *MOCCASIN* [121].
(XML)

S7 File This file contains the parameter values and initial conditions, along with some other basic information for simulating the autonomic dysfunction and control models in matlab. (MAT)

S8 File This file contains matlab simulation code. (M)

S9 File This file contains the parameter values and initial conditions for simulating the autonomic dysfunction and control models in R. (RDATA)

S10 File This file contains R simulation code. (R)

S1 Fig Sampling, normalization, and outlier evaluation. (A) Table detailing the animal sampling and organs utilized in our analysis for each animal. (B) Stability ranks of median expression values were considered for each organ/age combination. For the majority of organ/time combinations, the median expression level was ranked among the most stable (≤ 12/22), in comparison with the stability levels for individual genes. (C) PCA was applied to the entire data set (all genes/organs) and plotted along with the variability accounted for by the first two PCs. The smooth circle shows the 99% confidence interval for the mean of a bi-variate Gaussian distribution characterized by the displayed data. Note that this interval contains the majority of the data, and the few value outside of this interval are in close proximity. (D) PCA was implemented separately for each organ. Specific colors refer to the same animals in all plots. For instance, the three gray dots in the Adrenal PCA plot refer to three animals that are relatively distant from the other animal samples in this analysis. However, observation of the PC projections of these specific animals in the PCAs applied to the data from other organs shows that these animal samples are not imposing consistent biases. Panel (E) shows sample expression data labeled as in (D) for animal samples marked in the Adrenal and Ventricle PCAs.
(TIF)

S2 Fig Robustness of regularized regression-based system identification. Error between simulated gene expression levels and experimentally measured mean expression values varies minimally with respect to regularization parameters. Log error is plotted with respect to the log *λ* value for a range of *α* levels.
(TIF)

S3 Fig Evaluation of sign consistency of interaction coefficients across multiple iterations of system identification. The equation illustrates the computation of the odds ratio based on the contingency table.
(TIF)

S4 Fig Differential network analysis of changes in gene-gene interactions in autonomic dysfunction. Black bars correspond to edges considered to be differentially regulated in autonomic dysfunction.
(TIF)

S5 Fig Timeseries analysis of gene expression dynamics. Many genes showed significantly different expression patterns between autonomic dysfunction and control phenotypes (q < 0.1, -log q > 1).
(TIF)

S6 Fig Correlational analysis of system identification robustness. High correlations (> 0.7) between identified networks were observed over an expansive range of regularization parameter space. (A) Spearman rank correlation coefficient histogram and (B) Correlation values as a function of regularization parameter values for *λ* and *α*.
(TIF)

S7 Fig Graph theoretic analysis of network identification robustness. (A) Path length, (B) clustering coefficients, and (C) power law exponents are shown for a range of regularization parameters.
(TIF)

S8 Fig Graphical representations of network interactions. (A) Phenotype-specific multi-organ networks. (B) Subnetworks including interactions between the brainstem and adrenal gland.
(TIF)

S9 Fig Network illustrating influences of the brainstem on the other organs in the autonomic dysfunction phenotype. Note that the nodes are organized as in S10 Fig for comparison.
(TIF)

S10 Fig Network illustrating influences of the brainstem on the other organs in the control phenotype. Note that the nodes are organized as in S9 Fig for comparison.
(TIF)

S11 Fig Organized sequence of gene expression valleys in autonomic dysfunction. Expression profiles were organized according to the sequence of valleys observed for the autonomic dysfunction phenotype (left).
(TIF)

S12 Fig Organized sequence of gene expression peaks is disrupted in the autonomic dysfunction phenotype. Expression profiles were organized according to the sequence of peaks observed for the control phenotype (left).
(TIF)

S13 Fig Organized sequence of gene expression valleys is disrupted in the autonomic dysfunction phenotype. Expression profiles were organized according to the sequence of valleys observed for the control phenotype (left).
(TIF)

S14 Fig Dynamics comparison for autonomic dysfunction and control phenotypes. Genes are shown that exhibit (A) peaks in both phenotypes, (B) peaks in autonomic dysfunction but valleys for the control phenotype, (C) valleys for autonomic dysfunction but peaks for the control phenotype, and (D) valleys for both phenotypes. Straight black lines correspond to the unity line. (E) Conceptual overview of the profiles observed in panel (A, peaks on both axes) and panel (D, valleys on both axes). The top left quadrant of panel (E) shows two sets of profiles: in the first, the control profile shows an early peak while the disease profile shows a late peak; in the second, the control shows an early valley and the disease profile shows a late valley. Respectively, these two profiles in the upper left quadrant of panel (E) correspond to the upper left quadrants of panels (A) and (D). These sets of profiles correspond to preserved waveforms but temporal shifts between the expression in control versus disease phenotypes. Panel (F) can be interpreted as for panel (E). Each quadrant of (F) exhibits pairs of dynamic profiles corresponding to either panel (B, top pair) or (C, bottom pair). The extreme off-diagonal profiles depict instances in which the dynamics patterns are inverted for disease relative to control.
(TIF)

S15 Fig Control brainstem network. This representation is shown for comparison with main text Fig 7A.
(TIF)

S16 Fig Autonomic dysfunction brainstem feedforward motifs. All three node feedforward motifs were identified by motif analysis.
(TIF)

S17 Fig Example three node network with inconsistent kinetics. (A) Network motif and (B) simulation traces.
(TIF)

S18 Fig Autonomic dysfunction-specific SNVs in regulatory regions. Motif signatures for transcription factors and spatial proximities between TFBSs, TSSs, and SNVs.
(TIF)
