# Supplementary material for: A data-driven modeling approach to identify disease-specific multi-organ networks driving physiological dysregulation
Source: PLoS Comput Biol. 2017 Jul 21;13(7):e1005627. doi: 10.1371/journal.pcbi.1005627 (PMC5521738; doi:10.1371/journal.pcbi.1005627)
Supplement: S1 Text — (PDF) [file pcbi.1005627.s001.pdf]

A data-driven modeling approach to identify disease-specific multi-organ networks driving physiological dysregulation

Warren D. Anderson[1◑¤], Danielle DeCicco[1◑], James S. Schwaber[1], Rajanikanth Vadigepalli[1*]

1 Daniel Baugh Institute for Functional Genomics and Computational Biology, Department of Pathology, Anatomy, and Cell Biology, Sidney Kimmel Medical College, Thomas Jefferson University, Philadelphia, PA, USA

◑These authors contributed equally to this work.
¤Current Address: Center for Public Health Genomics, University of Virginia, Charlottesville, VA, USA
* Corresponding author: rajanikanth.vadigepalli@jefferson.edu

Supporting Information

- Supplementary Files
- Supplementary Figures
- Supplementary Table

Supplementary Files

S1 File *S1 File.csv*: This file contains un-normalized raw gene expression data (Ct values).

S2 File *S2 File.csv*: This file contains normalized gene expression data.

S3 File *S3 File.pdf*: This file contains normalized gene expression data plotted along with model simulation traces.

S4 File *S4 File.xlsx*: This file contains names for parameters and dynamic variables. As an example to describe out parameter label convention, the interaction coefficient denoting the directed influence in which gene g2 from organ r2 regulates gene g1 in organ r1 is labled `k_r1g1_r2g2` (i.e., `k_to_from`). Initial conditions are included in another tab. SHR denotes the spontaneously hypertensive rat (autonomic dysfunction) and WKY denotes the Wistar Kyoto control phenotype.

S5 File *S5 File.xml*: This file contains the dynamic model for the autonomic dysfunction phenotype in the systems biology markup language (SBML) format. The model was converted from Matlab to SBML using *MOCCASIN* [1].

S6 File *S6 File.xml*: This file contains the dynamic model for the control phenotype in the systems biology markup language (SBML) format. The model was converted from Matlab to SBML using *MOCCASIN* [1].

S7 File *S7 File.mat*: This file contains the parameter values and initial conditions, along with some other basic information for simulating the autonomic dysfunction and control models in matlab.

S8 File *S8 File.m*: This file contains matlab simulation code.

S9 File *S9 File.RData*: This file contains the parameter values and initial conditions for simulating the autonomic dysfunction and control models in R.

S10 File *S10 File.R*: This file contains R simulation code.

Supplementary Figures

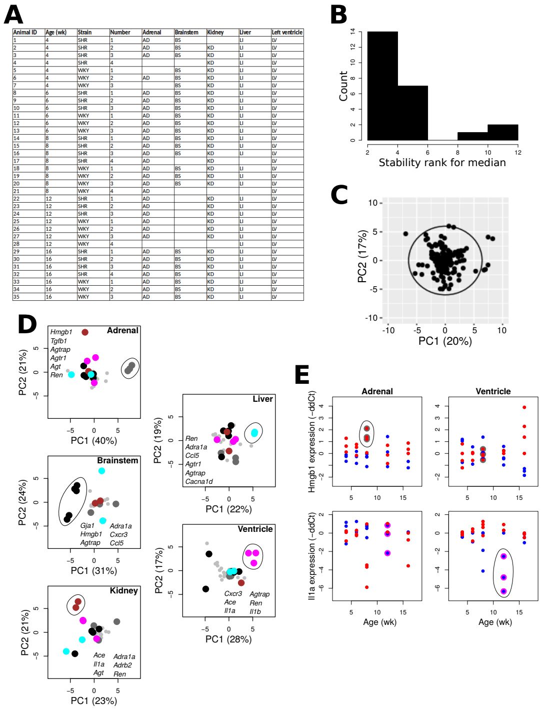

Animal ID	Age (wk)	Strain	Number	Adrenal	Brainstem	Kidney	Liver	Left ventricle
1	4	SHR	1	AD	BS		LI	LV
2	4	SHR	2	AD	BS	KD	LI	LV
3	4	SHR	3	AD	BS	KD	LI	LV
4	4	SHR	4			KD	LI	LV
5	4	WKY	1		BS	KD	LI	LV
6	4	WKY	2	AD	BS	KD	LI	LV
7	4	WKY	3		BS	KD	LI	LV
8	6	SHR	1	AD	BS	KD	LI	LV
9	6	SHR	2	AD	BS	KD	LI	LV
10	6	SHR	3	AD	BS	KD	LI	LV
11	6	WKY	1	AD	BS	KD	LI	LV
12	6	WKY	2	AD	BS	KD	LI	LV
13	6	WKY	3	AD	BS	KD	LI	LV
14	8	SHR	1	AD	BS	KD	LI	LV
15	8	SHR	2	AD	BS	KD	LI	LV
16	8	SHR	3	AD	BS	KD	LI	LV
17	8	SHR	4	AD		KD		LV
18	8	WKY	1	AD	BS	KD	LI	LV
19	8	WKY	2	AD	BS	KD	LI	LV
20	8	WKY	3	AD	BS	KD	LI	LV
21	8	WKY	4	AD				LV
22	12	SHR	1	AD		KD	LI	LV
23	12	SHR	2	AD		KD	LI	LV
24	12	SHR	3	AD		KD	LI	LV
25	12	WKY	1	AD		KD	LI	LV
26	12	WKY	2	AD		KD	LI	LV
27	12	WKY	3	AD		KD	LI	LV
28	12	WKY	4				LI	LV
29	16	SHR	1	AD	BS	KD	LI	LV
30	16	SHR	2	AD	BS	KD	LI	LV
31	16	SHR	3	AD	BS	KD	LI	LV
32	16	SHR	4	AD	BS	KD	LI	LV
33	16	WKY	1	AD	BS	KD	LI	LV
34	16	WKY	2	AD	BS	KD	LI	LV
35	16	WKY	3	AD	BS	KD	LI	LV

Fig S1. Sampling, normalization, and outlier evaluation. (A) Table detailing the amimal sampling and organs utilized in our analysis for each animal. (B) Stability ranks of median expression values were considered for each organ/age combination. For the majority of organ/time combinations, the median expression level was ranked among the most stable ($\leq 12/22$), in comparison with the stability levels for individual genes. (C) PCA was applied to the entire data set (all genes/organs) and plotted along with the variability accounted for by the first two PCs. The smooth circle shows the 99% confidence interval for the mean of a bi-variate Gaussian distribution characterized by the displayed data. Note that this interval contains the majority of the data, and the few value outside of this interval are in close proximity. (D) PCA was implemented separately for each organ. Specific color refer to the same animals in all plots. For instance, the three gray dots in the Adrenal PCA plot refer to three animals that are relatively distant from the other animal samples in this analysis. However, observation of the PC projections of these specific animals in the PCAs applied to the data from other organs shows that these animal samples are not imposing consistent biases. Panel (E) shows sample expression data labeled as in (D) for animal samples marked in the Adrenal and Ventricle PCAs.

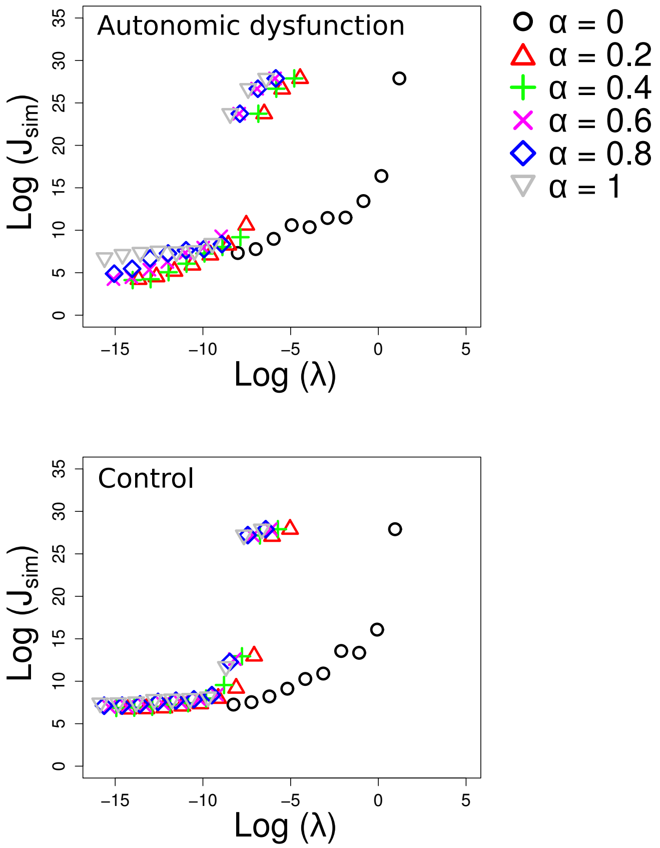

Fig S2. Robustness of regularized regression-based system identification. Error between simulated gene expression levels and experimentally measured mean expression values varies minimally with respect to regularization parameters. Log error is plotted with respect to the log λ value for a range of α levels.

$K_{best} < 0$ and $K_{comp} > 0$	$K_{best} > 0$ and $K_{comp} > 0$
$K_{best} < 0$ and $K_{comp} < 0$	$K_{best} > 0$ and $K_{comp} < 0$

$$\text{Odds ratio} = \frac{\dfrac{K_{best} < 0 \text{ and } K_{comp} > 0}{K_{best} > 0 \text{ and } K_{comp} > 0}}{\dfrac{K_{best} < 0 \text{ and } K_{comp} < 0}{K_{best} > 0 \text{ and } K_{comp} < 0}}$$

Fig S3. Evaluation of sign consistency of interaction coefficiencts across multiple iterations of system identification. The equation illustrates the computation of the odds ratio based on the contingency table.

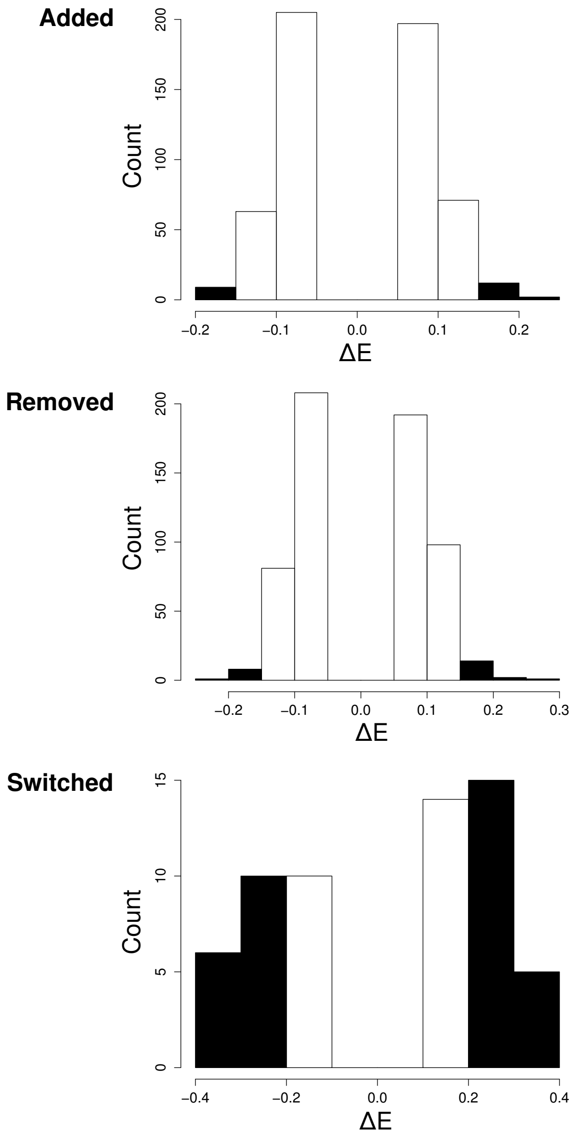

Fig S4. Differential network analysis of changes in gene-gene interactions in autonomic dysfunction. Black bars correspond to edges considered to be differentially regulated in autonomic dysfunction.

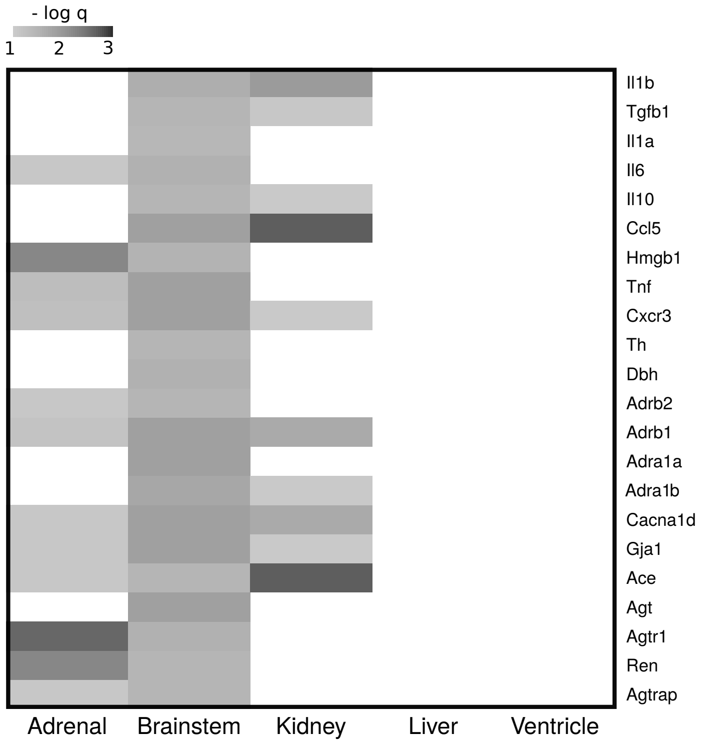

Fig S5. Timeseries analysis of gene expression dynamics. Many genes showed significantly different expression patterns between autonomic dysfunction and control phenotypes ($q < 0.1$, $-\log q > 1$).

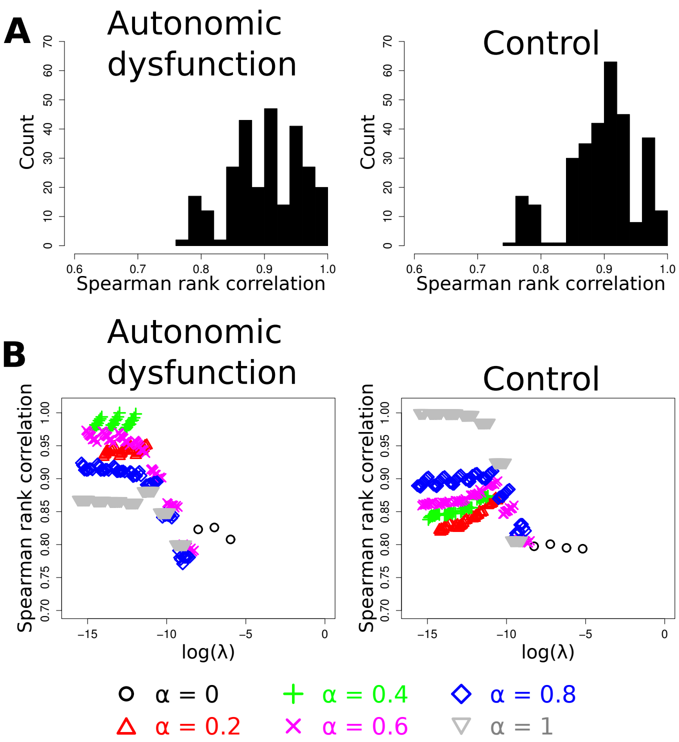

Fig S6. Correlational analysis of system identification robustness. High correlations (> 0.7) between identified networks were observed over an expansive range of regularization parameter space. (A) Spearman rank correlation coefficient histogram and (B) Correlation values as a function of regularization parameter values for λ and α.

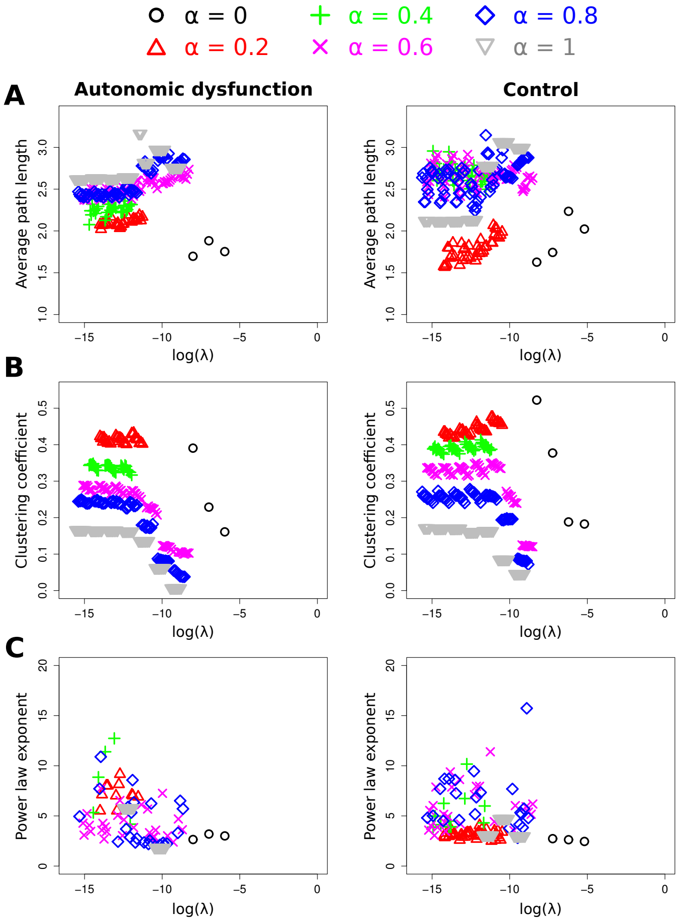

Fig S7. Graph theoretic analysis of network identification robustness. (A) Path length, (B) clustering coefficients, and (C) power law exponents are shown for a range of regularization parameters.

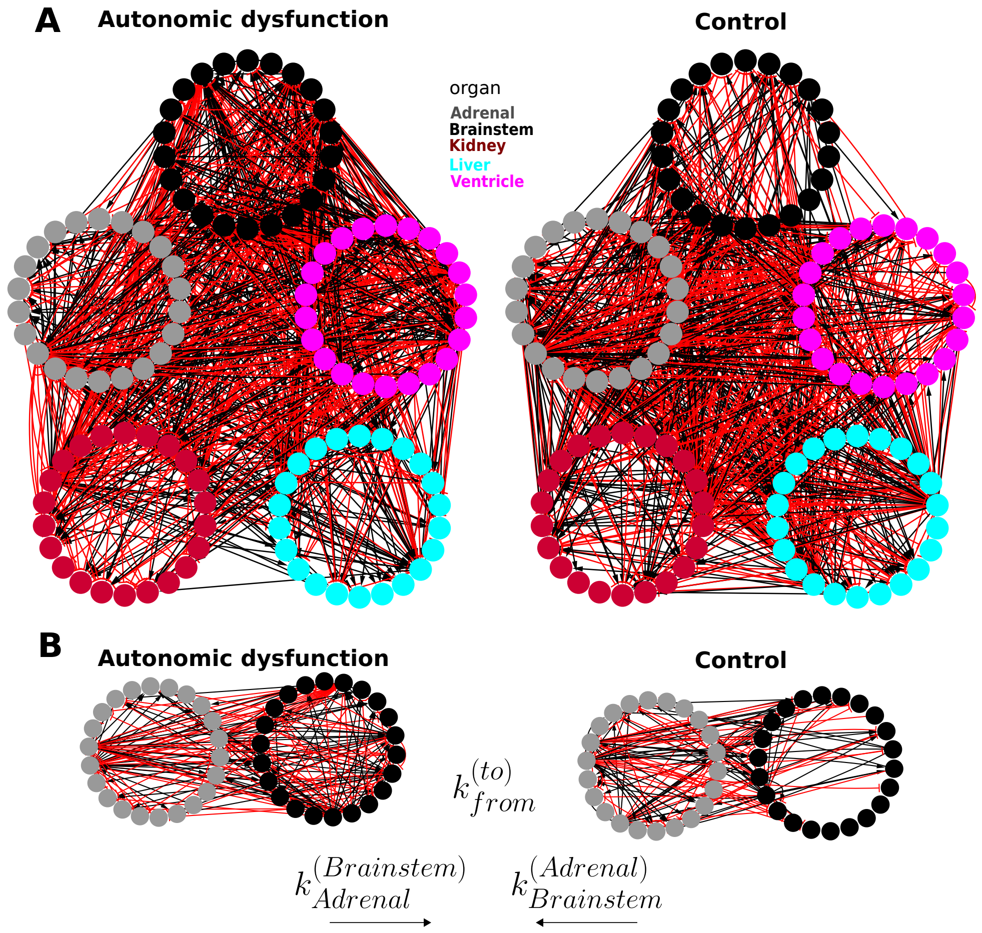

Fig S8. Graphical representations of network interactions. (A) Phenotype-specific multi-organ networks. (B) Subnetworks including interactions between the brainstem and adrenal gland.

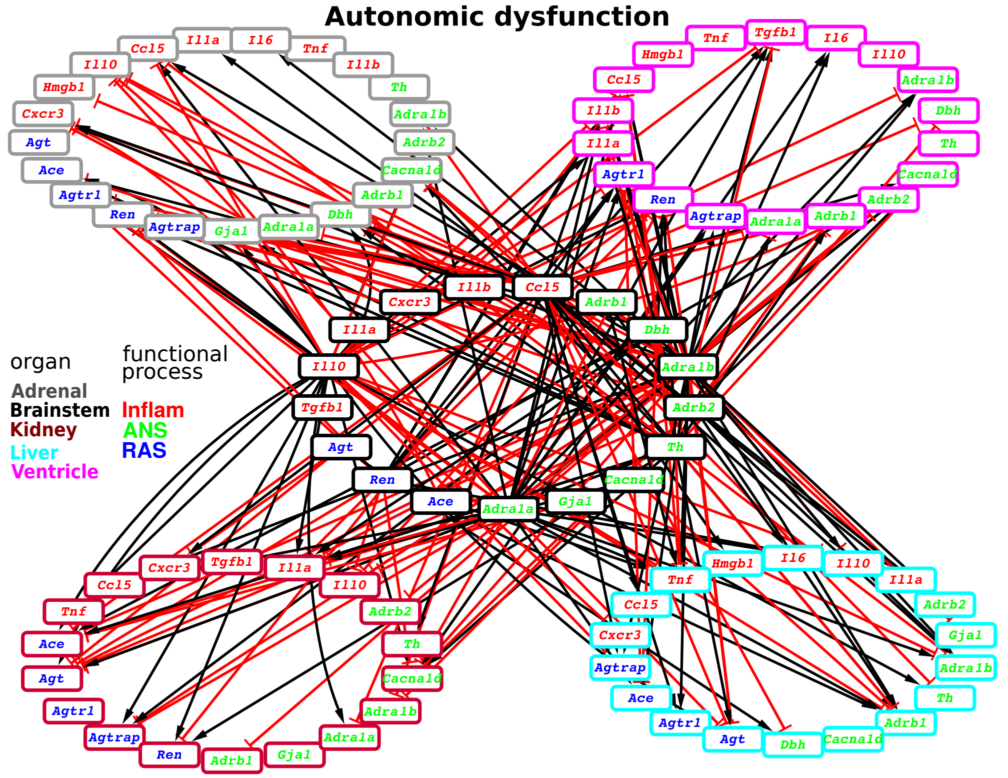

Fig S9. Network illustrating influences of the brainstem on the other organs in the autonomic dysfunction phenotype. Note that the nodes are organized as in Fig S10 for comparison.

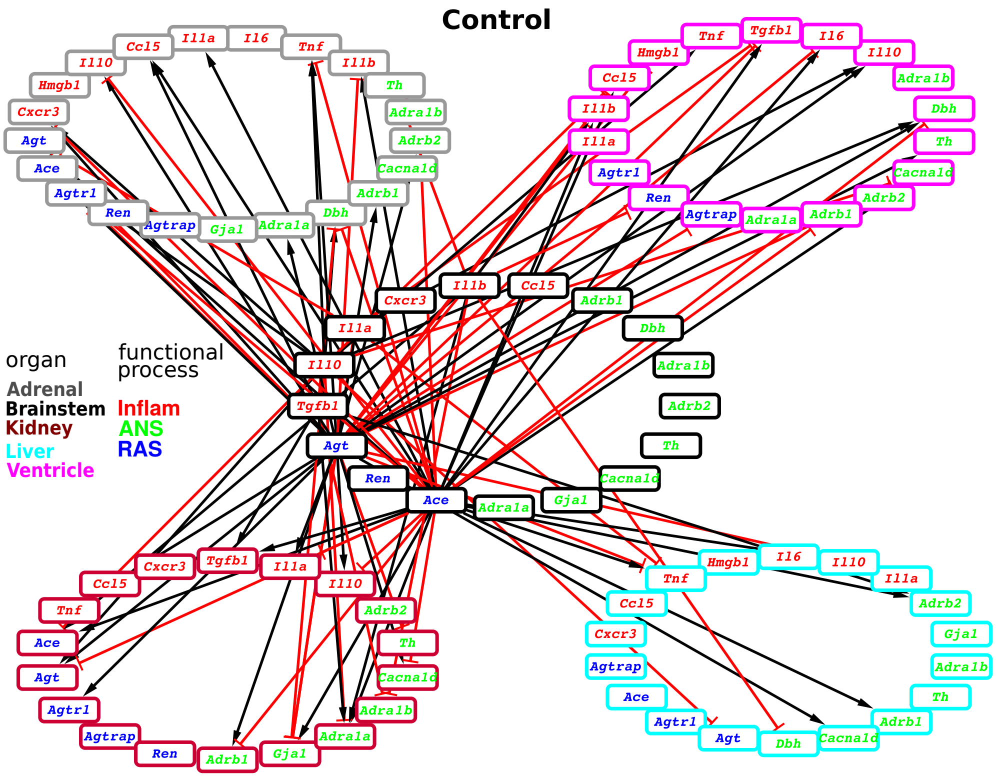

Fig S10. Network illustrating influences of the brainstem on the other organs in the control phenotype. Note that the nodes are organized as in Fig S9 for comparison.

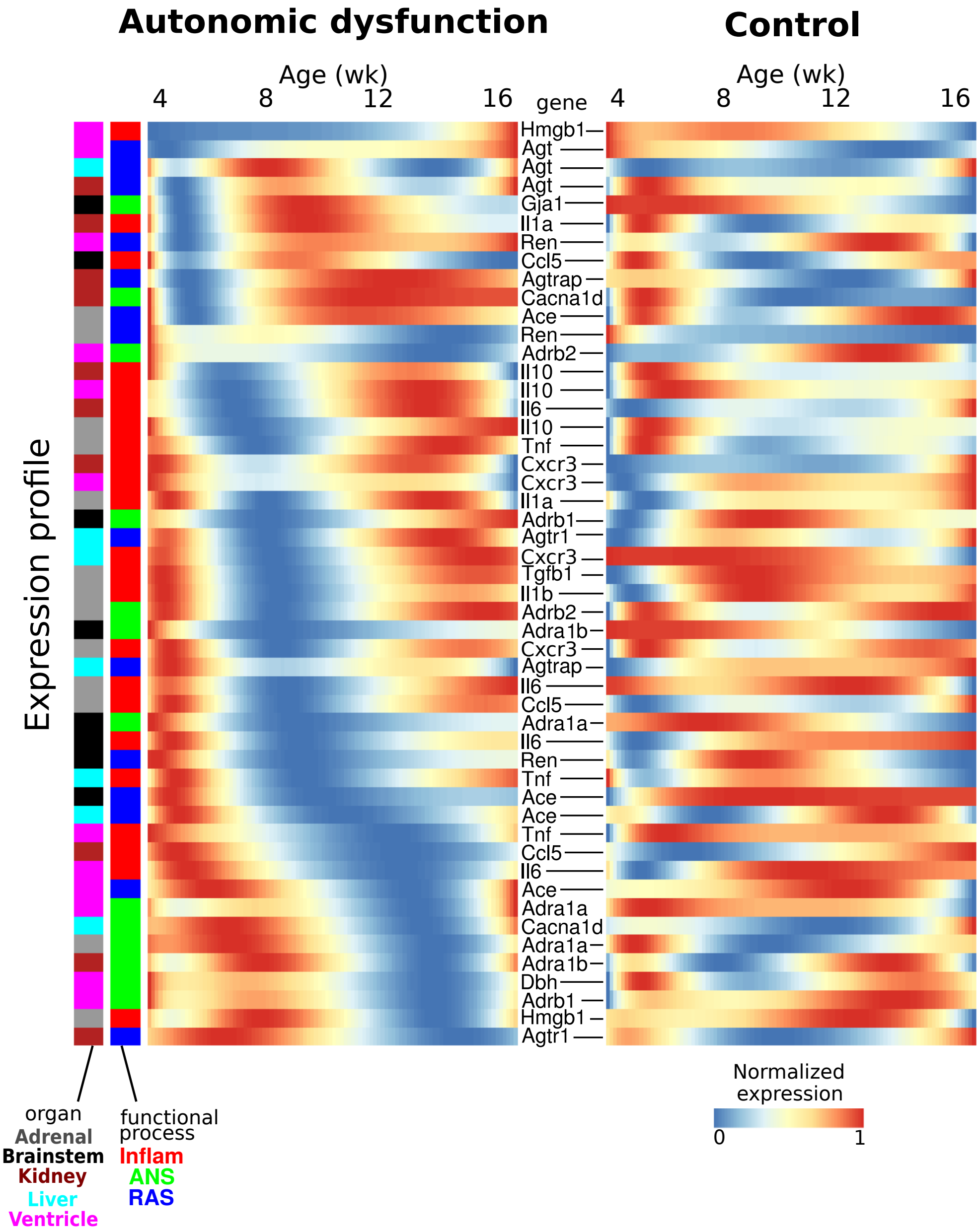

Fig S11. Organized sequence of gene expression valleys in autonomic dysfunction. Expression profiles were organized according to the sequence of valleys observed for the autonomic dysfunction phenotype (left).

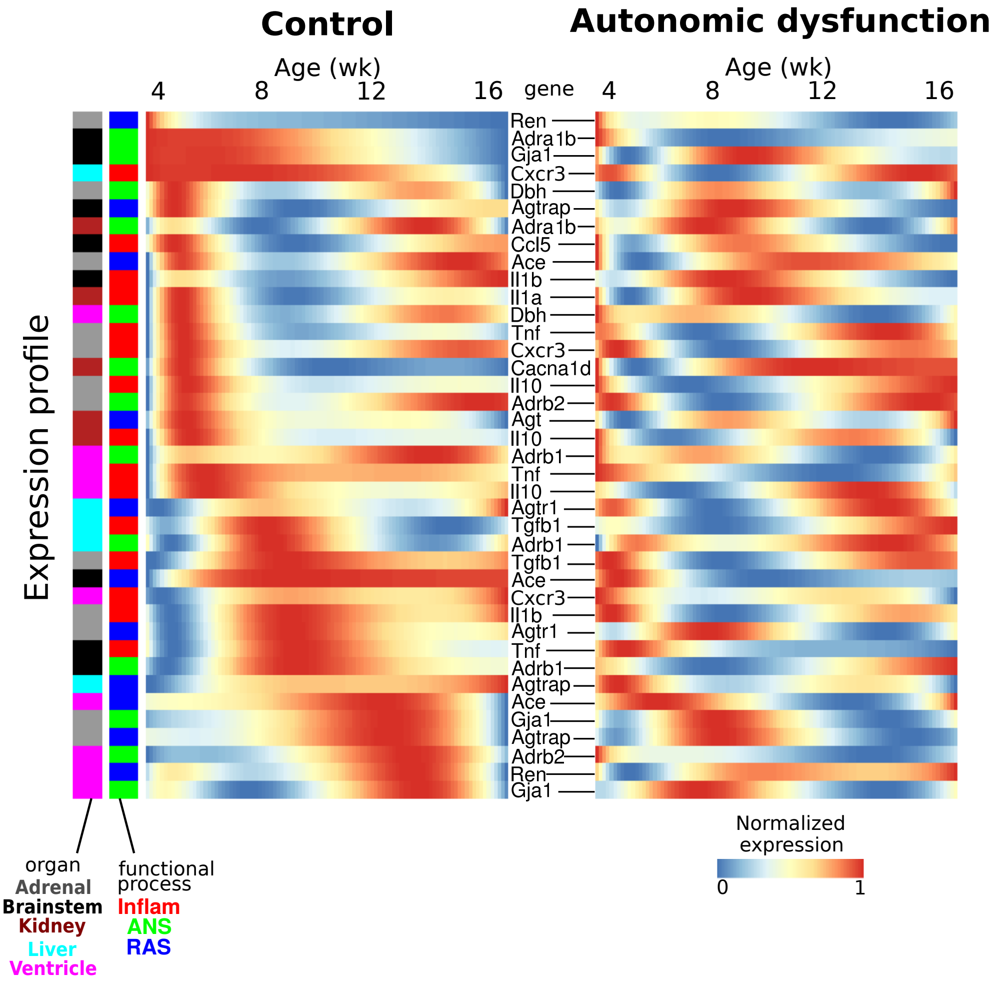

Fig S12. Organized sequence of gene expression peaks is disrupted in the autonomic dysfunction phenotype. Expression profiles were organized according to the sequence of peaks observed for the control phenotype (left).

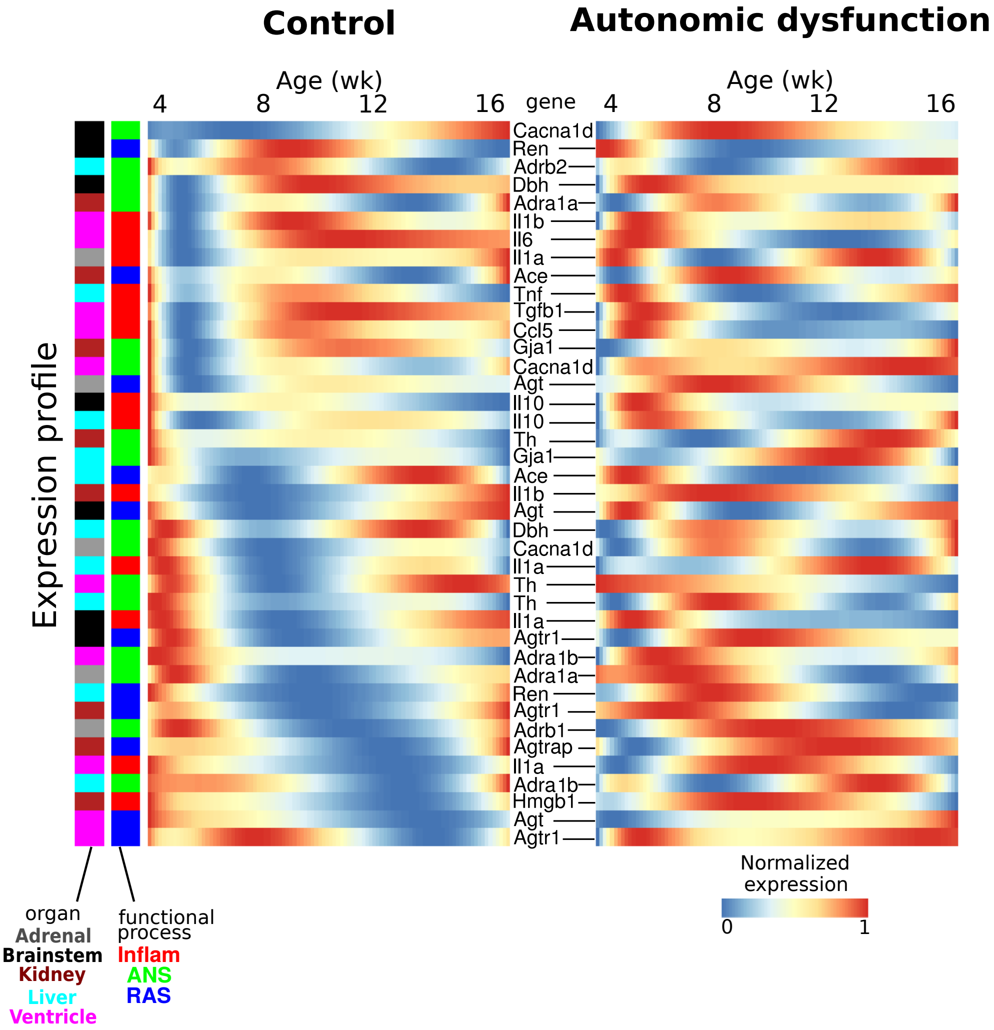

Fig S13. Organized sequence of gene expression valleys is disrupted in the autonomic dysfunction phenotyppe. Expression profiles were organized according to the sequence of valleys observed for the control phenotype (left).

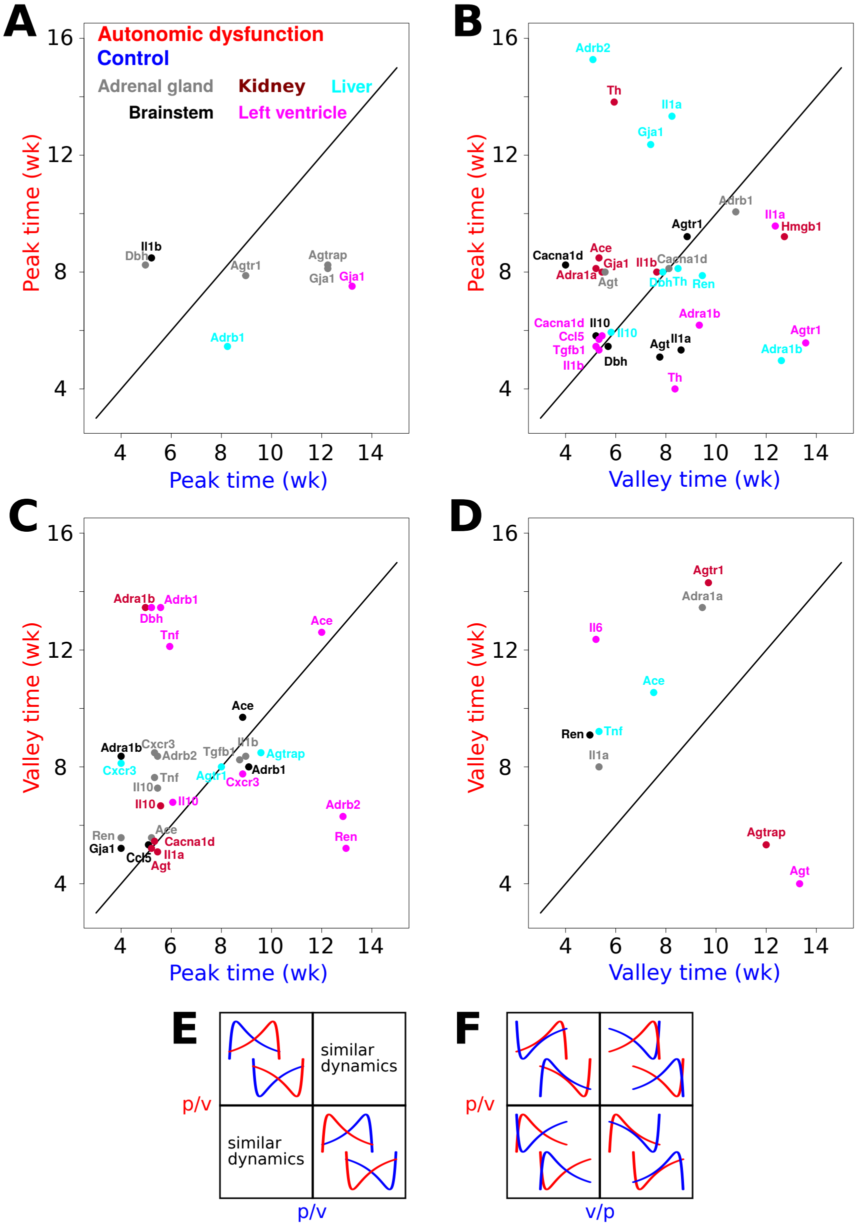

Fig S14. Dynamics comparison for autonomic dysfunction and control phenotypes. Genes are shown that exhibit (A) peaks in both phenotypes, (B) peaks in autonomic dysfunction but valleys for the control phenotype, (C) valleys for autonomic dysfunction but peaks for the control phenotype, and (D) valleys for both phenotypes. Straight black lines correspond to the unity line. (E) Conceptual overview of the profiles observed in panel (A, peaks on both axes) and panel (D, valleys on both axes). The top left quadrant of panel (E) shows two sets of profiles: in the first, the control profile shows an early peak while the disease profile shows a late peak; in the second, the control shows an early valley and the disease profile shows a late valley. Respectively, these two profiles in the upper left quadrant of panel (E) correspond to the upper left quadrants of panels (A) and (D). These sets of profiles correspond to preserved waveforms but temporal shifts between the expression in control versus disease phenotypes. Panel (F) can be interpreted as for panel (E). Each quadrant of (F) exhibits pairs of dynamic profiles corresponding to either panel (B, top pair) or (C, bottom pair). The extreme off-diagonal profiles depict instances in which the dynamics patterns are inverted for disease relative to control.

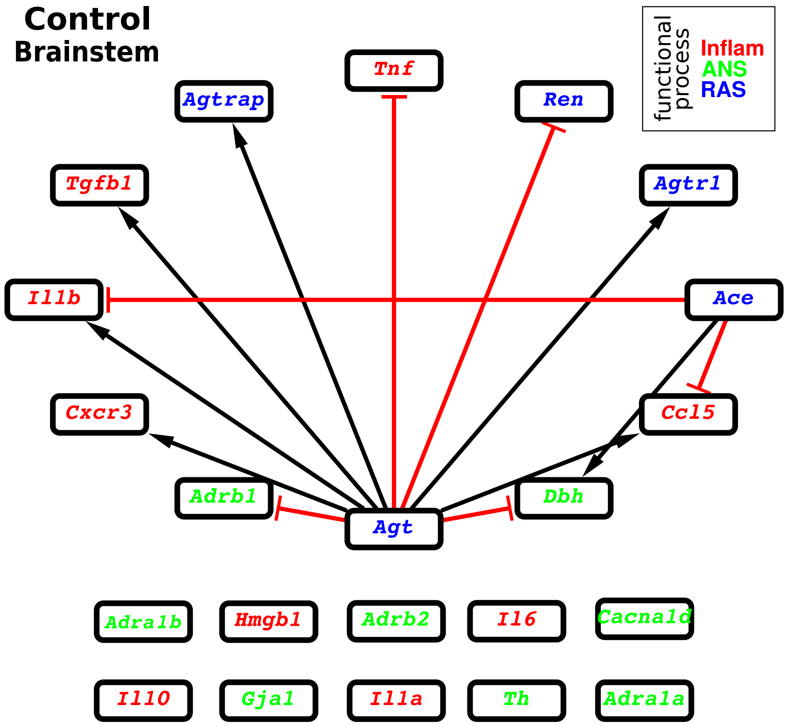

Fig S15. Control brainstem network. This representation is shown for comparison with main text Figure 7A.

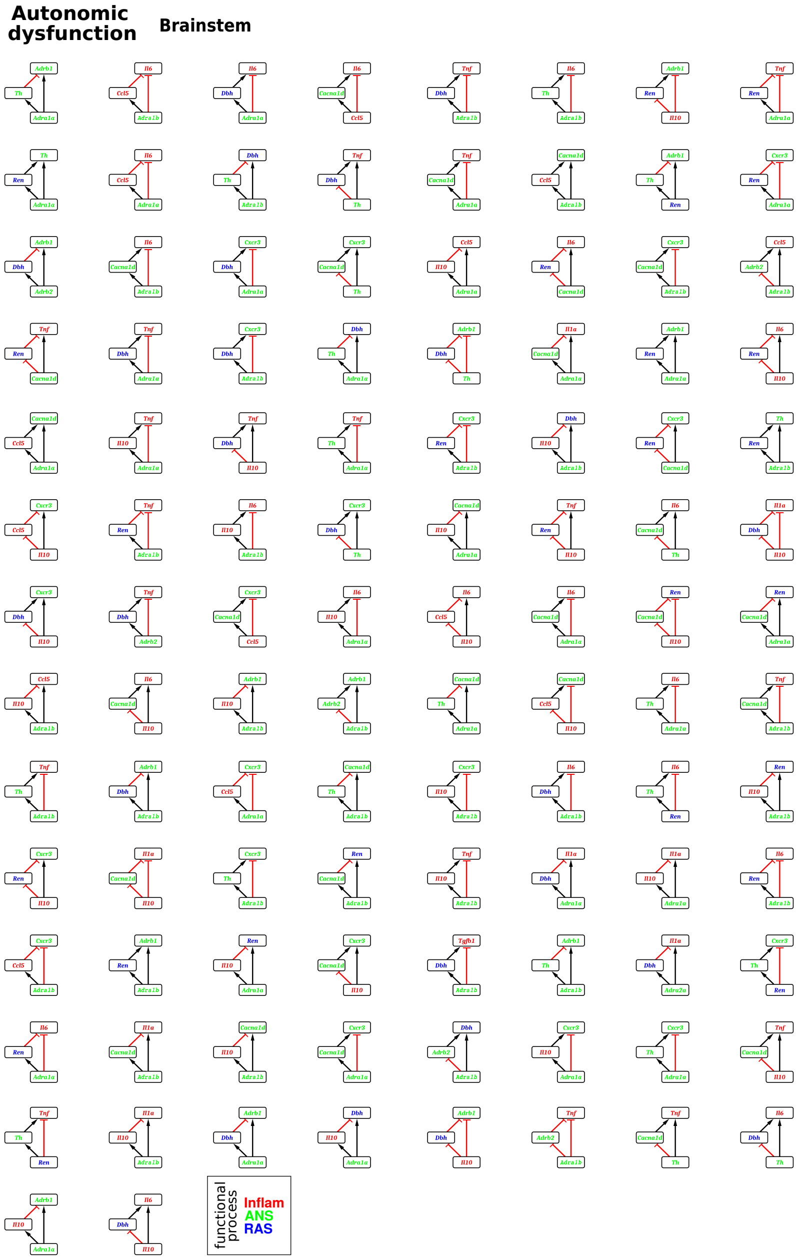

Fig S16. Autonomic dysfunction brainstem feedforward motifs. All three node feedforward motifs were identified by motif analysis.

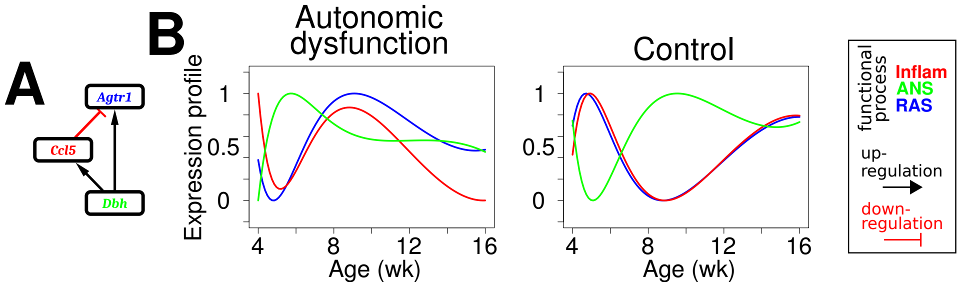

Fig S17. Example three node network with inconsistent kinetics. (A) Network motif and (B) simulation traces.

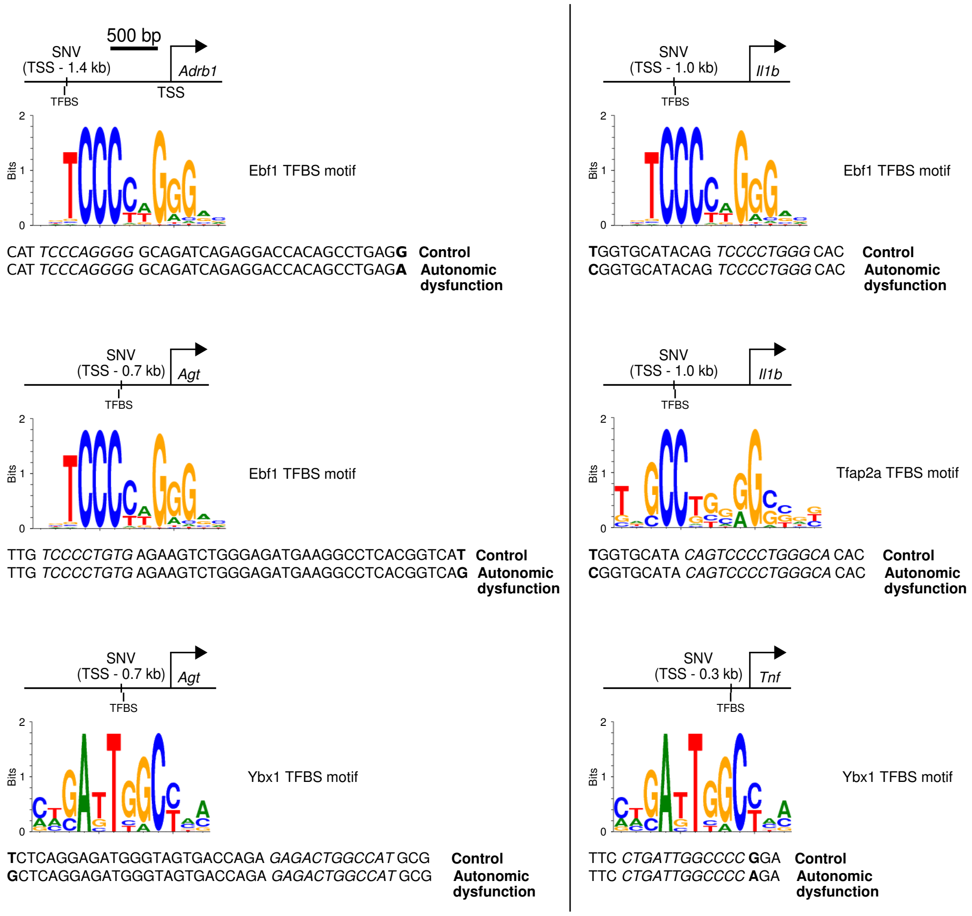

Fig S18. Autonomic dysfunction-specific SNVs in regulatory regions. Motif signatures for transcription factors and spatial proximities between TFBSs, TSSs, and SNVs.

Supplementary Table

Table 1. Primer sequences

Gene	ID	Sequence (5' - 3')
Ace	NM_012544	f: GACAACTATCCAGAGGGAATTGA
		r: CACAACACCTTGGCTGTCC
Actb	NM_031144	f: CTGGCTCCTAGCACCATGA
		r: TAGAGCCACCAATCCACACA
Adra1a	NM_017191	f: CACTTCTCAGTGAGGCTGCT
		r: AGGCTTGAAATCCGGGAAGA
Adra1b	NM_016991	f: TTCTTCATCGCTCTCCCGCT
		r: GCTGTTGAAGTAGCCCAGCC
Adrb1	NM_012701	f: AGACGTGCTATGTGTGACGG
		r: CTCTGGTAGCGAAAGGGCAG
Adrb2	NM_012492	f: GTGGATTGTGTCGGGCCTTA
		r: GCGATAGCATAGGCCTGGTT
Agt	NM_134432	f: CACCTACGTTCACTTCCAAGG
		r: AGAACTCATGGAGCCCAGTC
Agtr1a	NM_030985	f: CTCTCTCAGCTCTGCCACATTC
		r: TTCGAAATCCACTTGACCTGGTG
Agtrap	NM_001007654	f: ATTGGCATGTTTCTTGGTGGC
		r: CAACGGCAACGCTTGAGTAG
Cacna1d	NM_017298	f: GGCAGAAGACATAGATCCTGAGA
		r: ACTGGTGGGCATGCTAGTGT
Ccl5	NM_031116	f: GTGCCCACGTGAAGGAGTAT
		r: TCGAGTGACAAAGACGACTGC
Cxcr3	NM_053415	f: TAGATGCCTCGGACATTGCC
		r: AGGAGGCTGTAGAGGACTGG
Dbh	NM_013158	f: ACTACTGTCGCCACGTGCT
		r: ACCGGCTTCTTCTGGGTAGT
Gja1	NM_012567	f: ACTTCAGCCTCCAAGGAGTTC
		r: CATGTCTGGGCACCTCTCTTT
Hmgb1	NM_012963	f: GGCGGCTGTTTTGTTGACAT
		r: ACCCAAAATGGGCAAAAGCA
Il1a	NM_017019	f: AGGATCGTCAAGCAGGAGTT
		r: TTTAGAGTCGTCTCCTCCCGA
Il1b	NM_031512	f: AGGCTGACAGACCCCAAAAG
		r: CTCCACGGGCAAGACATAGG
Il6	NM_012589	f: TCTGGTCTTCTGGAGTTCCG
		r: AGCATTGGAAGTTGGGGTAGG
Il10	NM_012854	f: TTGAACCACCCGGCATCTAC
		r: CCAAGGAGTTGCTCCCGTTA
Ren	NM_012642	f: GCCAGCTTTGGACGAATCTT
		r: CCCCATTCAGCACTGATCCT
Tgfb1	NM_021578	f: TGGAAAGGGCTCAACACCTG
		r: AGAAGTTGGCATGGTAGCCC
Th	NM_012740	f: GCCTGTGTACTTTGTGTCCGAGAG
		r: TACGAGAGGCATAGTTCCTGAGC
Tnf	NM_012675	f: GTCGTAGCAAACCACCAAGC
		r: TGTGGGTGAGGAGCACATAG

References

1. Gómez HF, Hucka M, Keating SM, Nudelman G, Iber D, Sealfon SC. MOCCASIN: converting MATLAB ODE models to SBML. Bioinformatics (Oxford, England). 2016;32(12):1905–1906.
