# Supplementary figures and images for: A data-driven modeling approach to identify disease-specific multi-organ networks driving physiological dysregulation
Source: PLoS Comput Biol. 2017 Jul 21;13(7):e1005627. doi: 10.1371/journal.pcbi.1005627 (PMC5521738; doi:10.1371/journal.pcbi.1005627)

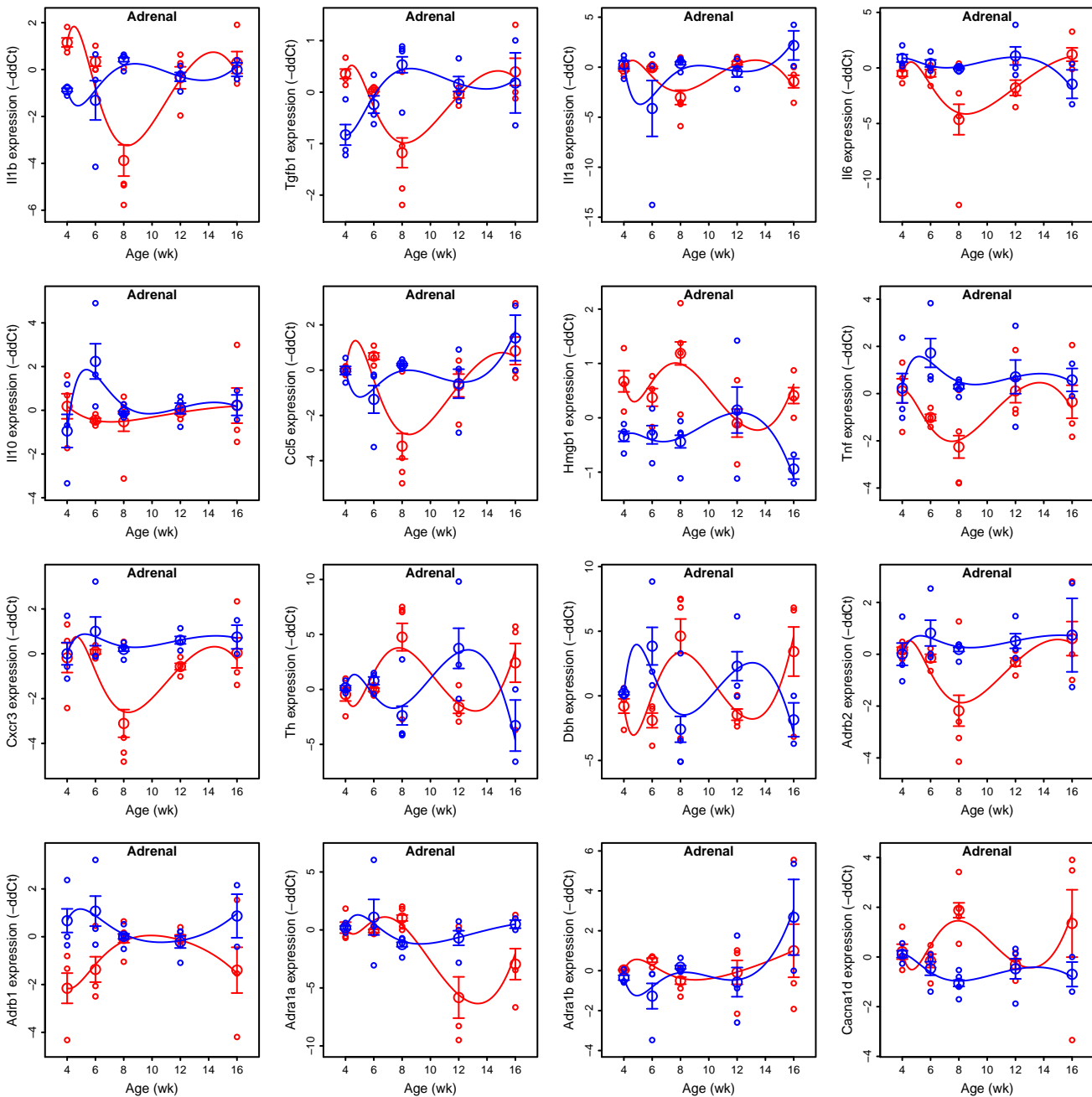

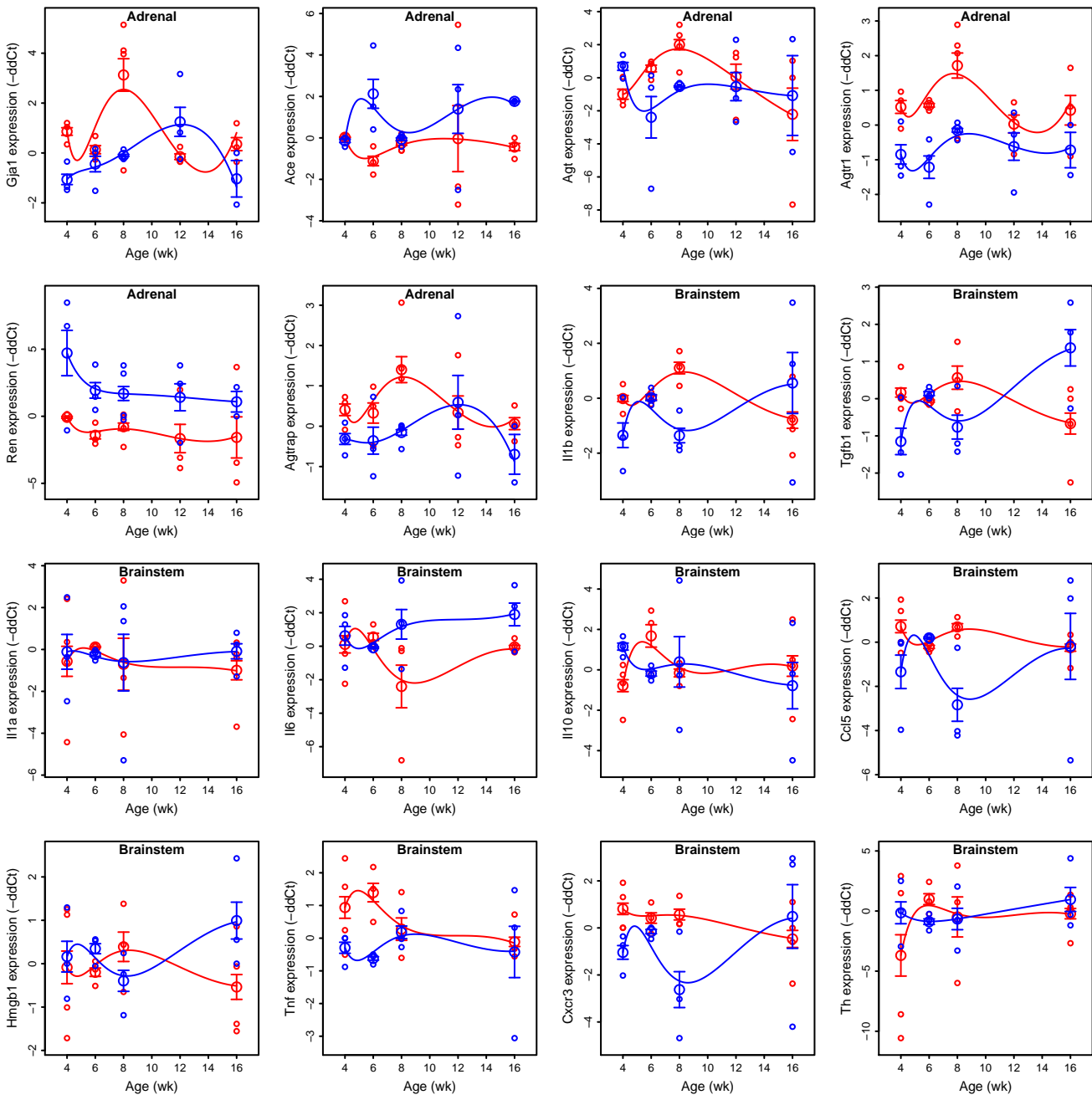

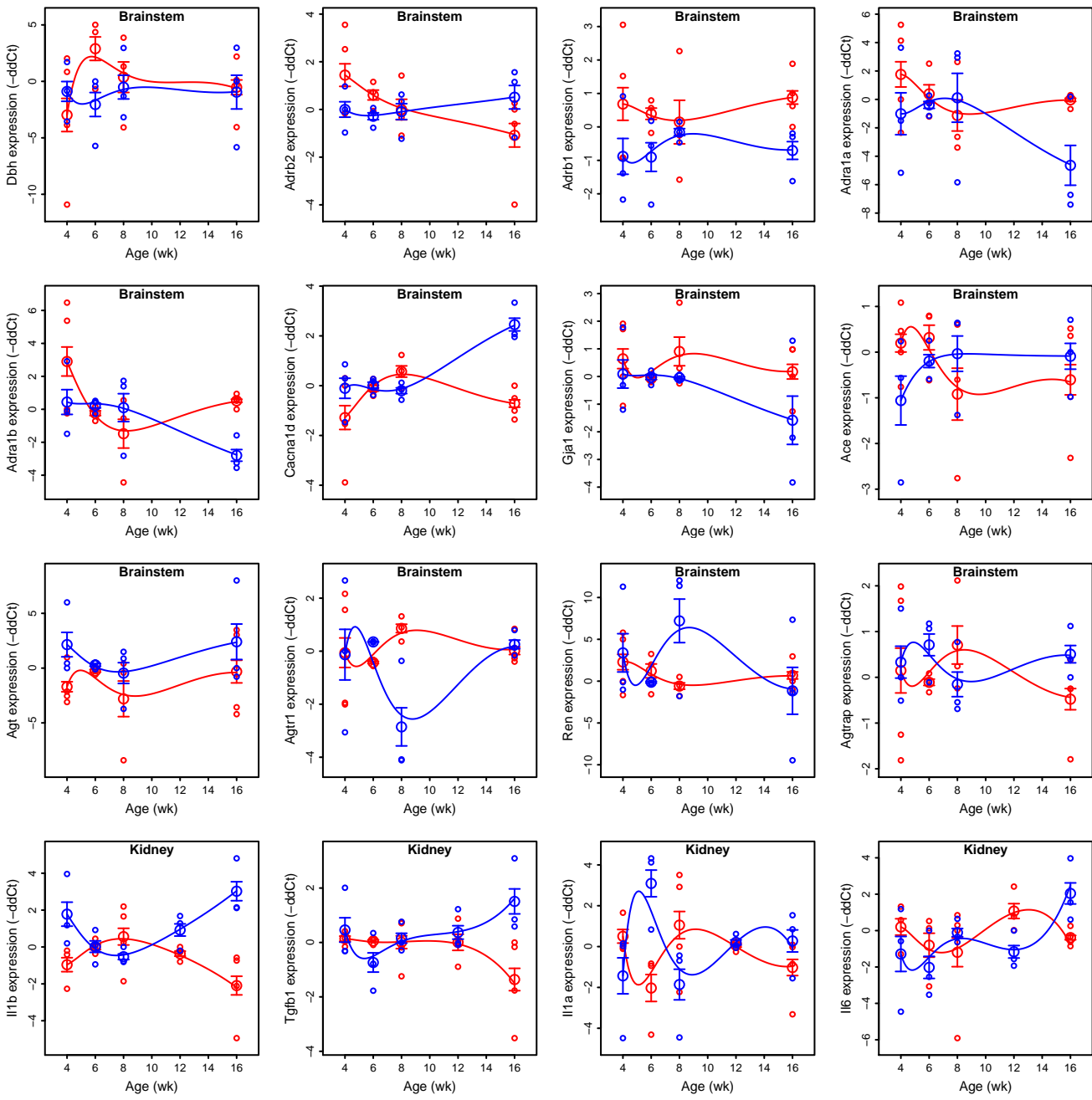

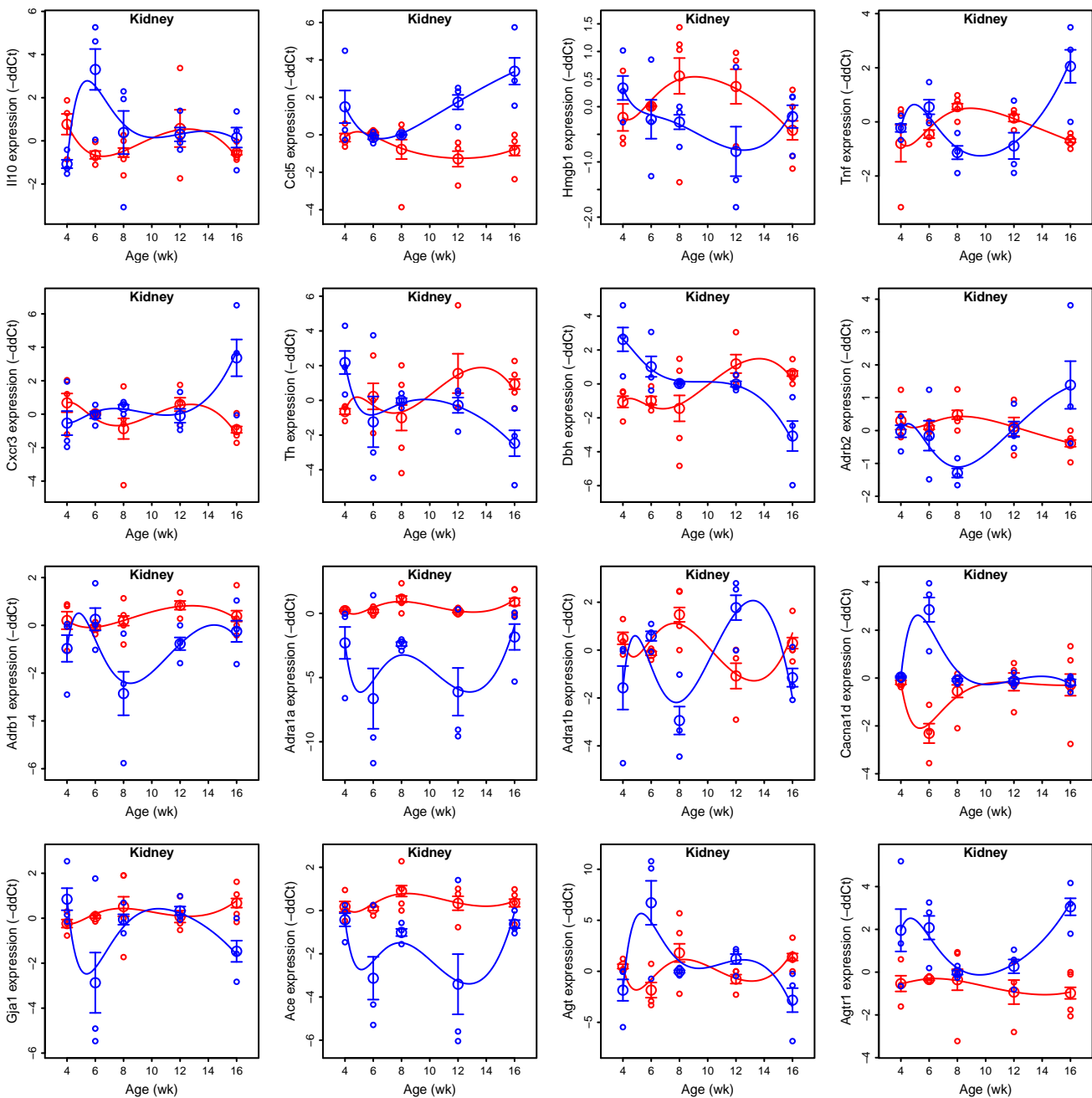

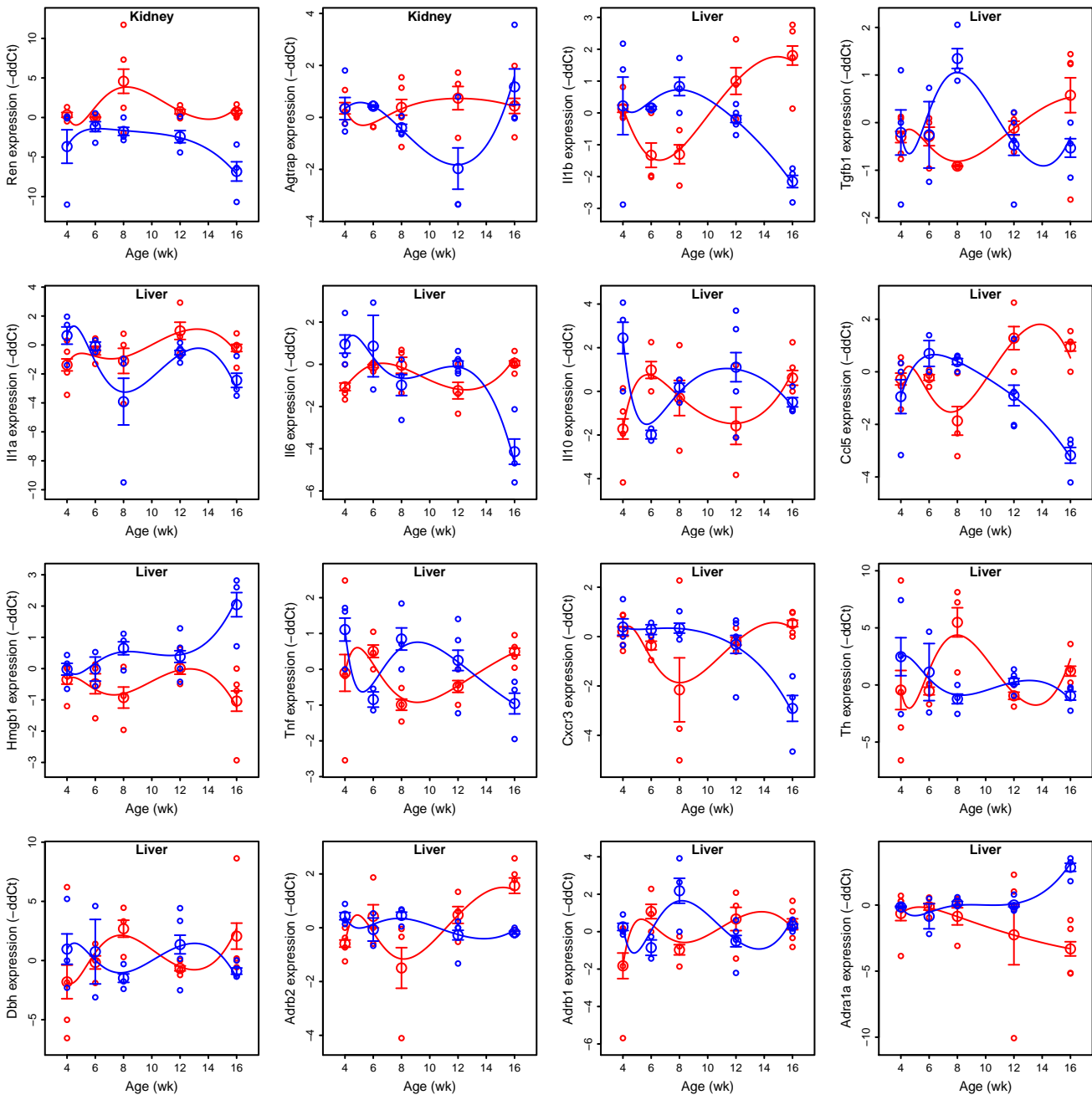

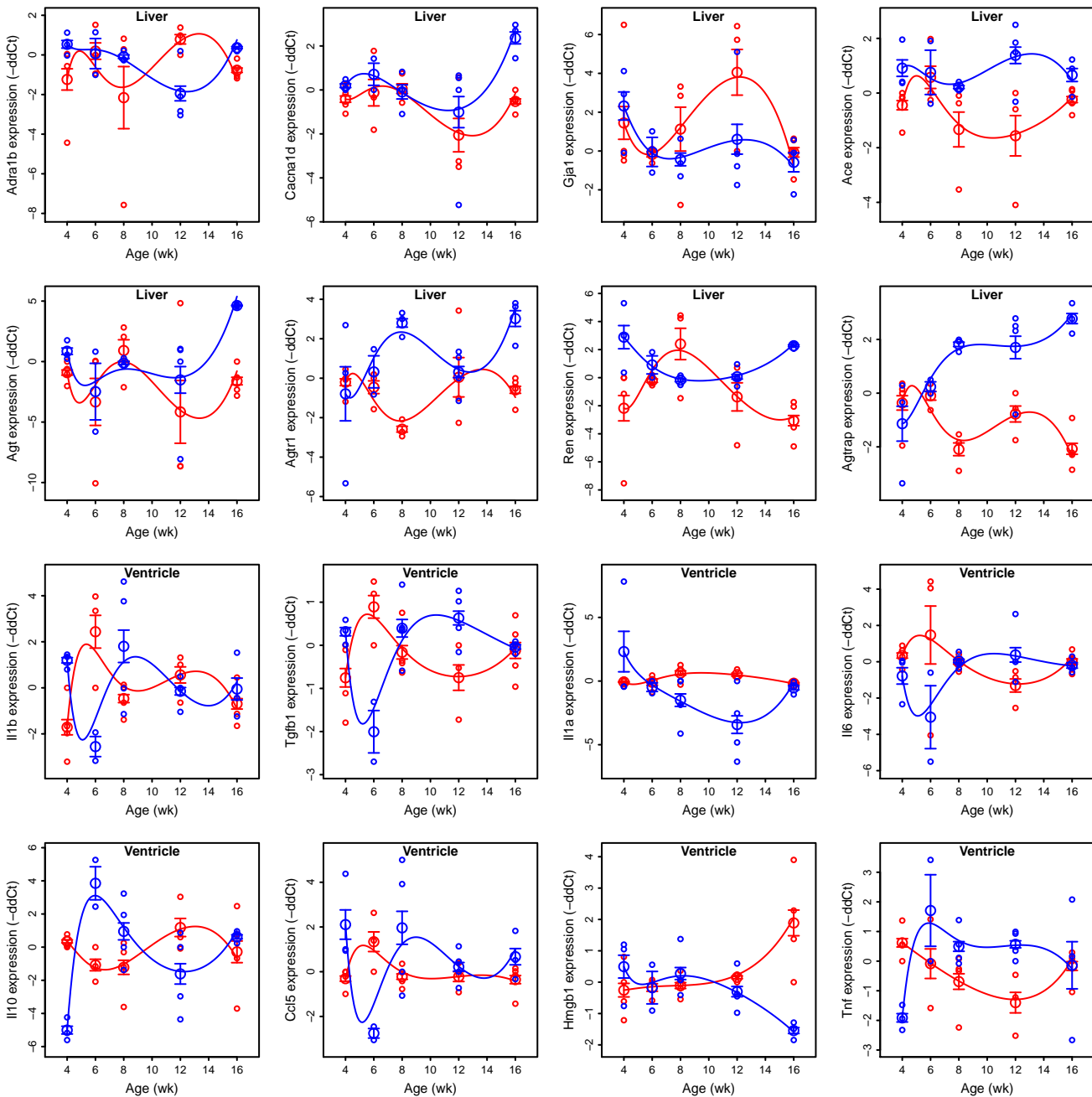

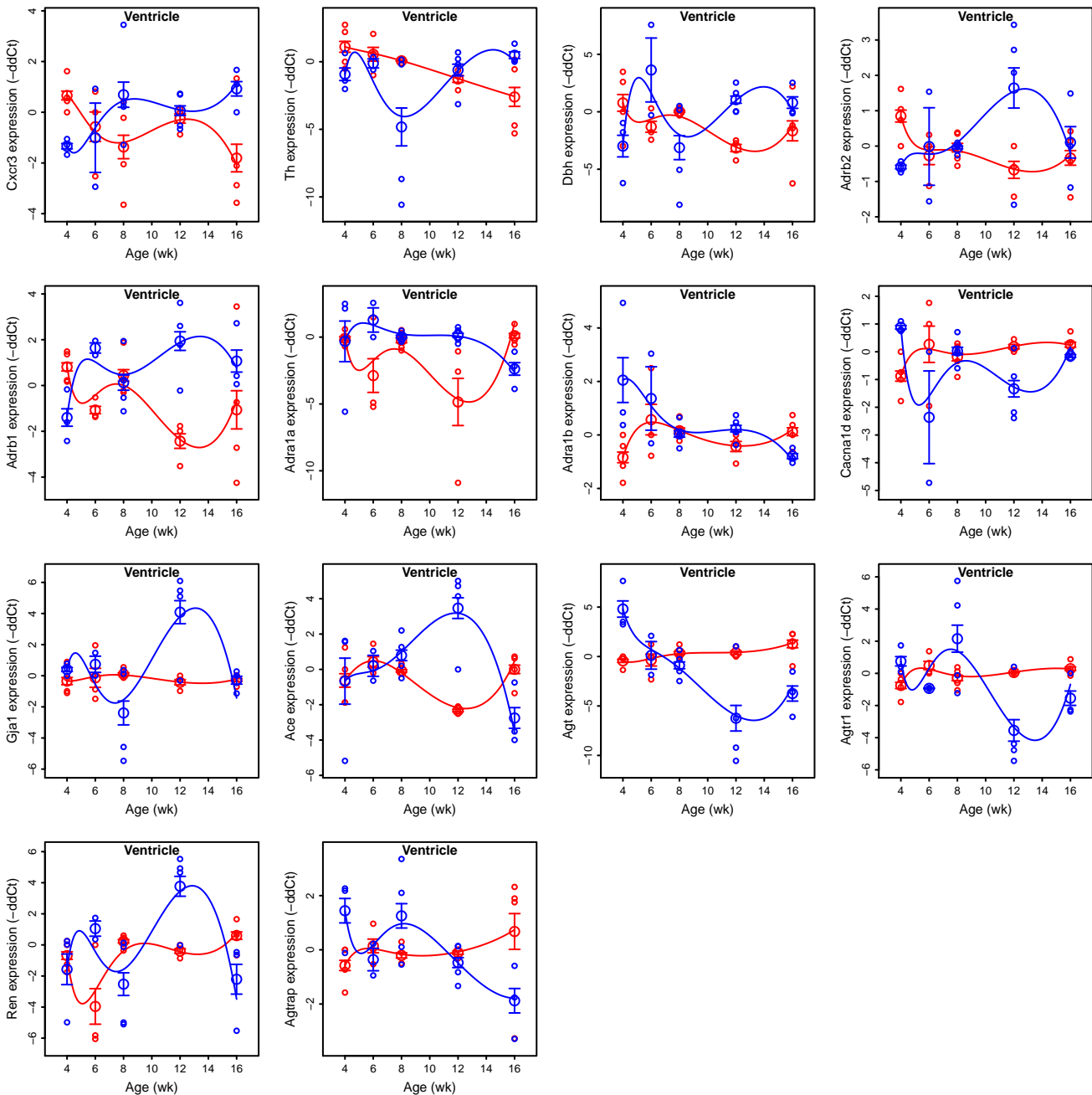

Supplement: S3 File — (PDF) [file pcbi.1005627.s004.pdf]

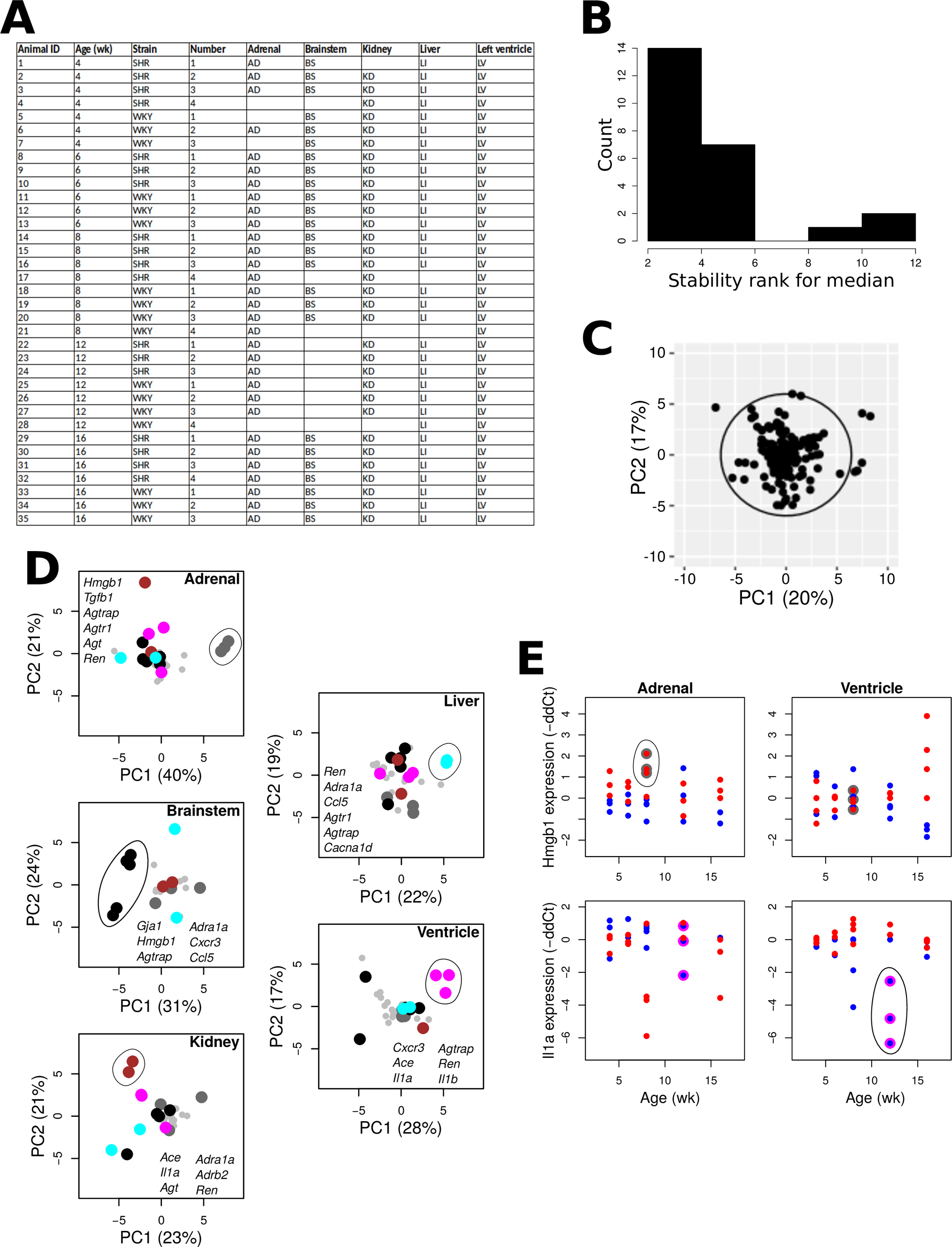

Supplement: S1 Fig — (A) Table detailing the animal sampling and organs utilized in our analysis for each animal. (B) Stability ranks of median expression values were considered for each organ/age combination. For the majority of organ/time combinations, the median expression level was ranked among the most stable (≤ 12/22), in comparison with the stability levels for individual genes. (C) PCA was applied to the entire data set (all genes/organs) and plotted along with the variability accounted for by the first two PCs. The smooth circle shows the 99% confidence interval for the mean of a bi-variate Gaussian distribution characterized by the displayed data. Note that this interval contains the majority of the data, and the few value outside of this interval are in close proximity. (D) PCA was implemented separately for each organ. Specific colors refer to the same animals in all plots. For instance, the three gray dots in the Adrenal PCA plot refer to three animals that are relatively distant from the other animal samples in this analysis. However, observation of the PC projections of these specific animals in the PCAs applied to the data from other organs shows that these animal samples are not imposing consistent biases. Panel (E) shows sample expression data labeled as in (D) for animal samples marked in the Adrenal and Ventricle PCAs. (TIF) [file pcbi.1005627.s012.tif]

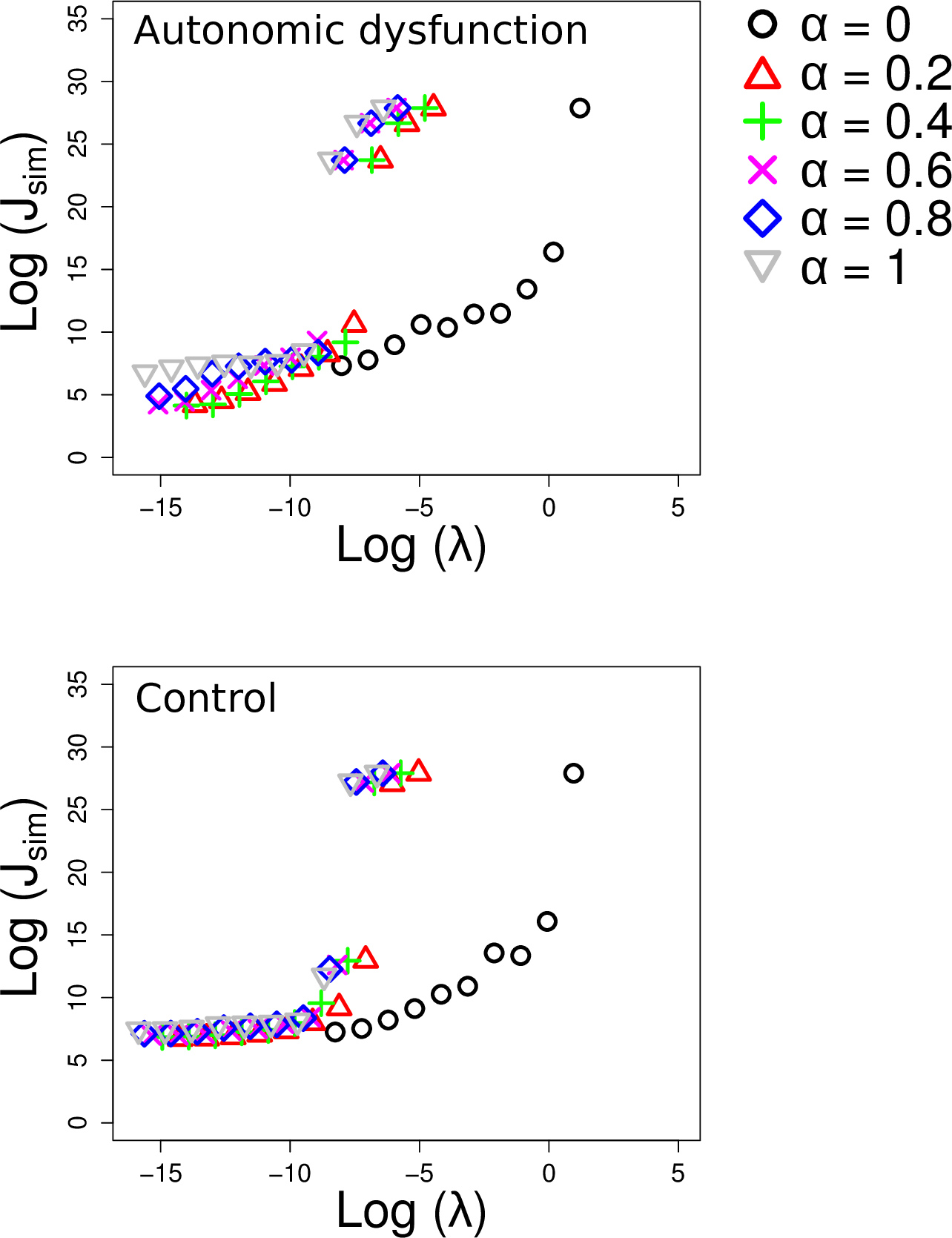

Supplement: S2 Fig — Error between simulated gene expression levels and experimentally measured mean expression values varies minimally with respect to regularization parameters. Log error is plotted with respect to the log λ value for a range of α levels. (TIF) [file pcbi.1005627.s013.tif]

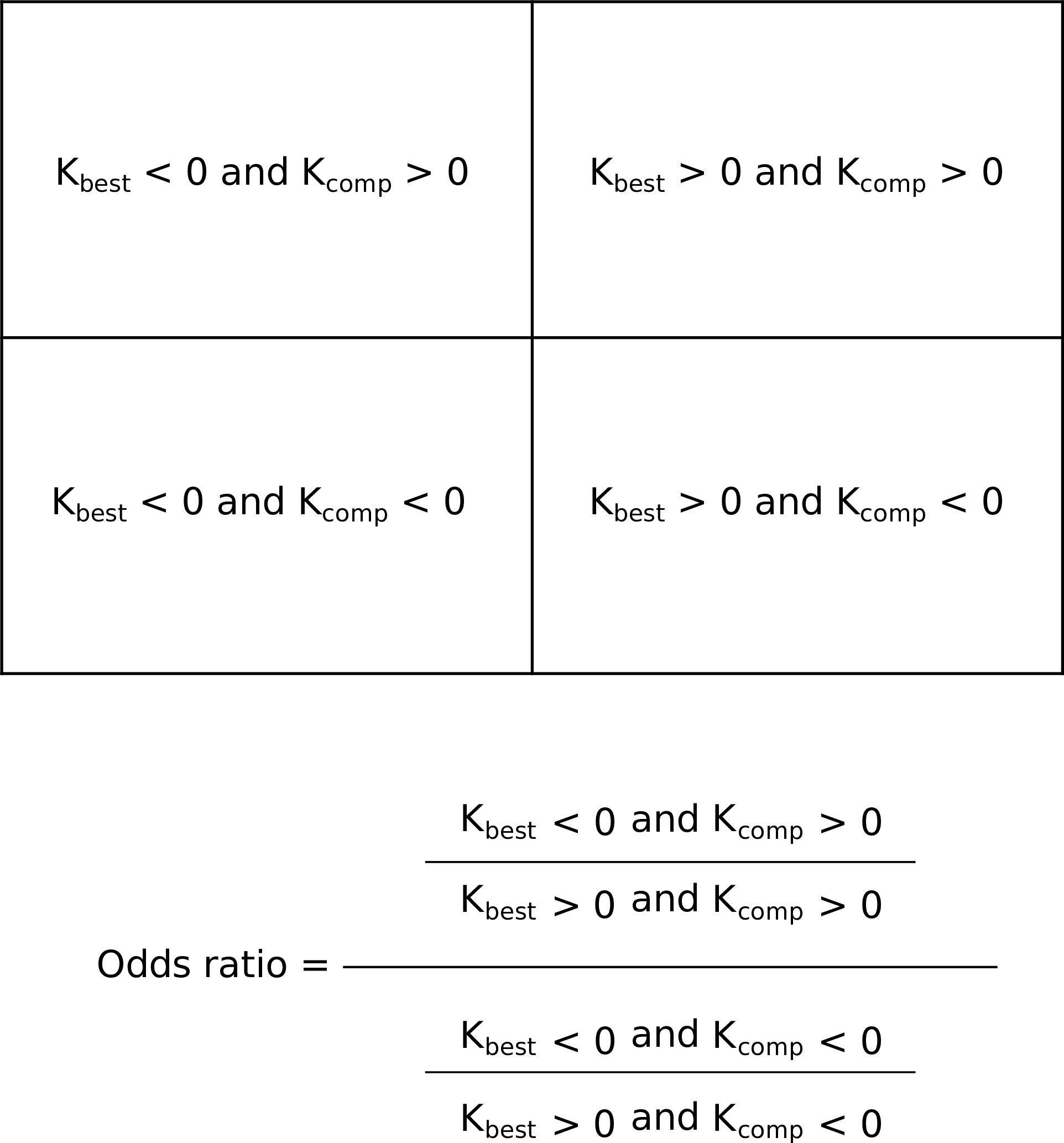

Supplement: S3 Fig — The equation illustrates the computation of the odds ratio based on the contingency table. (TIF) [file pcbi.1005627.s014.tif]

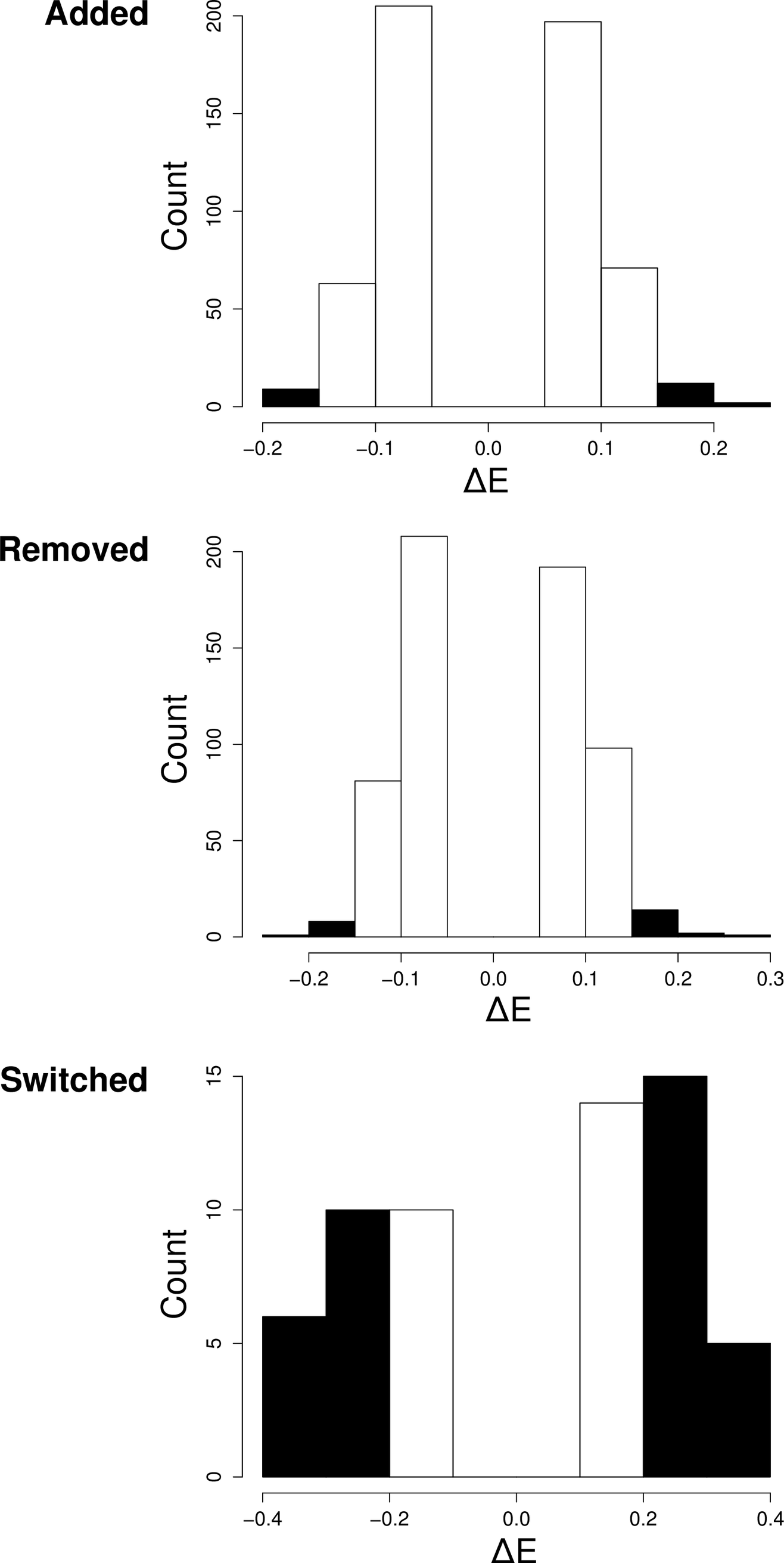

Supplement: S4 Fig — Black bars correspond to edges considered to be differentially regulated in autonomic dysfunction. (TIF) [file pcbi.1005627.s015.tif]

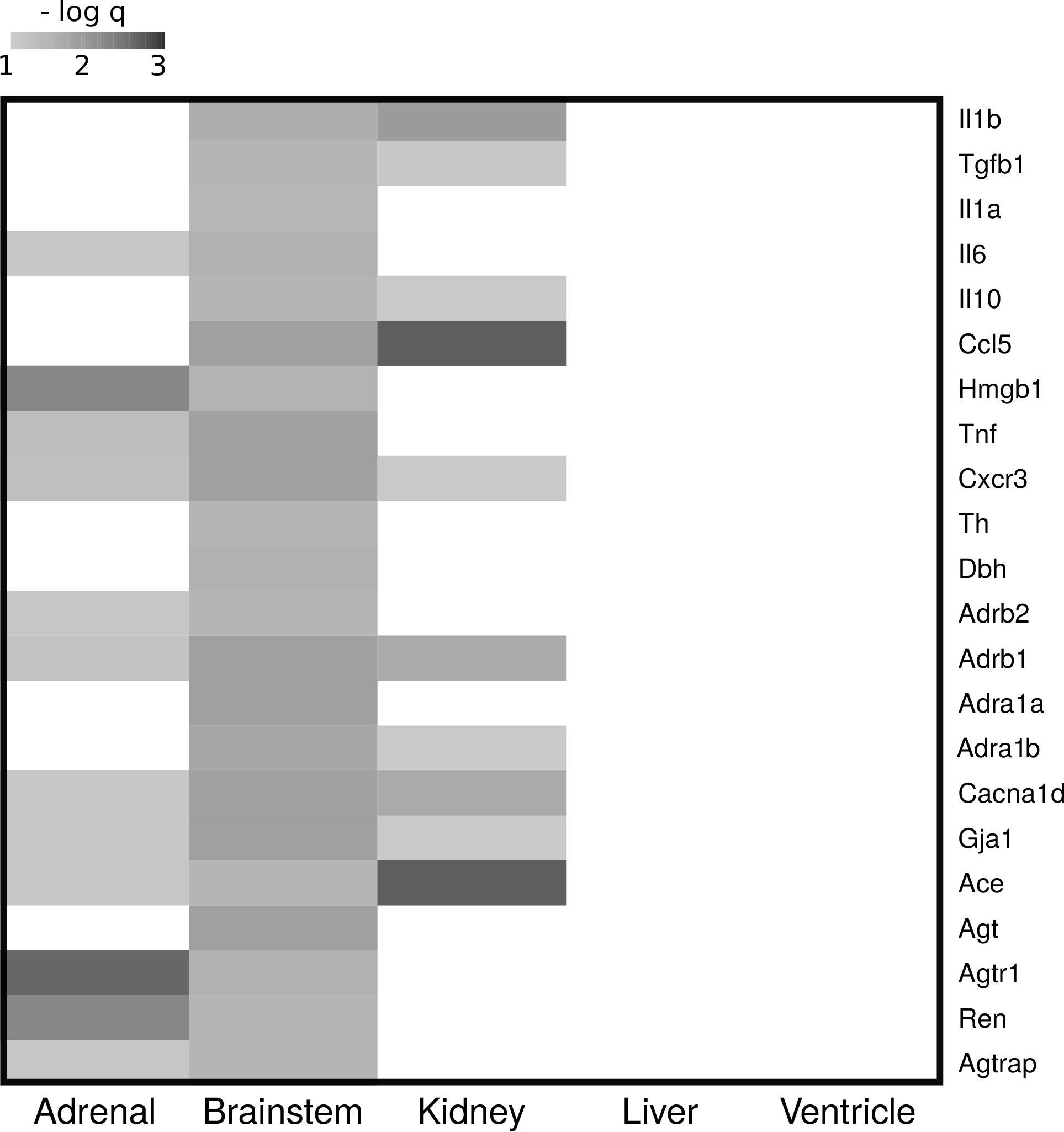

Supplement: S5 Fig — Many genes showed significantly different expression patterns between autonomic dysfunction and control phenotypes (q < 0.1, -log q > 1). (TIF) [file pcbi.1005627.s016.tif]

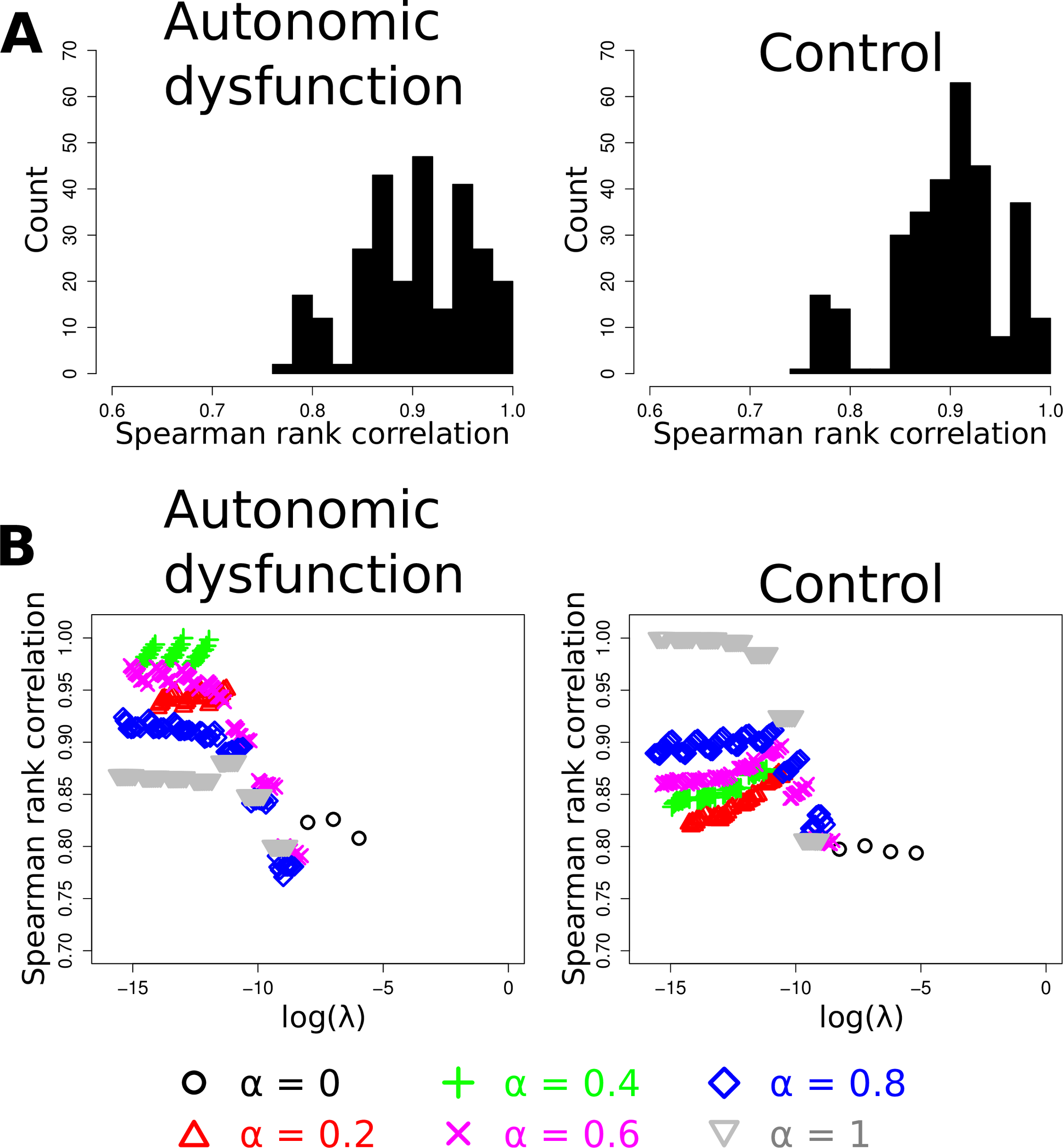

Supplement: S6 Fig — High correlations (> 0.7) between identified networks were observed over an expansive range of regularization parameter space. (A) Spearman rank correlation coefficient histogram and (B) Correlation values as a function of regularization parameter values for λ and α. (TIF) [file pcbi.1005627.s017.tif]

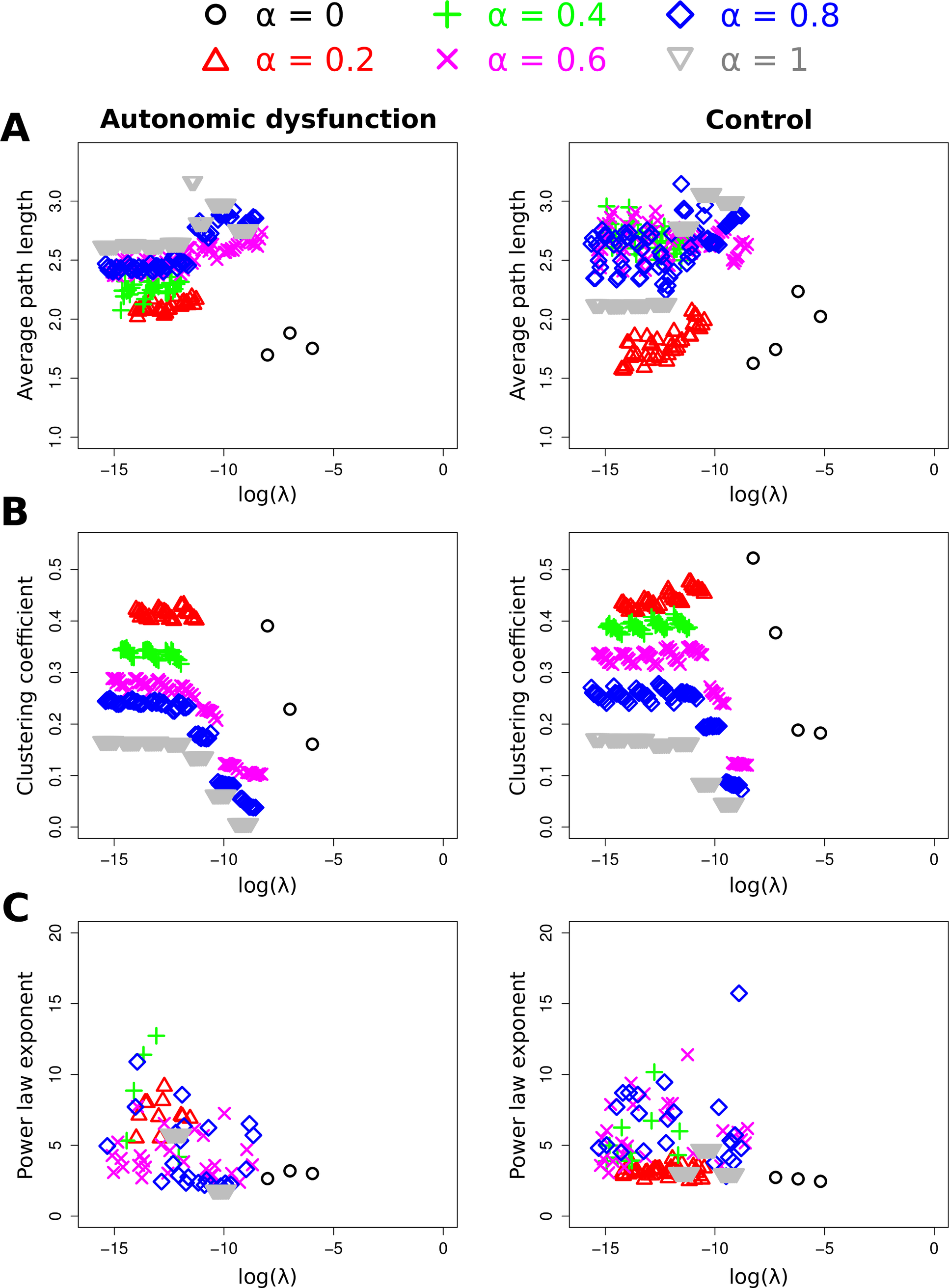

Supplement: S7 Fig — (A) Path length, (B) clustering coefficients, and (C) power law exponents are shown for a range of regularization parameters. (TIF) [file pcbi.1005627.s018.tif]

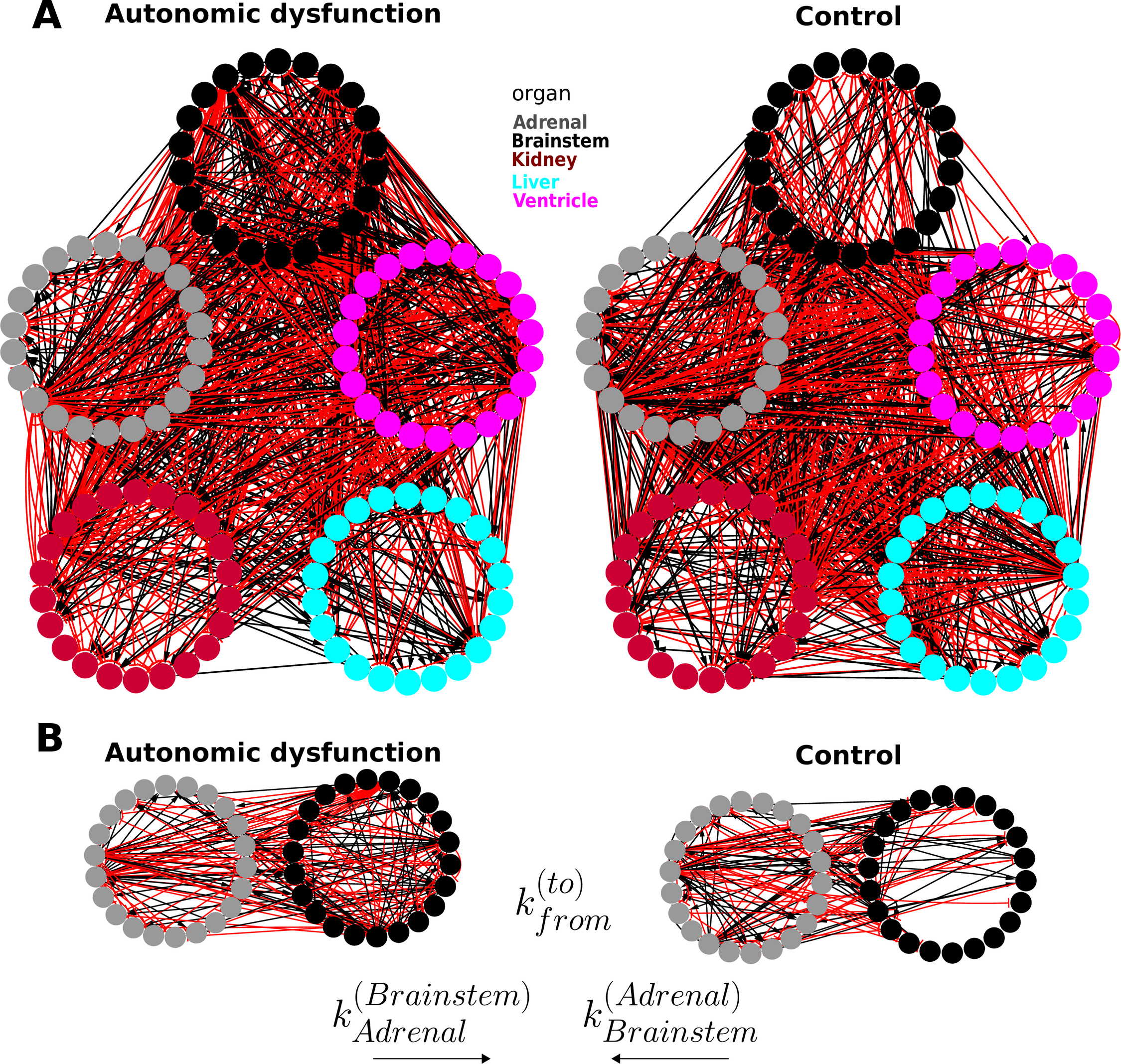

Supplement: S8 Fig — (A) Phenotype-specific multi-organ networks. (B) Subnetworks including interactions between the brainstem and adrenal gland. (TIF) [file pcbi.1005627.s019.tif]

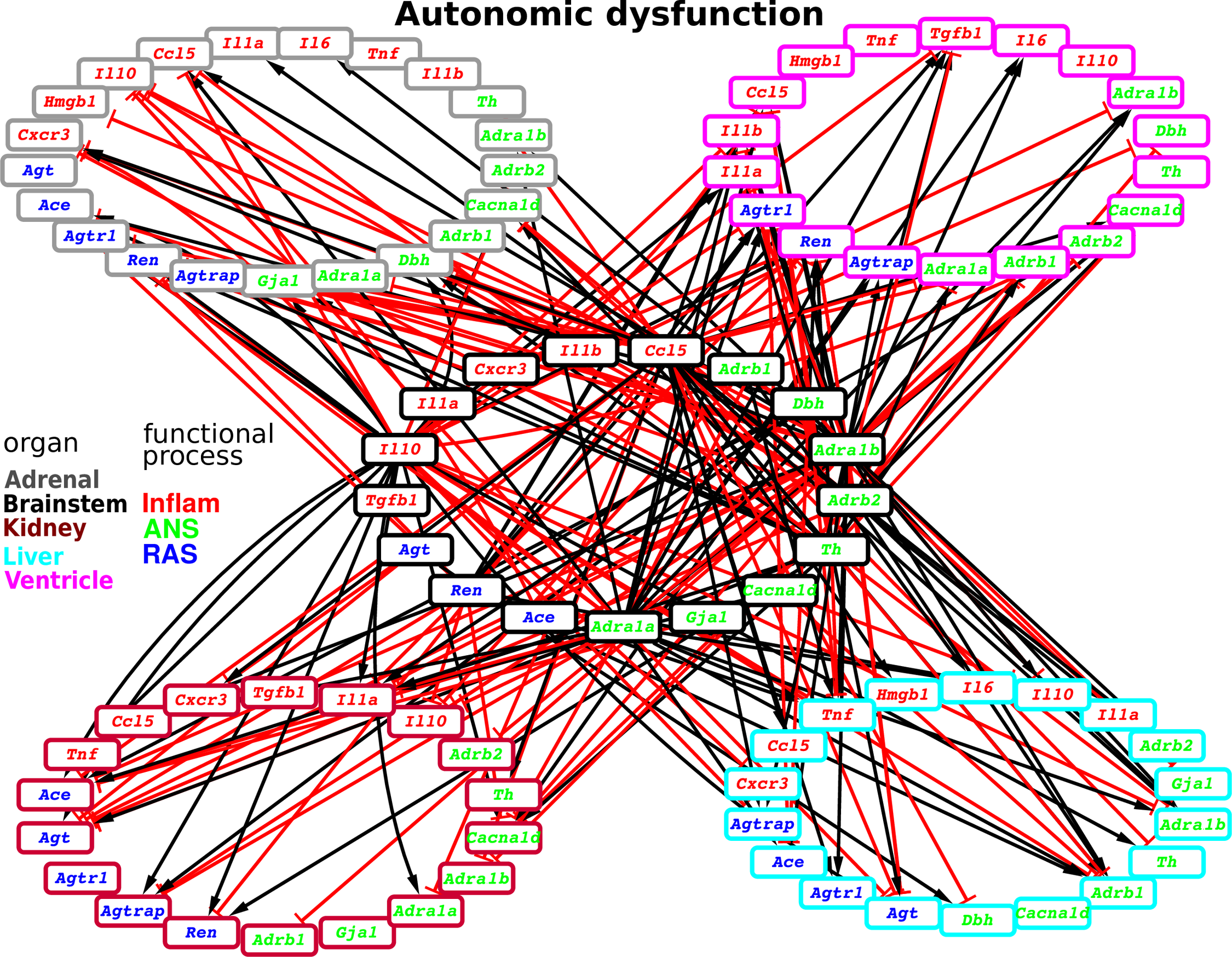

Supplement: S9 Fig — Note that the nodes are organized as in S10 Fig for comparison. (TIF) [file pcbi.1005627.s020.tif]

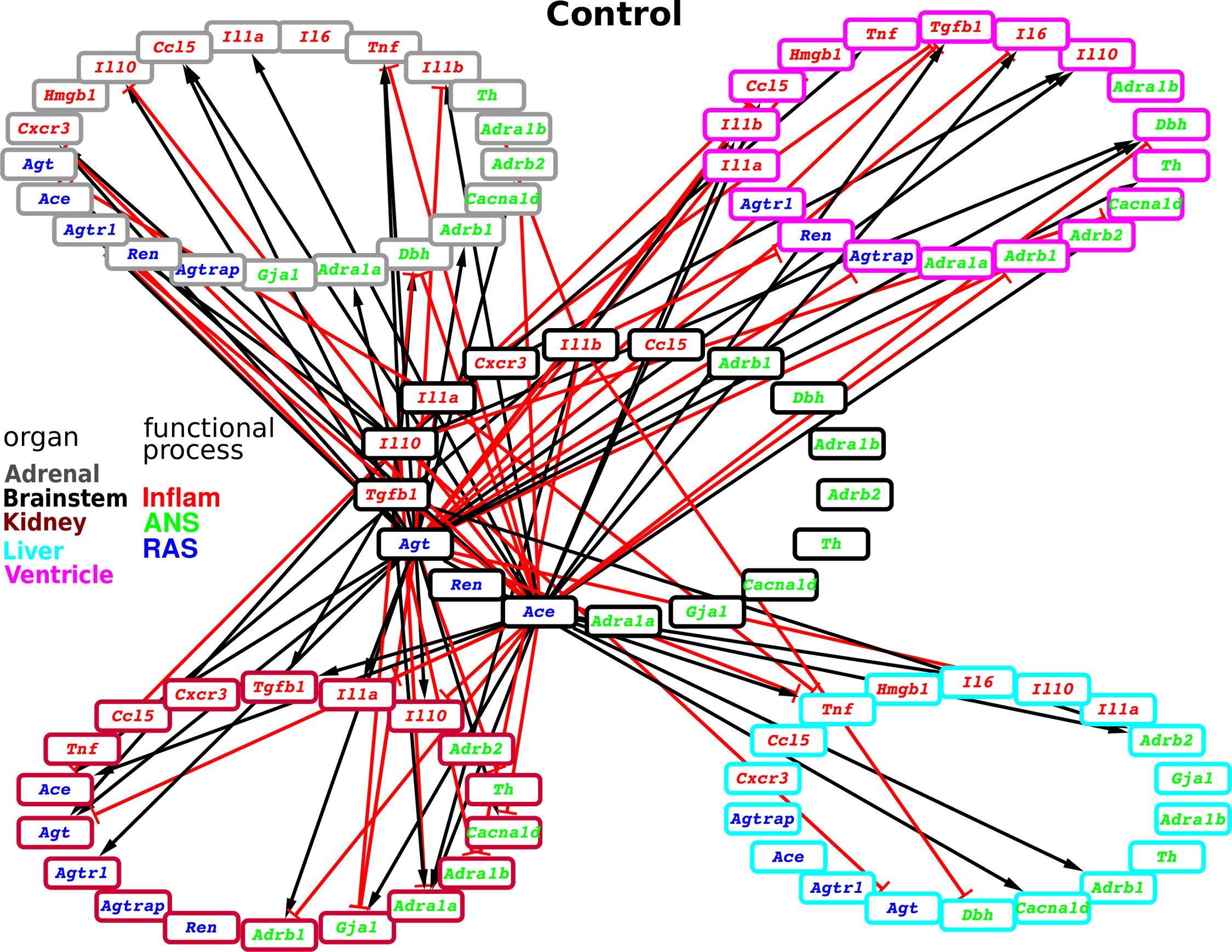

Supplement: S10 Fig — Note that the nodes are organized as in S9 Fig for comparison. (TIF) [file pcbi.1005627.s021.tif]

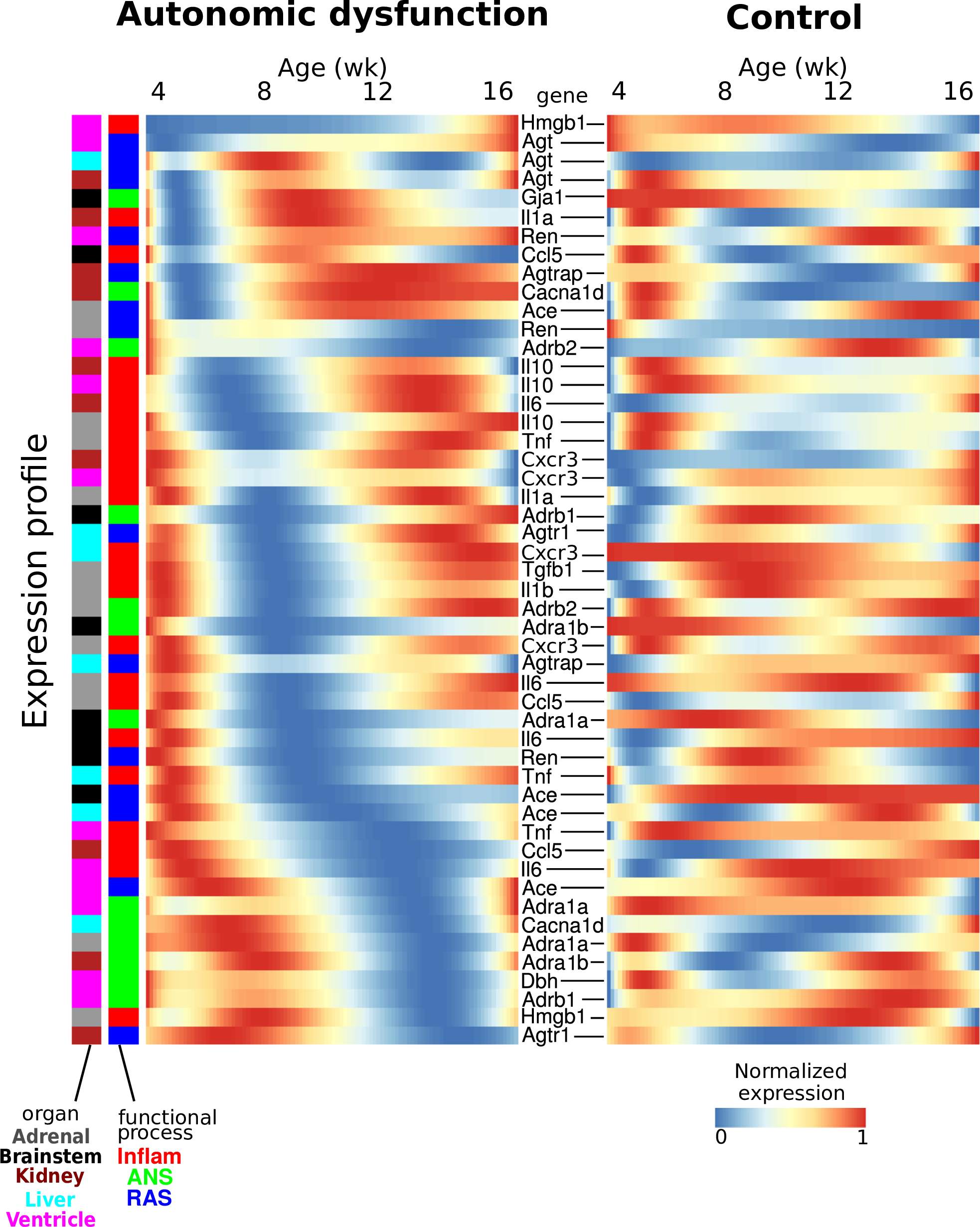

Supplement: S11 Fig — Expression profiles were organized according to the sequence of valleys observed for the autonomic dysfunction phenotype (left). (TIF) [file pcbi.1005627.s022.tif]

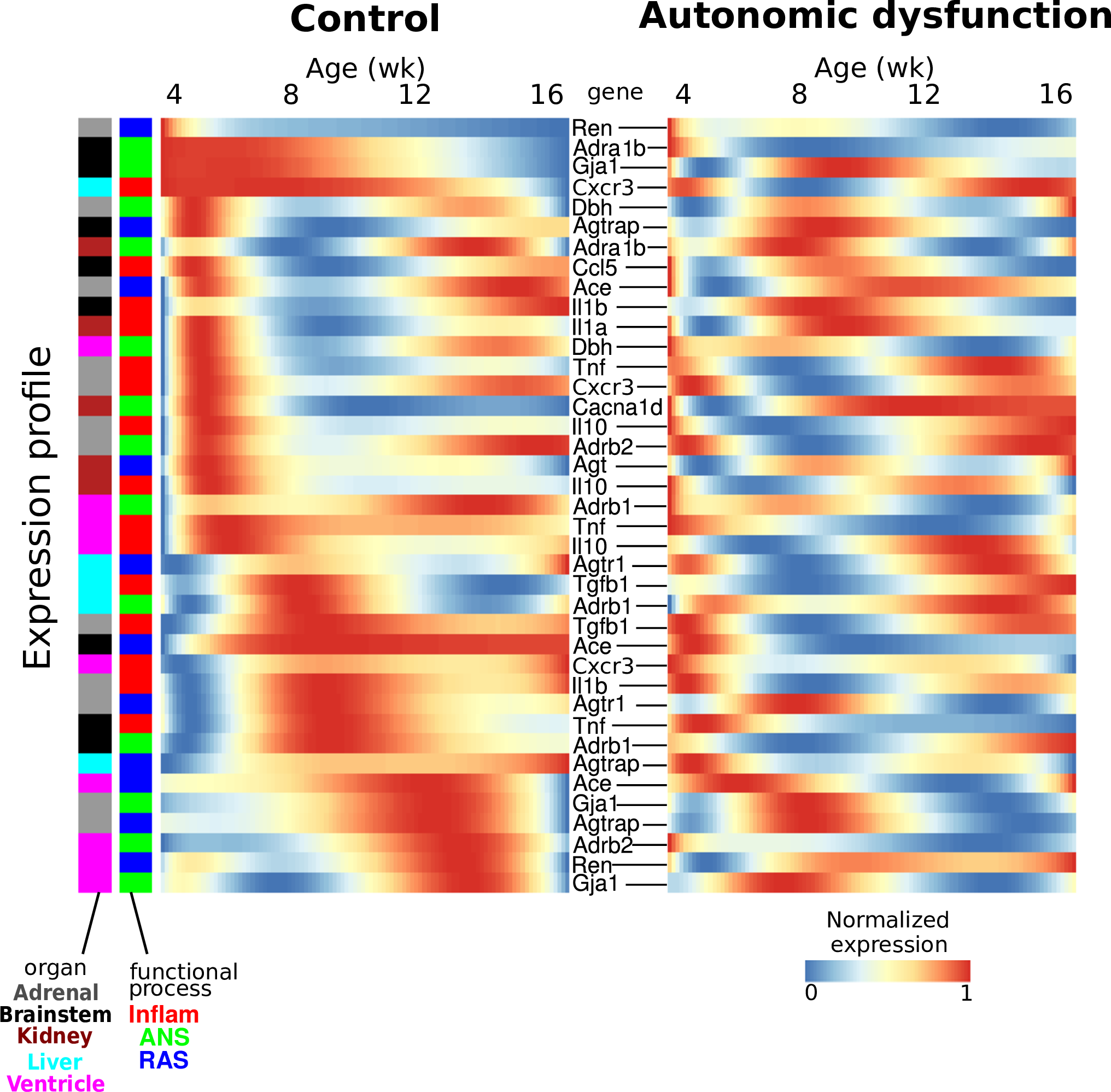

Supplement: S12 Fig — Expression profiles were organized according to the sequence of peaks observed for the control phenotype (left). (TIF) [file pcbi.1005627.s023.tif]

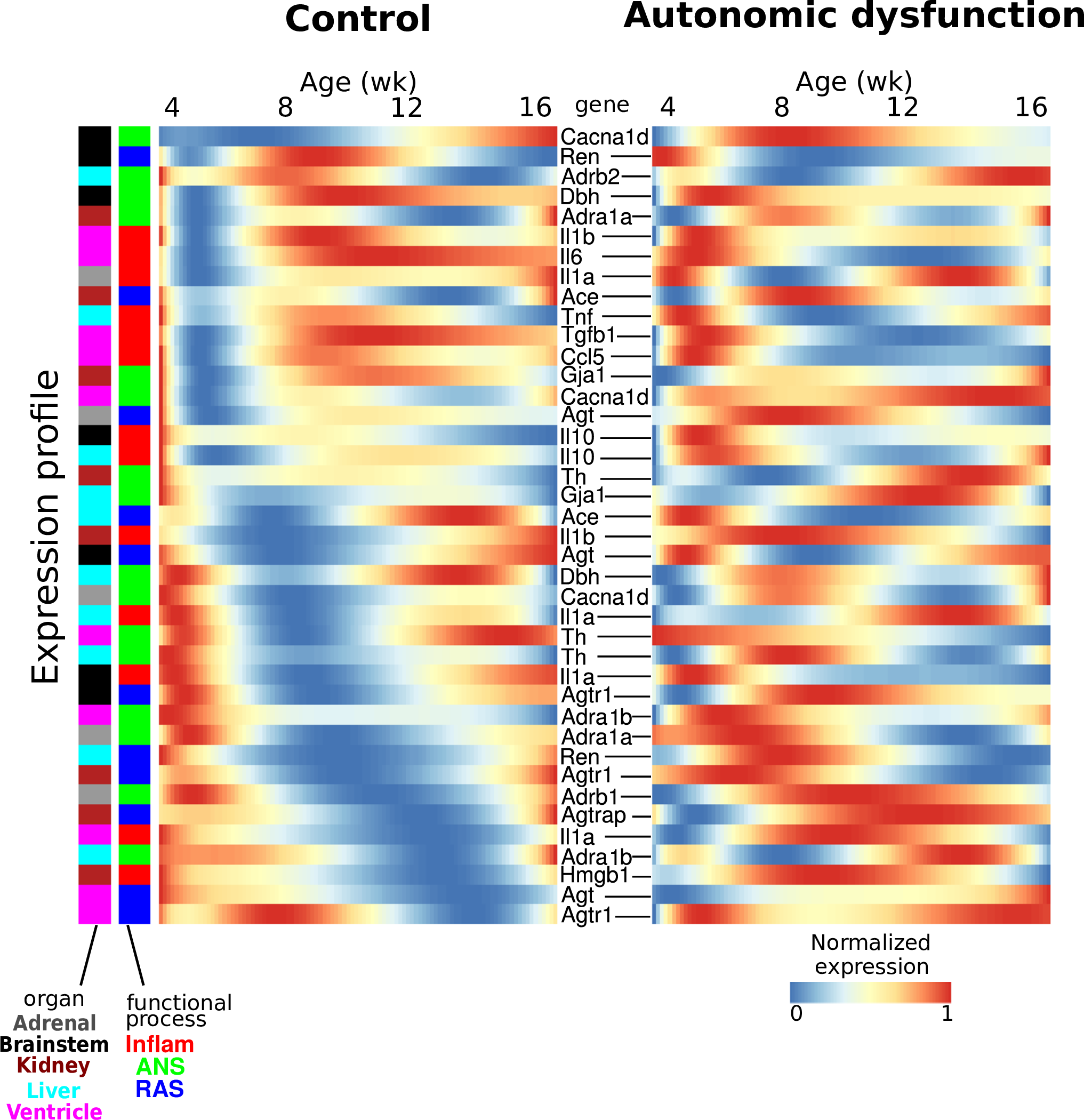

Supplement: S13 Fig — Expression profiles were organized according to the sequence of valleys observed for the control phenotype (left). (TIF) [file pcbi.1005627.s024.tif]

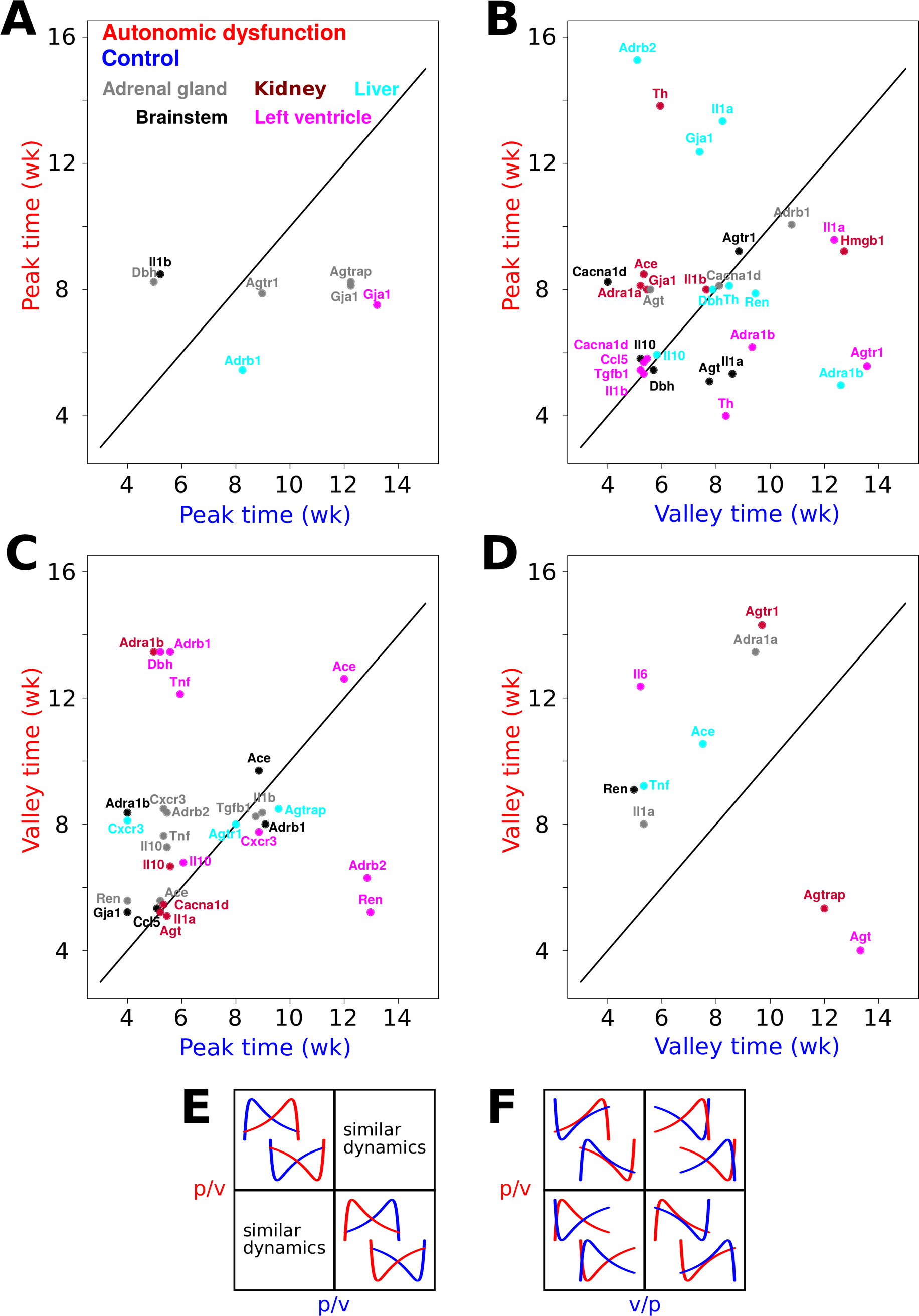

Supplement: S14 Fig — Genes are shown that exhibit (A) peaks in both phenotypes, (B) peaks in autonomic dysfunction but valleys for the control phenotype, (C) valleys for autonomic dysfunction but peaks for the control phenotype, and (D) valleys for both phenotypes. Straight black lines correspond to the unity line. (E) Conceptual overview of the profiles observed in panel (A, peaks on both axes) and panel (D, valleys on both axes). The top left quadrant of panel (E) shows two sets of profiles: in the first, the control profile shows an early peak while the disease profile shows a late peak; in the second, the control shows an early valley and the disease profile shows a late valley. Respectively, these two profiles in the upper left quadrant of panel (E) correspond to the upper left quadrants of panels (A) and (D). These sets of profiles correspond to preserved waveforms but temporal shifts between the expression in control versus disease phenotypes. Panel (F) can be interpreted as for panel (E). Each quadrant of (F) exhibits pairs of dynamic profiles corresponding to either panel (B, top pair) or (C, bottom pair). The extreme off-diagonal profiles depict instances in which the dynamics patterns are inverted for disease relative to control. (TIF) [file pcbi.1005627.s025.tif]

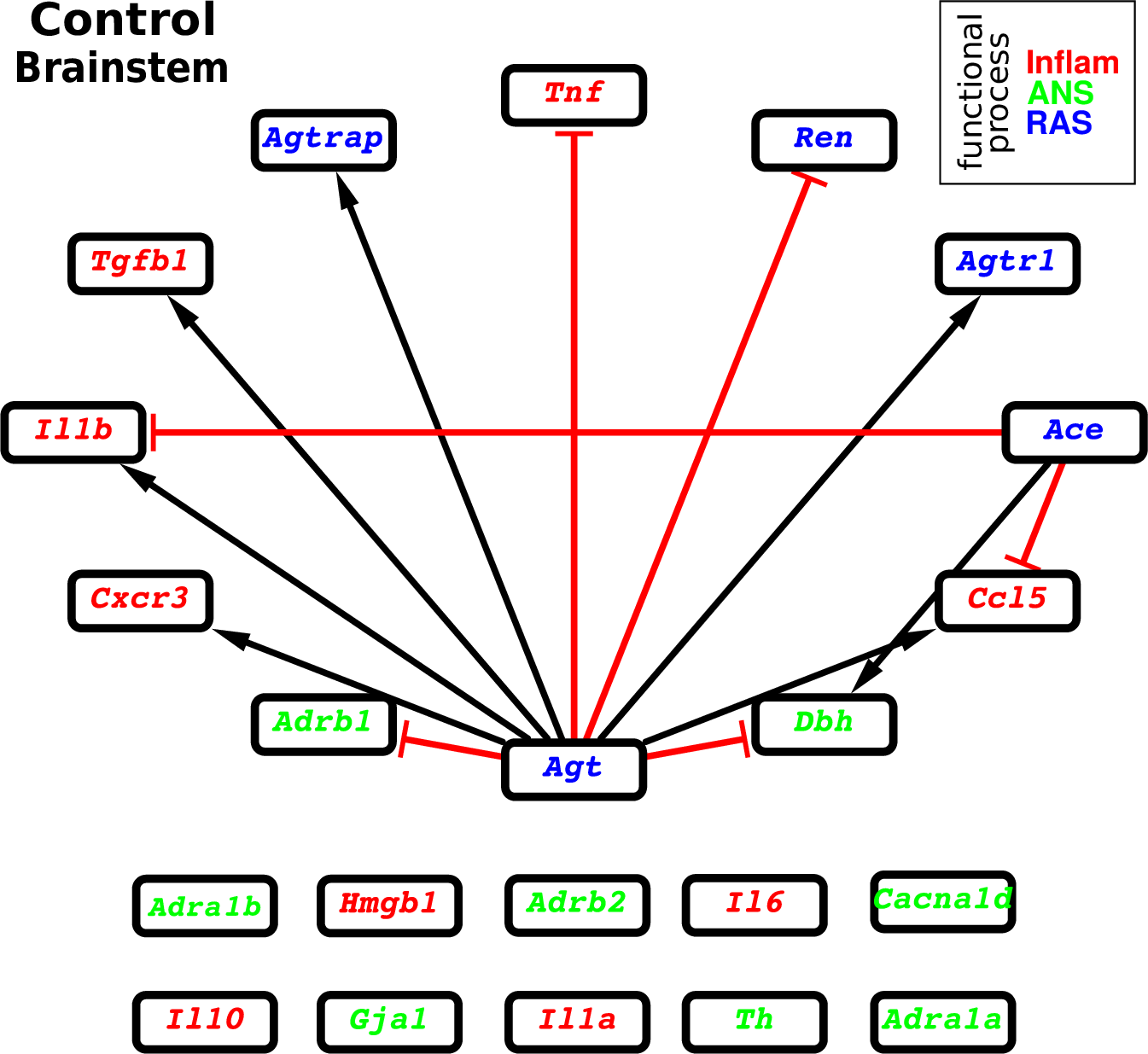

Supplement: S15 Fig — This representation is shown for comparison with main text Fig 7A. (TIF) [file pcbi.1005627.s026.tif]

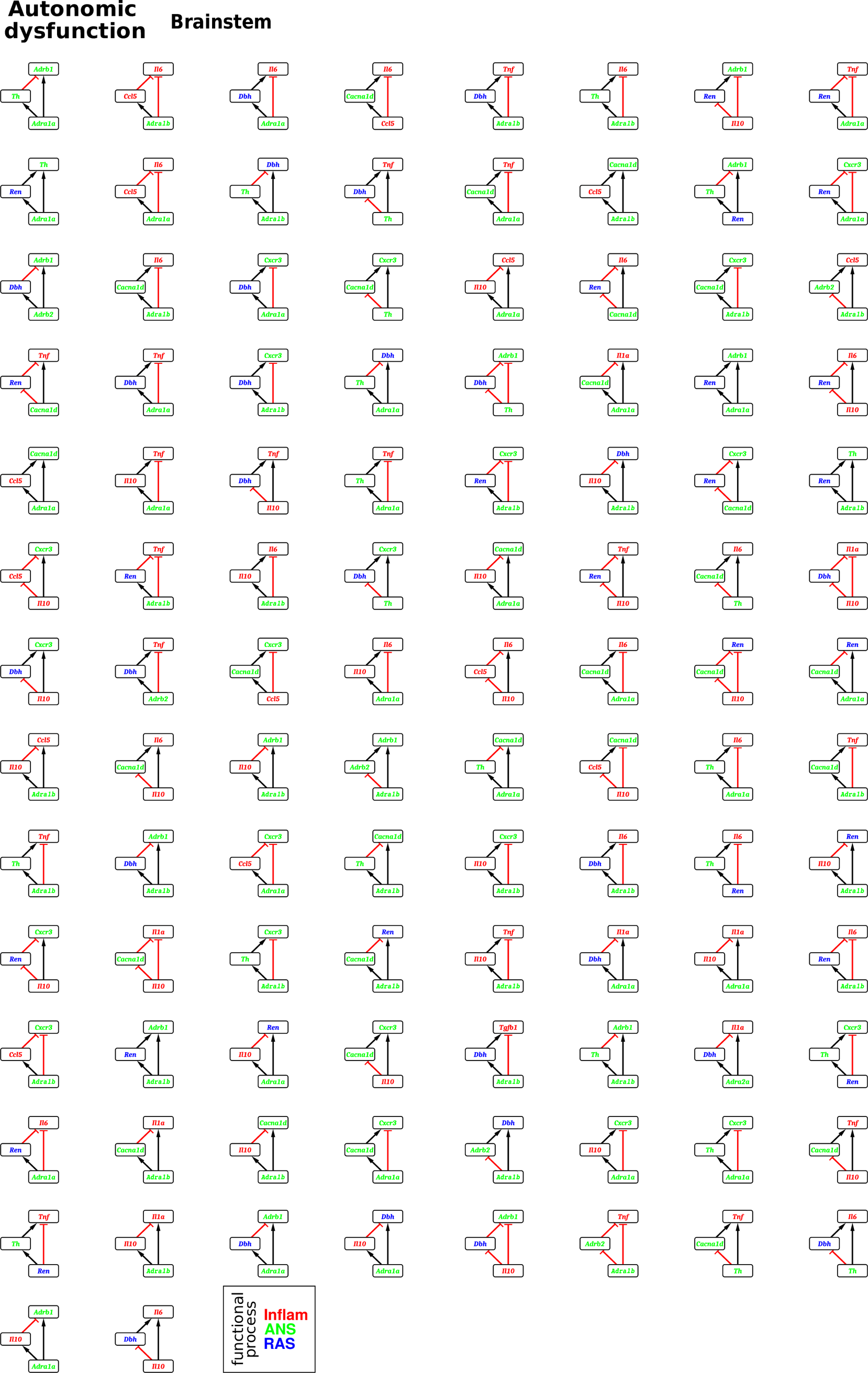

Supplement: S16 Fig — All three node feedforward motifs were identified by motif analysis. (TIF) [file pcbi.1005627.s027.tif]

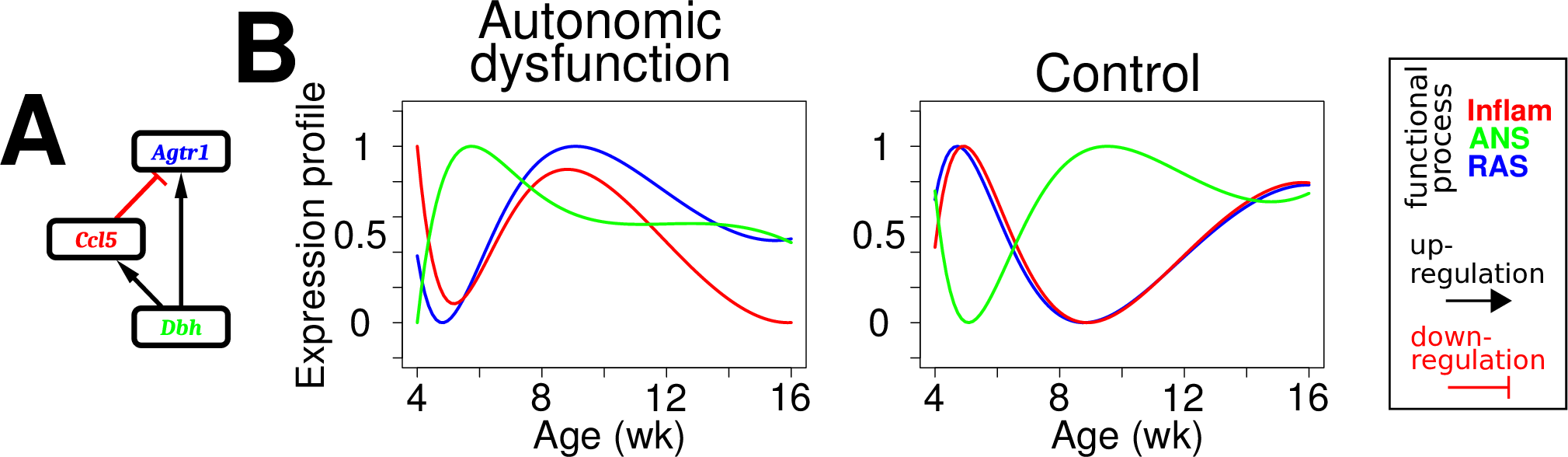

Supplement: S17 Fig — (A) Network motif and (B) simulation traces. (TIF) [file pcbi.1005627.s028.tif]

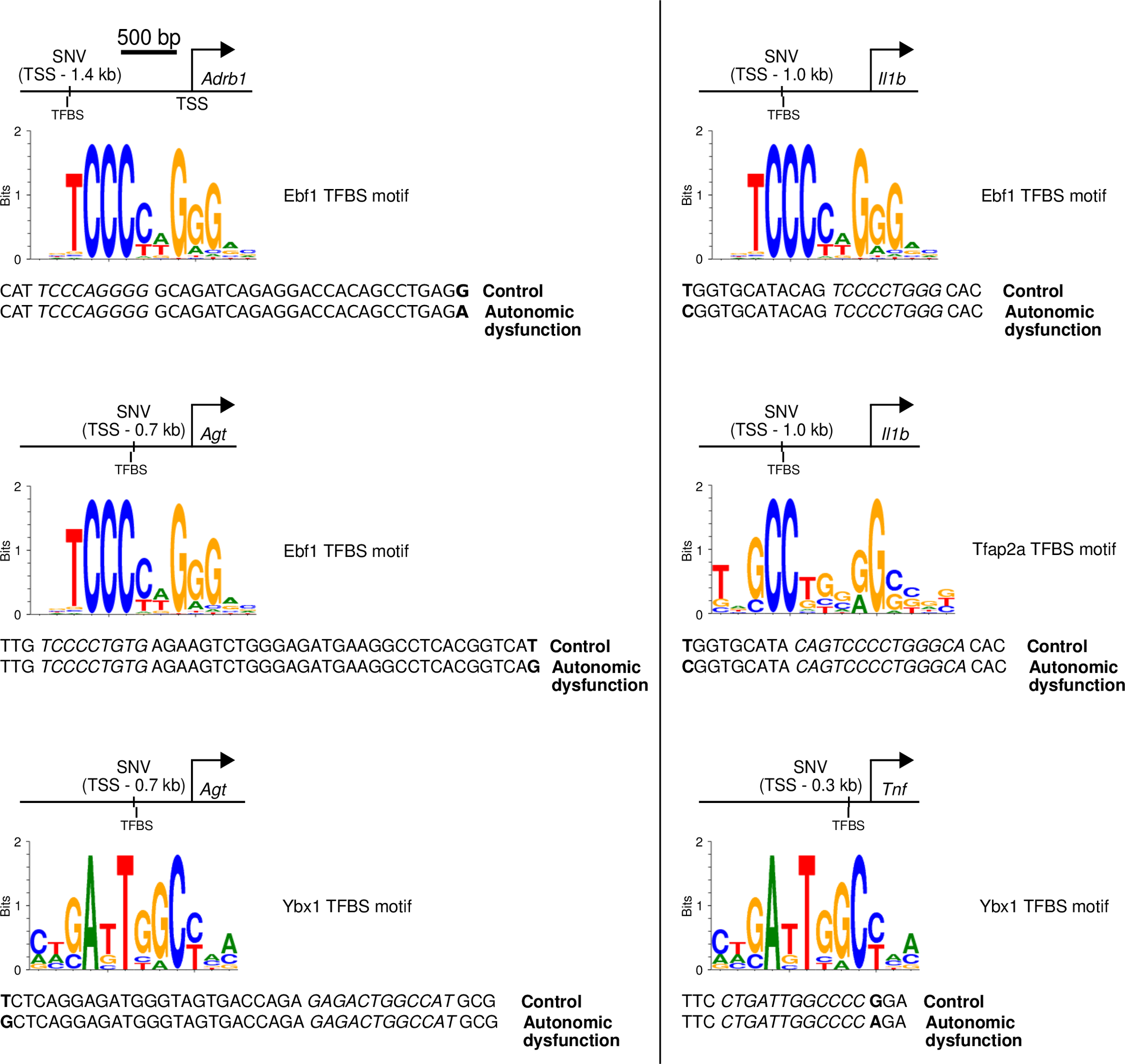

Supplement: S18 Fig — Motif signatures for transcription factors and spatial proximities between TFBSs, TSSs, and SNVs. (TIF) [file pcbi.1005627.s029.tif]
